# Gut Microbiota Signatures Associate with Chemotherapy-Related Adverse Events in Breast Cancer Patients

**DOI:** 10.3390/cancers17233783

**Published:** 2025-11-26

**Authors:** Jiun-Wei Hsu, Ying-Wen Su, Chung-Hsin Tsai, Chi-Chan Lee, Horng-Woei Yang, Jie-Jen Lee, Hsi-Hsien Hsu, Fang Lee, Po-Sheng Yang

**Affiliations:** 1 Department of General Surgery, MacKay Memorial Hospital, Taipei 104217, Taiwan; 2 Department of Medicine, Mackay Medical University, New Taipei 252005, Taiwan; 3 Department of Medical Oncology, MacKay Memorial Hospital, Taipei 104217, Taiwan; 4 Department of Medical Research, MacKay Memorial Hospital, New Taipei City 251404, Taiwan; 5 Department of Colorectal Surgery, MacKay Memorial Hospital, Taipei 104217, Taiwan

**Keywords:** gut microbiota, chemotherapy toxicity, breast cancer, adverse events, hematologic toxicity, hepatotoxicity, biomarkers, 16S rRNA sequencing, butyrate-producing bacteria, microbiome diversity

## Abstract

**Background/Objectives**: Chemotherapy-induced adverse events significantly impact cancer treatment outcomes and patient quality of life. Emerging evidence suggests that gut microbiota composition may influence individual susceptibility to chemotherapy toxicity. This study investigated associations between gut microbiome alterations and specific chemotherapy-related adverse events in breast cancer patients undergoing cancer treatment. **Methods**: We conducted a prospective cohort study of 81 breast cancer patients receiving chemotherapy between May 2018 and October 2022. Gut microbiome composition was analyzed using 16S rRNA sequencing across multiple taxonomic levels. Alpha diversity (Chao1 estimator) and beta diversity (PERMANOVA) analyses were performed to assess microbial richness and community structure. Linear discriminant analysis effect size (LEfSe) identified differentially abundant taxa, with Cohen’s d effect sizes calculated with 95% confidence intervals and false discovery rate correction applied for multiple comparisons. **Results**: Significant microbiome-toxicity associations were identified across 42 taxa (FDR q < 0.05, effect sizes d = −1.60 to +1.67). Severe anemia demonstrated the strongest associations, with large effect size enrichment of Eubacteriaceae (d = 1.38), Anaerofilum (d = 1.42), and Eubacterium (d = 1.38). Severe neutropenia was characterized by depletion of butyrate-producing bacteria, particularly Eubacterium_hallii_group (d = −1.08). Thrombocytopenia showed dramatic depletion of Eubacterium limosum (53-fold reduction, d = −1.60). Hepatotoxicity (ALT elevation) was associated with depletion of Eubacterium_hallii_group (d = −0.99) and Burkholderia-Caballeronia-Paraburkholderia (d = −1.19). Hand-foot syndrome exhibited significantly elevated microbial diversity with depletion of Lachnospiraceae taxa. Most adverse events showed enrichment rather than depletion patterns, with 54.8% of significant associations demonstrating medium to large effect sizes. **Conclusions**: Specific gut microbiota signatures associated with distinct chemotherapy-related adverse events, suggesting potential microbiome-based biomarkers for toxicity prediction and personalized cancer treatment strategies.

## Simple Summary

Chemotherapy saves lives but often causes debilitating side effects, including blood cell depletion, liver damage, nausea, and skin reactions. Recent studies suggest that gut bacteria may influence how patients respond to cancer treatment. We examined gut microbiome composition in 81 breast cancer patients undergoing chemotherapy to identify bacterial patterns associated with treatment side effects. Our analysis revealed that specific bacterial families and genera were significantly linked to particular toxicities. Patients with severe anemia showed enrichment of certain bacteria, while those with severe blood cell depletion had reduced levels of beneficial butyrate-producing bacteria. Liver toxicity, digestive problems, and hand-foot syndrome each displayed distinct bacterial signatures. These findings suggest that gut microbiome profiles could potentially serve as predictive biomarkers, enabling clinicians to identify high-risk patients and implement preventive strategies to reduce chemotherapy-related complications and improve patient quality of life during cancer treatment.

## 1. Introduction

Breast cancer represents the most prevalent malignancy among women worldwide, with approximately 2.3 million new cases diagnosed annually, accounting for 11.7% of all cancer cases and resulting in 685,000 deaths [1]. The disease affects one in eight women during their lifetime and represents the leading cause of cancer-related mortality in females [2]. Chemotherapy remains a cornerstone of breast cancer treatment across various disease stages. Overall chemotherapy utilization across all subtypes was 53.17% in a separate NCDB cohort of 414,528 patients [3]. Breast cancer is characterized by distinct molecular subtypes based on receptor expression patterns—hormone receptor-positive (HR+), human epidermal growth factor receptor 2-positive (HER2+)—which fundamentally influence treatment approaches and prognosis. Chemotherapy usage rates differ substantially by breast cancer molecular subtype. In the National Cancer Database (2010–2015), among 315,264 patients receiving chemotherapy, triple-negative breast cancer had the highest neoadjuvant chemotherapy rates (increasing from 19.5% to 33.7%), followed by HER2-positive subtypes (HR-/HER2+: 21.5→39.8%; HR+/HER2+: 17.0→33.7%) [4]. In contrast, HR+/HER2- tumors showed lower rates (13.0→16.8%). For HR+/HER2- disease specifically, 78% received adjuvant chemotherapy versus 22% who received neoadjuvant chemotherapy. Common regimens typically comprise anthracyclines (doxorubicin, epirubicin), alkylating agents (cyclophosphamide), and taxanes (paclitaxel, docetaxel) [5,6]. Doxorubicin exerts anti-tumor effects through DNA intercalation, topoisomerase II inhibition, and free radical production [7]. While chemotherapy has significantly improved survival rates, clinical application faces substantial challenges. Response rates remain unpredictable due to inherent or acquired tumor resistance, and chemotherapy-induced toxicity occurs frequently and can be severe, with gastrointestinal adverse events affecting 40–100% of patients depending on the regimen [8]. These side effects may necessitate dose reductions or treatment discontinuation, thereby compromising therapeutic efficacy and patient quality of life [9].

The human gut microbiota comprises a complex ecosystem of trillions of microbial cells, predominantly bacteria from the Firmicutes and Bacteroidetes phyla [10]. Each individual harbors a distinct gut microbial composition influenced by age, diet, medications, and other factors [11]. This microbial diversity plays essential roles in nutrient absorption, production of short-chain fatty acids (SCFAs), immune homeostasis, and pathogen protection [12]. Disruption of this balance results in dysbiosis—characterized by loss of microbial diversity and increased pathogenic microorganisms—which has been implicated in inflammatory bowel disease, obesity, and cancer [13,14].

Emerging evidence demonstrates that both gut and tumor microbiota profoundly influence anticancer therapy outcomes through diverse mechanisms: drug metabolism, modulation of drug absorption, alteration of host gene expression, immunomodulation, production of bioactive metabolites, and drug bioaccumulation [15,16]. Studies demonstrate that gut microbiota influences the efficacy of HER2-targeted therapy, immune checkpoint inhibitors, and chemotherapy regimens [17,18]. For instance, breast cancer patients responding to anthracycline-based regimens showed enrichment of bacteria producing SCFAs, including Clostridium, Faecalibacterium, and Lactobacillus [19]. Neoadjuvant chemotherapy shifts breast tumor microbiota populations, with increased Pseudomonas and decreased Streptococcus correlating with treatment responsiveness and metastatic progression [20].

The gut microbiota plays a crucial role in modulating chemotherapy-induced toxicity in breast cancer patients. Chemotherapy disrupts gut microbial composition and intestinal barrier integrity, potentially facilitating translocation of lipopolysaccharide (LPS) and triggering systemic inflammation [21]. A comprehensive study of 301 breast cancer patients treated with taxane-based chemotherapy revealed significant associations between baseline gut microbiota and toxicities. Severe hematological toxicity and neutropenia were associated with higher Firmicutes abundance, while Synergistales showed protective effects. Gastrointestinal toxicity correlated with elevated Lachnospiraceae, Oscillospiraceae, and Ruminococcaceae [22]. Despite growing evidence linking microbiota composition to chemotherapy outcomes, the precise bacterial species and mechanistic pathways remain incompletely understood. Understanding these microbiota-chemotherapy interactions holds promise for developing personalized therapeutic strategies through probiotics, prebiotics, dietary interventions, or fecal microbiota transplantation to optimize treatment outcomes and improve quality of life for breast cancer patients [23].

Given that chemotherapy-related toxicities remain a major clinical challenge affecting treatment adherence, quality of life, and therapeutic outcomes, identifying predictive biomarkers is critically important for personalized cancer care. We designed this prospectively cross-sectional study to investigate gut microbiome associations with chemotherapy-induced adverse effects in breast cancer patients undergoing standardized chemotherapy regimens.

## 2. Materials and Methods

### 2.1. Patient Population

This prospective cross-sectional study was conducted at MacKay Memorial Hospital (MMH), Taipei, Taiwan, with institutional review board approval (IRB No. 19MMHIS061e). Adults (≥20 years) with histologically confirmed stage I-IV breast cancer via core biopsy were recruited through convenience sampling. All participants provided written informed consent, while patients with recurrent breast cancer were excluded. All participants received guideline-concordant systemic chemotherapy based on molecular subtype, disease stage, and clinical risk stratification per institutional protocols aligned with international standards. Treatment decisions incorporated tumor size, nodal status, and molecular markers including HER2 status, hormone receptor expression, and multigene assay results (Oncotype Dx, MammaPrint) where applicable. For HER2-positive disease, chemotherapy with trastuzumab was administered for tumors > 1.0 cm or node-positive disease, with pertuzumab added for metastases > 2 mm and TDM-1 reserved for residual disease following neoadjuvant therapy. Triple-negative breast cancer received chemotherapy for tumors > 0.5 cm or with nodal involvement, with capecitabine, olaparib (BRCA mutation carriers), or pembrolizumab administered for residual disease after neoadjuvant therapy. In hormone receptor-positive/HER2-negative disease, chemotherapy decisions in node-negative or limited node-positive cases were guided by multigene assay risk stratification, though pN2/pN3 status mandated chemotherapy regardless of assay results, with high-risk patients considered for adjuvant abemaciclib. Neoadjuvant chemotherapy was preferentially used for locally advanced disease (Stage IIIA-IIIC) and considered for HER2-positive or triple-negative tumors ≥ cT2/cN1, particularly when downstaging could enable breast conservation. For metastatic disease, treatment selection balanced efficacy with toxicity: HER2-positive cases received taxane-based chemotherapy with dual HER2-blockade, triple-negative cases received chemotherapy with or without immunotherapy, while hormone receptor-positive disease was treated with endocrine therapy or chemotherapy depending on visceral crisis status and performance status.

### 2.2. Fecal Sample Collection and DNA Extraction

This study employed a cross-sectional design analyzing baseline (pre-treatment) microbiota composition in relation to chemotherapy-related adverse events that occurred during subsequent treatment. We did not perform longitudinal sampling to assess microbiota changes over the course of chemotherapy. Fecal samples were collected upon breast cancer confirmation by core biopsy, prior to initiation of any treatment (chemotherapy, surgery, or radiotherapy), and stored at −20 °C until analysis. Genomic DNA was extracted using the QIAamp Fast DNA Stool Mini Kit (QIAGEN GmbH, Hilden, Germany) according to manufacturer’s instructions with modifications [24]. Briefly, 0.2 g of fecal sample was homogenized with 1 mL InhibitEX buffer (proprietary stool lysis buffer containing reagents to stabilize DNA and inhibit PCR inhibitors) and glass beads using a Precellys homogenizer (Bertin Instruments, Montigny-le-Bretonneux, France) at 4500 beats/min for 2 min. The suspension was heated at 70 °C for 10 min and centrifuged for 1 min to pellet stool particles. The supernatant (600 µL) was transferred to a new 2 mL tube containing 25 µL proteinase K, followed by addition of 600 µL AL buffer (lysis buffer containing guanidinium salts for protein denaturation and DNA stabilization). After incubation at 70 °C for 10 min, 600 µL of 100% ethanol was added and mixed thoroughly. The mixture was filtered through a QIAamp spin column at 13,000 rpm for 1 min, followed by sequential washing with AW1 buffer (washing buffer containing ethanol and chaotropic salts) and AW2 buffer (washing buffer containing ethanol for final purification). DNA was eluted in 100 µL ATE buffer (elution buffer: 10 mM Tris-HCl, pH 9.0, 0.5 mM EDTA), and its concentration and quality were assessed using a NanoDrop 2000 spectrophotometer (Thermo Scientific, Waltham, MA, USA).

### 2.3. 16S rRNA Gene Sequencing

The V3-V4 hypervariable regions of the 16S rRNA gene were amplified for microbial phylogenetic classification. The first PCR amplification was performed in 25 µL reactions containing 50 ng (2.5 µL) of template DNA, 0.2 µM each of V3-V4 forward primer (5′-TCGTCGGCAGCGTCAGATGTGTATAAGAGACAGCCTACGGGNGGCWGCAG-3′) and reverse primer (5′-GTCTCGTGGGCTCGGAGATGTGTATAAGAGACAGGACTACHVGGGTATCTAATCC-3′), and 12.5 µL 2X Kapa HiFi HotStart ReadyMix (KapaBiosystems, Wilmington, MA, USA). PCR conditions were initial denaturation at 95 °C for 3 min; 25 cycles of denaturation at 95 °C for 30 s, annealing at 55 °C for 30 s, and extension at 72 °C for 30 s; followed by final extension at 72 °C for 5 min. Amplified products were purified using Agencourt AMPure XP Reagent beads (Beckman Coulter Inc., Brea, CA, USA).

Index PCR was performed in 50 µL reactions containing 5 µL of purified amplicon, 25 µL 2X Kapa HiFi HotStart ReadyMix, and 5 µL each of Nextera XT Index 1 and 2 primers (Illumina, San Diego, CA, USA). Cycling conditions were: 95 °C for 3 min; 8 cycles of 95 °C for 30 s, 55 °C for 30 s, and 72 °C for 30 s; with final extension at 72 °C for 5 min using an Applied Biosystems 2720 thermocycler (Thermo Fisher Scientific, Carlsbad, CA, USA). The indexed amplicons were purified using AMPure XP beads. Libraries were quantified using KAPA SYBR FAST qPCR Master Mix on a Roche LightCycler 480 system, normalized to 4 nM, and pooled for sequencing on the Illumina MiSeq platform with 2 × 300 bp paired-end chemistry, generating >80,000 reads per sample [25].

Sequence data were processed using QIIME2 software package (version 2017.10). Chimeric sequences were removed using DADA2, and reads were trimmed by 30 and 90 bases at the 3′ end of forward and reverse reads, respectively [26]. Taxonomic classification was performed using a Naïve Bayes classifier trained on the Greengenes13.8 database with a 99% OTU threshold [27]. The resulting taxonomic assignments were classified at seven levels (kingdom, phylum, class, order, family, genus, and species) using the NCBI database.

### 2.4. Adverse Event Assessment

Adverse events were graded according to CTCAE version 5.0. Hematologic toxicities included anemia (hemoglobin < 10.0 g/dL for Grade 1, <8.0 g/dL for Grade 2–3), neutropenia (ANC < 1500/mm^3^ for Grade 1, <1000/mm^3^ for Grade 2, <500/mm^3^ for Grades 3–4), and thrombocytopenia (platelets < 75,000/mm^3^ for Grade 1, <50,000/mm^3^ for Grade 2, <25,000/mm^3^ for Grades 3–4). Hepatotoxicity was graded by ALT/AST elevations (>ULN to 3.0 × ULN for Grade 1, >3.0–5.0 × ULN for Grade 2, >5.0–20.0 × ULN for Grade 3, >20.0 × ULN for Grade 4). Gastrointestinal toxicities (nausea, oral mucositis, diarrhea), neurological toxicities (peripheral neuropathy), dermatological toxicities (maculopapular rash, hand-foot syndrome), and fatigue were graded based on symptom severity and impact on activities of daily living.

### 2.5. Statistical and Microbial Community Analysis

Microbial analyses were performed using MicrobiomeAnalyst at family, genus, and species levels. Alpha diversity was assessed using the Chao1 index (QIIME 2017.10). Beta diversity was evaluated using Bray–Curtis dissimilarity and visualized by PCoA, with PERMANOVA testing for statistical significance [26].

Taxa absent in ≥5% of participants were excluded. For genera with median relative abundance > 1%, multiple regression analysis examined associations while adjusting for confounders. Non-normal data were normalized using Box–Cox transformation. LEfSe identified differentially abundant features (*p* < 0.05 and Log LDA score > 1.0) stratified by taxonomic level. Linear discriminant analysis effect size (LEfSe) was performed to identify differentially abundant taxa between groups. LEfSe is a computational method that combines statistical significance testing with biological relevance assessment by using the Kruskal–Wallis rank sum test to detect features with significant differential abundance, followed by linear discriminant analysis to estimate the effect size of each differentially abundant feature. An LDA score threshold of > 2.0 was used to identify taxa with biologically meaningful differences [28].

### 2.6. Differential Abundance and Association Analysis

Patients were stratified by adverse event severity grades across twelve comparisons: anemia (grade 2–3 vs. 0–1), severe neutropenia (grade 4 vs. 0–3), thrombocytopenia (grade 1–2 vs. 0), hepatotoxicity (grade 1–3 vs. 0), gastrointestinal toxicities (nausea, mucositis, diarrhea; grade 1–2 vs. 0), peripheral neuropathy (grade 1–2 vs. 0), dermatologic toxicities (rash grade 1–3 vs. 0, hand-foot syndrome grade 1–2 vs. 0), and fatigue (grade 1–2 vs. 0).

Benjamini–Hochberg FDR correction controlled for multiple testing (q < 0.05 significant). Effect sizes were quantified using Cohen’s d, a standardized measure of the difference between two group means expressed in units of standard deviation. Cohen’s d values of 0.2, 0.5, and 0.8 are conventionally interpreted as small, medium, and large effects, respectively. We calculated Cohen’s d with 95% confidence intervals using the effsize package in R and applied false discovery rate (FDR) correction using the Benjamini–Hochberg method to control multiple comparisons, with statistical significance defined as FDR-corrected *p* < 0.05.

### 2.7. Data Visualization

Forest plots visualized effect sizes and 95% CIs for FDR-significant taxa, with positive Cohen’s d indicating enrichment in adverse event groups. A comprehensive heatmap displayed Cohen’s d values across 46 taxa (rows) and 12 adverse events (columns), organized by taxonomic level (family, genus, species). Positive associations were shown in red gradients, negative in blue, with color intensity reflecting effect magnitude. Taxa were color-coded by level (blue: family; green: genus; orange: species). Non-significant cells appeared in gray.

## 3. Results

### 3.1. Patient Demographics and Clinical Characteristics

A total of 81 breast cancer patients who received chemotherapy were enrolled in this study between May 2020 and October 2022. The majority of patients presented with early-stage disease, with stage I accounting for 24.7% (*n* = 20), stage II accounting for 59.3% (*n* = 48), stage III accounting for 9.9% (*n* = 8), and stage IV accounting for 6.2% (*n* = 5) of cases (Table 1). Initial clinical lymph node assessment identified 72.8% (*n* = 59) of patients as node-negative (cN0) and 27.2% (*n* = 22) as node-positive (cN+) prior to treatment initiation.

Molecular subtyping, defined according to hormone receptor (HR) status, HER2 expression, and Ki-67 proliferation index, revealed diverse breast cancer phenotypes. Luminal A (HR+/HER2− with Ki-67 <14%) was identified in 19.8% (*n* = 16) of patients, luminal B (HR+/HER2+ with Ki-67 ≥14%) in 34.6% (*n* = 28), luminal HER2 in 14.8% (*n* = 12), HER2-enriched in 13.6% (*n* = 11), and triple-negative breast cancer in 17.3% (*n* = 14) of the cohort.

Regarding treatment modality, 32.1% (*n* = 26) of patients received neoadjuvant chemotherapy, 61.7% (*n* = 50) received adjuvant chemotherapy, and 6.2% (*n* = 5) received palliative chemotherapy. Among patients who underwent neoadjuvant chemotherapy, pathologic complete response (pCR) was achieved in 26.9% (*n* = 7), while 73.1% (*n* = 19) did not attain pCR. Hormone therapy was administered to 69.1% (*n* = 56) of patients. At follow-up, 86.4% (*n* = 70) of patients remained disease-free, while 7.4% (*n* = 6) experienced recurrence and 6.2% (*n* = 5) had stage IV disease.

### 3.2. Chemotherapy-Related Adverse Event Profile

The spectrum of chemotherapy-related adverse events observed in this cohort is detailed in Table 2. Hematologic toxicities predominated, with neutropenia representing the most common adverse event, affecting 96.3% of patients at any grade and 81.5% at grade 3 or higher. Anemia occurred in 64.2% of patients, though severe cases (grade ≥ 3) were less frequent at 8.6%. Thrombocytopenia was documented in 25.9% of patients, with all cases classified as grade 1–2 severity.

Non-hematologic toxicities were also common, with peripheral neuropathy emerging as the most prevalent (50.6%), including 6.2% of cases at grade 3 or higher. Hepatotoxicity, manifested as AST elevation, affected 48.1% of patients, predominantly at lower grades (grade ≥ 3: 2.5%). Gastrointestinal adverse events included diarrhea (39.5%), nausea (28.4%), and oral mucositis (16.0%), all predominantly mild to moderate in severity. Fatigue was reported in 38.3% of patients. Dermatologic manifestations, including skin rash (22.2%) and hand-foot syndrome (13.6%), were less common. Notably, aside from neutropenia, anemia, and peripheral neuropathy, severe adverse events (grade ≥ 3) were infrequent, with most toxicities remaining manageable at lower grades.

### 3.3. Gut Microbiota Alterations Associated with Chemotherapy-Induced Hematologic Toxicity

#### 3.3.1. Anemia

Baseline fecal samples from 81 treatment-naïve patients were analyzed. Comparative microbiome analysis between patients with mild anemia (grades 0–1, *n* = 60) and severe anemia (grades 2–3, *n* = 21) revealed significant compositional alterations despite comparable microbial richness. Alpha diversity assessment using the Chao1 estimator demonstrated no significant differences between groups across all taxonomic levels (family *p* = 0.249, genus *p* = 0.150, species *p* = 0.241) (Supplementary Figure S1a). However, beta diversity analysis using PERMANOVA revealed significant community structure differences at the family (F = 2.50, R^2^ = 3.07%, *p* = 0.034) and genus (F = 2.20, R^2^ = 2.71%, *p* = 0.027) levels, with principal coordinate axes accounting for 41.1–58.4% of total variance (Figure S1b). Linear discriminant analysis effect size (LEfSe) identified multiple differentially abundant bacterial taxa with robust statistical and biological significance (Table 3 and Figure S1c). At the family level, Eubacteriaceae exhibited substantial enrichment in severe anemia with a large effect size (Cohen’s d = 1.380, 95% CI [0.840, 1.921], FDR q = 0.0053, LDA = 2.89), as did Enterobacteriaceae (Cohen’s d = 0.601, 95% CI [0.096, 1.106], FDR q = 0.013, LDA = 4.37). Peptostreptococcaceae showed smaller but significant effects (Cohen’s d = 0.192, FDR q = 0.015). At the genus level, Anaerofilum demonstrated the strongest association with severe anemia (Cohen’s d = 1.423, 95% CI [0.880, 1.966], FDR q = 0.0053, LDA = 2.12), followed by Eubacterium (Cohen’s d = 1.380, 95% CI [0.840, 1.921], FDR q = 0.0053, LDA = 2.89) and Eubacterium_brachy_group (Cohen’s d = 1.066, 95% CI [0.542, 1.589], FDR q = 0.0086, LDA = 2.37). Intestinibacter and Erysipelatoclostridium were also enriched, though with negligible effect sizes (Cohen’s d < 0.2, FDR q = 0.0053). Species-level analysis revealed large effect sizes for Eubacterium limosum (Cohen’s d = 1.380, 95% CI [0.840, 1.921], FDR q = 0.0053, LDA = 2.89) and Massiliomicrobiota timonensis (Cohen’s d = 1.400, 95% CI [0.858, 1.942], FDR q = 0.023, LDA = 2.01), along with a medium effect size for Clostridium scindens (Cohen’s d = 0.633, 95% CI [0.126, 1.139], FDR q = 0.037, LDA = 1.70).

#### 3.3.2. Neutropenia

Analysis comparing patients with mild neutropenia (grades 0–3, *n* = 29) to those with severe neutropenia (grade 4, *n* = 52) revealed distinct compositional patterns. Alpha diversity showed consistent but non-significant trends toward reduced microbial richness in severe neutropenia across all taxonomic levels (family *p* = 0.210, genus *p* = 0.160, species *p* = 0.170) (Figure S2a). Beta diversity analysis demonstrated progressive differentiation in community structure from family level (F = 0.966, *p* = 0.415) to species level (F = 1.812, *p* = 0.089), with species-level composition approaching significance and explaining 2.2% of variance between groups (Figure S2b). LEfSe analysis identified significant biomarkers with robust effect sizes (Table 3 and Figure S2c). At the family level, Coriobacteriales_Incertae_Sedis showed statistical but not biological significance (Cohen’s d = −0.157, 95% CI [−0.612, 0.298], FDR q = 0.0035, LDA = −1.75). At the genus level, Eubacterium_hallii_group exhibited large effect size depletion in severe neutropenia (Cohen’s d = −1.081, 95% CI [−1.565, −0.598], FDR q = 0.0089, LDA = −3.91), while Fournierella showed small effect size enrichment (Cohen’s d = 0.231, 95% CI [−0.225, 0.687], FDR q = 0.0089, LDA = 1.52). At the species level, Intestinimonas_butyriciproducens exhibited large effect size depletion (Cohen’s d = −1.115, 95% CI [−1.601, −0.630], FDR q = 0.0089, LDA = −2.44). These findings suggest that butyrate-producing bacteria, particularly Eubacterium_hallii_group, are significantly depleted in severe neutropenia, indicating that altered short-chain fatty acid metabolism may contribute to the severity of chemotherapy-induced immunosuppression.

#### 3.3.3. Thrombocytopenia

Comparison between patients with thrombocytopenia (grades 1–2, *n* = 21) and those without thrombocytopenia (grade 0, *n* = 60) revealed minimal community-level differences. Alpha diversity analysis showed no significant differences across all taxonomic levels (*p* > 0.05) (Figure S3a), though positive trends toward increased richness in thrombocytopenia patients strengthened progressively from family (*p* = 0.365) to species level (*p* = 0.129). Beta diversity analysis demonstrated subtle, non-significant compositional differences, with effect sizes increasing from family (R^2^ = 0.012, *p* = 0.433) to species level (R^2^ = 0.016, *p* = 0.226) (Figure S3b). LEfSe analysis identified Eubacterium genus and Eubacterium limosum species as the sole significant biomarkers with large effect sizes, showing dramatic depletion in thrombocytopenia patients (mean abundance: 39.1 vs. 2055; Cohen’s d = −1.604, 95% CI [−2.159, −1.049], FDR q = 0.0083, LDA = −3.0), representing an approximately 53-fold reduction compared to non-thrombocytopenia patients (Table 3 and Figure S3c).

### 3.4. Gut Microbiota Alterations Associated with Chemotherapy-Induced Hepatotoxicity

#### 3.4.1. ALT (GPT) Elevation

Patients with ALT elevation (grades 1–3, *n* = 39) demonstrated significant alterations across multiple diversity metrics compared to those without elevation (grade 0, *n* = 42). Alpha diversity analysis revealed significantly reduced microbial richness at family (*p* = 0.0048), genus (*p* = 0.013), and species (*p* = 0.016) levels (Figure S4a). Beta diversity assessment confirmed significant community structure differences at family (F = 2.8538, *p* = 0.031, R^2^ = 0.035) and genus (F = 2.6372, *p* = 0.020, R^2^ = 0.032) levels, though significance was lost at the species level (*p* = 0.202) (Figure S4b). LEfSe analysis identified specific biomarkers depleted in patients with hepatotoxicity, all demonstrating statistical and biological significance after FDR correction (Table 3 and Figure S4c). At the genus level, Eubacterium_hallii_group exhibited large effect size depletion (Cohen’s d = −0.994, 95% CI [−1.456, −0.532], FDR q = 0.0045, LDA = −3.77), and Burkholderia_Caballeronia_Paraburkholderia showed similarly large depletion (Cohen’s d = −1.191, 95% CI [−1.664, −0.718], FDR q = 0.012, LDA = −2.64). At the family level, Burkholderiaceae (Cohen’s d = −0.679, 95% CI [−1.127, −0.231], FDR q = 0.012, LDA = −3.65) and Acidaminococcaceae (Cohen’s d = −0.561, 95% CI [−1.006, −0.117], FDR q = 0.012, LDA = −3.92) demonstrated medium effect size depletion, while Marinifilaceae showed statistical but minimal biological significance (Cohen’s d = −0.177, FDR q = 0.012, LDA = −2.89). These findings suggest that ALT elevation is associated with loss of potentially protective bacterial taxa, particularly butyrate-producing species, rather than pathogenic bacterial overgrowth.

#### 3.4.2. AST (GOT) Elevation

AST elevation (grades 1–3, *n* = 22) was associated with distinct microbial signatures compared to no elevation (grade 0, *n* = 59). Alpha diversity demonstrated statistically significant differences specifically at the genus level (*p* = 0.04958), while family and species levels showed non-significant trends (Figure S5a). Beta diversity profiling revealed significant compositional differences across all taxonomic levels, with the strongest separation at the species level (F = 2.9475, R^2^ = 0.036, *p* = 0.012), followed by family (*p* = 0.023) and genus (*p* = 0.027) levels (Figure S5b). LEfSe analysis identified key microbial biomarkers with robust statistical and biological significance (Table 3 and Figure S5c). Akkermansiaceae and Akkermansia showed large effect size depletion in AST-elevated patients (Cohen’s d = −0.962, 95% CI [−1.473, −0.450], FDR q = 0.0085, LDA = −4.03), while Bacteroidaceae demonstrated small effect size depletion (Cohen’s d = −0.280, FDR q = 0.032, LDA = −4.85). Conversely, genera associated with Ruminococcaceae were notably enriched: Subdoligranulum exhibited large effect size enrichment (Cohen’s d = 1.054, 95% CI [0.538, 1.570], FDR q = 0.011, LDA = 3.82), Butyricicoccus showed medium effect size enrichment (Cohen’s d = 0.783, 95% CI [0.278, 1.287], FDR q = 0.0085, LDA = 3.02), and the species metagenome demonstrated large effect size enrichment (Cohen’s d = 1.319, 95% CI [0.789, 1.849], FDR q = 0.012, LDA = 3.52). The Ruminococcaceae family showed small effect size enrichment overall (Cohen’s d = 0.494, 95% CI [−0.002, 0.989], FDR q = 0.014, LDA = 4.58).

### 3.5. Gut Microbiota Alterations Associated with Chemotherapy-Induced Gastrointestinal Toxicity

#### 3.5.1. Nausea

Patients with nausea (grades 1–2, *n* = 23) showed no significant community-level differences compared to those without nausea (grade 0, *n* = 58). Alpha diversity analysis revealed no significant differences at family (*p* = 0.557), genus (*p* = 0.567), or species (*p* = 0.524) level (Figure S6a). Similarly, beta diversity analysis showed no significant compositional differences at any taxonomic level (family: *p* = 0.994, R^2^ = 0.0018; genus: *p* = 0.992, R^2^ = 0.0042; species: *p* = 0.355, R^2^ = 0.013) (Figure S6b). Despite the absence of overall community-level differences, LEfSe analysis identified potential species-level biomarkers with varying levels of significance (Table 3 and Figure S6c). Intestinimonas butyriciproducens showed large effect size enrichment in patients without nausea (Cohen’s d = 1.541, 95% CI [1.002, 2.079], FDR q = 0.033, LDA = 2.34), achieving both statistical and biological significance. Two additional butyrate-producing bacteria demonstrated large effect sizes but did not survive FDR correction: Christensenella_minuta (Cohen’s d = 1.667, 95% CI [1.120, 2.214], raw *p* = 0.035, FDR q = 0.061, LDA = 1.51) and Faecalibacterium_prausnitzii (Cohen’s d = 1.301, 95% CI [0.779, 1.824], raw *p* = 0.046, FDR q = 0.061, LDA = 2.76). Bacteroides_coprocola_DSM_17136 showed medium effect size enrichment in nausea patients but was not statistically significant (Cohen’s d = −0.740, 95% CI [−1.236, −0.244], raw *p* = 0.082, FDR q = 0.082, LDA = −4.35). These findings suggest that chemotherapy-induced nausea may be associated with depletion of specific butyrate-producing taxa rather than broad alterations in overall microbiome composition.

#### 3.5.2. Oral Mucositis

Patients who developed oral mucositis (grades 1–2, *n* = 13) demonstrated no significant differences in microbial richness compared to those without this complication (grade 0, *n* = 68) at any taxonomic level (family *p* = 0.267, genus *p* = 0.231, species *p* = 0.266) (Figure S7a). Beta diversity analysis similarly demonstrated no significant differences in overall community composition (family: R^2^ = 0.007, *p* = 0.717; genus: R^2^ = 0.014, *p* = 0.348; species: R^2^ = 0.010, *p* = 0.581) (Figure S7b), indicating that mucositis status explained minimal variance in microbiome structure. LEfSe analysis identified three biomarker candidates with statistical and biological significance after FDR correction (Table 3 and Figure S7c). At the genus level, Coprococcus_3 showed large effect size depletion in mucositis patients (Cohen’s d = 1.134, 95% CI [0.516, 1.753], FDR q = 0.025, LDA = 3.14), and Ruminococcaceae_UCG_014 demonstrated large effect size depletion (Cohen’s d = 1.667, 95% CI [1.020, 2.313], FDR q = 0.027, LDA = 3.51). At the species level, gut_metagenome exhibited medium effect size depletion in mucositis patients (Cohen’s d = 0.762, 95% CI [0.157, 1.367], FDR q = 0.025, LDA = 4.02). All three taxa were enriched in non-mucositis patients, suggesting protective effects of these commensal bacteria against chemotherapy-induced oral mucosal damage.

#### 3.5.3. Diarrhea

Analysis of patients with diarrhea (grades 1–2, *n* = 32) revealed no significant differences in alpha diversity (family *p* = 0.690, genus *p* = 0.804, species *p* = 0.704) (Figure S8a) or beta diversity (family: F = 1.35, *p* = 0.186; genus: F = 1.17, *p* = 0.249; species: F = 1.18, *p* = 0.272) (Figure S8b) compared to those without diarrhea (grade 0, n = 49), with diarrhea status explaining less than 2% of compositional variance. LEfSe analysis identified Christensenella (genus) and Christensenella_minuta (species) as significantly depleted in the diarrhea group with both statistical and biological significance (Cohen’s d = 1.509, 95% CI [1.007, 2.011], FDR q = 0.041, LDA = 1.55), indicating that depletion of this specific taxon, rather than widespread microbial alterations, may be associated with chemotherapy-induced diarrhea pathogenesis (Table 3 and Supplementary Figure S8c).

### 3.6. Gut Microbiota Alterations Associated with Chemotherapy-Induced Neurological Toxicity

Peripheral sensory neuropathy (PSN; grades 1–2, *n* = 41) showed no significant associations with microbial diversity patterns compared to patients without PSN (grade 0, *n* = 40). Alpha diversity profiling demonstrated comparable microbial richness between groups across all taxonomic levels (family *p* = 0.940, genus *p* = 0.513, species *p* = 0.408) (Figure S9a). Beta diversity analysis similarly showed no significant compositional differences at any level (family: F = 0.868, *p* = 0.506, R^2^ = 1.10%; genus: F = 1.152, *p* = 0.301, R^2^ = 1.45%; species: F = 0.913, *p* = 0.507, R^2^ = 1.16%) Figure S9b). LEfSe analysis identified Clostridium scindens as nominally enriched in the non-PSN group (mean abundance: 122.67 vs. 51.06, raw *p* = 0.024, LDA = 1.57); however, this difference did not remain significant after FDR correction (FDR-adjusted *p* = 0.581) (Table 3 and Figure S9c), suggesting no robust microbiome associations with chemotherapy-induced peripheral neuropathy in this cohort.

### 3.7. Gut Microbiota Alterations Associated with Chemotherapy-Induced Dermatologic Toxicity

#### 3.7.1. Maculopapular Rash

Patients who developed skin rash (grades 1–3, *n* = 18) exhibited no significant differences in microbial diversity compared to those without rash (grade 0, *n* = 63). Alpha diversity showed comparable microbial richness at all taxonomic levels (family *p* = 0.802, genus *p* = 0.816, species *p* = 0.845) (Figure S10a). Beta diversity assessment similarly revealed no significant compositional differences (family: F = 0.797, *p* = 0.517; genus: F = 0.991, *p* = 0.386; species: F = 1.351, *p* = 0.182) (Figure S10b), with skin rash status explaining less than 2% of community variation. LEfSe analysis identified Bacteroides caccae as potentially discriminative (*p* = 0.012, LDA = 2.12), showing higher abundance in the no-rash group; however, this finding became non-significant after FDR correction (adjusted *p* = 0.584) (Table 3 and Figure S10c). These results suggest that chemotherapy-induced maculopapular rash is not associated with broad taxonomic shifts in gut microbiota composition.

#### 3.7.2. Hand-Foot Syndrome

In contrast to other dermatologic toxicities, hand-foot syndrome (HFS; grades 1–2, *n* = 11) showed significant associations with microbial profiles compared to patients without HFS (grade 0, *n* = 70). Alpha diversity demonstrated significantly higher microbial richness in HFS patients across all taxonomic levels (family: t = −3.25, *p* = 0.0027; genus: t = −3.21, *p* = 0.0041; species: t = −2.75, *p* = 0.013) (Figure S11a). Beta diversity analysis showed marginally significant differences in microbial community composition at the family level (F = 2.251, *p* = 0.044, FDR = 0.051), but not at genus or species levels (Figure S11b). LEfSe analysis identified multiple genera as robust biomarkers with statistical and biological significance after FDR correction (Table 3 and Figure S11c). Two butyrate-producing genera from Lachnospiraceae showed large effect size depletion in HFS patients: Butyricicoccus (Cohen’s d = −1.084, 95% CI [−1.741, −0.426], FDR q = 0.0013, LDA = −3.5) and Roseburia (Cohen’s d = −0.959, 95% CI [−1.612, −0.306], FDR q = 0.0013, LDA = −3.88). At the family level, Eggerthellaceae exhibited large effect size depletion (Cohen’s d = −1.087, 95% CI [−1.745, −0.430], FDR q = 0.0042, LDA = −3.12), while Lachnospiraceae showed small effect size depletion overall (Cohen’s d = −0.408, 95% CI [−1.047, 0.231], FDR q = 0.0050, LDA = −4.61). These findings suggest that depletion of butyrate-producing bacterial taxa combined with elevated gut microbiome diversity may be associated with chemotherapy-induced palmar-plantar erythrodysesthesia syndrome.

### 3.8. Gut Microbiota Alterations Associated with Chemotherapy-Induced Fatigue

Fatigue (grades 1–2, *n* = 31) showed no statistically significant associations with microbial diversity metrics compared to patients without fatigue (grade 0, *n* = 50). Alpha diversity demonstrated comparable microbial richness between groups at all taxonomic levels (family *p* = 0.607, genus *p* = 0.621, species *p* = 0.619) (Figure S12a). Beta diversity analysis similarly showed no significant differences in microbial community composition across all levels, with fatigue status accounting for minimal variance (R^2^ = 0.51% at family level, 0.54% at genus level, 1.18% at species level; all *p* > 0.05) (Figure S12b). LEfSe analysis identified only Bacteroides_vulgatus with small effect size enrichment in the fatigue group (Cohen’s d = −0.496, 95% CI [−0.950, −0.041], FDR q = 0.026, LDA = −4.22), achieving statistical but minimal biological significance (Table 3 and Figure S12c). Despite the presence of diverse bacterial taxa including Bacteroidaceae, Lachnospiraceae, and various Bacteroides species in patients with fatigue, the overall results indicate that chemotherapy-induced fatigue was not associated with substantial alterations in gut microbiome diversity or composition in this breast cancer patient cohort.

### 3.9. Summary of Bacterial Family Associations Across Adverse Events

**Figure 1:**
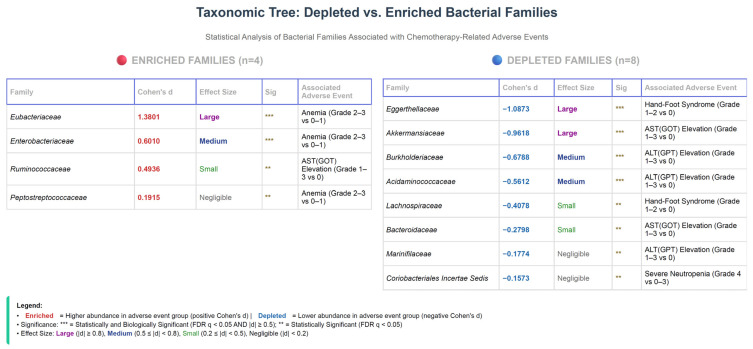
Differential abundance of bacterial families associated with chemotherapy-related adverse events. Taxonomic analysis revealed 12 bacterial families with statistically significant differential abundance between patients with and without specific adverse events. Families are categorized as enriched (red, *n* = 4; higher relative abundance in adverse event groups, positive Cohen’s d values) or depleted (blue, *n* = 8; lower relative abundance in adverse event groups, negative Cohen’s d values). Each family is annotated with Cohen’s d effect size, magnitude classification, statistical significance level, and associated adverse event comparison. Enriched families include *Eubacteriaceae* (largest effect, Cohen’s d = 1.38) and *Enterobacteriaceae* (d = 0.60), both associated with anemia. Depleted families include *Eggerthellaceae* (d = −1.09) and *Lachnospiraceae* (d = −0.41) in hand-foot syndrome, *Akkermansiaceae* (d = −0.96) in AST elevation, and multiple families (*Burkholderiaceae*, *Acidaminococcaceae*, *Marinifilaceae*) in hepatotoxicity. Statistical significance is denoted as: *** = statistically and biologically significant (FDR q-value < 0.05 AND |Cohen’s d| ≥ 0.5); ** = statistically significant (FDR q-value < 0.05 but |Cohen’s d| < 0.5). Effect sizes are classified according to Cohen’s guidelines: Large (|d| ≥ 0.8), Medium (0.5 ≤ |d| < 0.8), Small (0.2 ≤ |d| < 0.5), and Negligible (|d| < 0.2). All *p*-values were adjusted for multiple testing using the Benjamini–Hochberg false discovery rate (FDR) correction.

Taxonomic analysis of gut microbiome composition revealed significant associations between bacterial family abundance and chemotherapy-related adverse events in breast cancer patients (Figure 1). Twelve bacterial families demonstrated statistically significant differential abundance patterns across multiple adverse event comparisons. Among enriched families (higher abundance in adverse event groups), Eubacteriaceae showed the strongest association with anemia (Cohen’s d = 1.38, FDR q < 0.01), followed by Enterobacteriaceae (Cohen’s d = 0.60, FDR q = 0.013). Conversely, eight families were significantly depleted in adverse event groups. Eggerthellaceae showed the largest depletion effect in hand-foot syndrome patients (Cohen’s d = −1.09, FDR q = 0.004), and Akkermansiaceae demonstrated substantial depletion in patients with AST elevation (Cohen’s d = −0.96, FDR q = 0.009). Additional depleted families associated with hepatotoxicity included Burkholderiaceae, Acidaminococcaceae, and Marinifilaceae in ALT elevation cases, and Bacteroidaceae in AST elevation cases. Notably, Lachnospiraceae depletion was observed in hand-foot syndrome patients, while Coriobacteriales Incertae Sedis showed depletion in severe neutropenia cases. These findings suggest that specific bacterial families may serve as potential biomarkers for predicting chemotherapy-related toxicity, with both enrichment and depletion patterns demonstrating clinical relevance across hematologic, hepatic, and dermatologic adverse events.

### 3.10. Directional Association Patterns Between Gut Microbiota and Adverse Events

**Figure 2:**
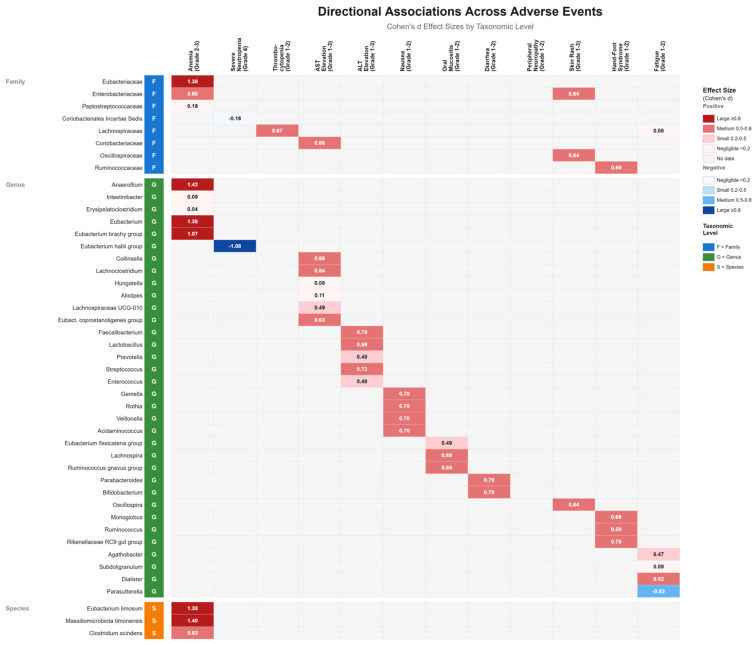
Heatmap of Directional Associations Between Gut Microbiota and Chemotherapy-Related Adverse Events. Comprehensive visualization of Cohen’s d effect sizes showing directional associations between gut microbial taxa and adverse events across multiple taxonomic levels. Rows represent 46 bacterial taxa organized hierarchically by taxonomic classification: Family (F, blue labels), Genus (G, green labels), and Species (S, orange labels). Columns represent 12 adverse event categories grouped by clinical domain: hematologic toxicities (anemia, severe neutropenia, thrombocytopenia), hepatic toxicities (AST and ALT elevation), and non-hematologic toxicities (nausea, oral mucositis, diarrhea, peripheral neuropathy, skin rash, hand-foot syndrome, and fatigue). Cell colors indicate both the direction and magnitude of associations: red gradients represent positive associations (taxa enriched in adverse event groups), with dark red indicating large effect sizes (|Cohen’s d| ≥ 0.8), medium red for medium effects (0.5 ≤ |d| < 0.8), and light red for small effects (0.2 ≤ |d| < 0.5); blue gradients represent negative associations (taxa depleted in adverse event groups) with corresponding intensity scales; gray cells indicate no significant association. Effect sizes were calculated using Cohen’s d with 95% confidence intervals, and statistical significance was determined using Mann–Whitney U tests with Benjamini–Hochberg false discovery rate (FDR) correction (q < 0.05). Only taxa demonstrating statistical significance (FDR q < 0.05 or nominal *p* < 0.05 with |Cohen’s d| ≥ 0.5) are displayed. Numerical values within cells represent Cohen’s d coefficients rounded to two decimal places. The heatmap reveals predominantly positive associations (43 of 46 significant relationships), with the strongest effects observed for Eubacteriaceae family members in anemia and notable negative associations for Eubacterium hallii group in severe neutropenia and Parasutterella in fatigue. Sample sizes varied by adverse event comparison as indicated in Table 3.

**Figure 3:**
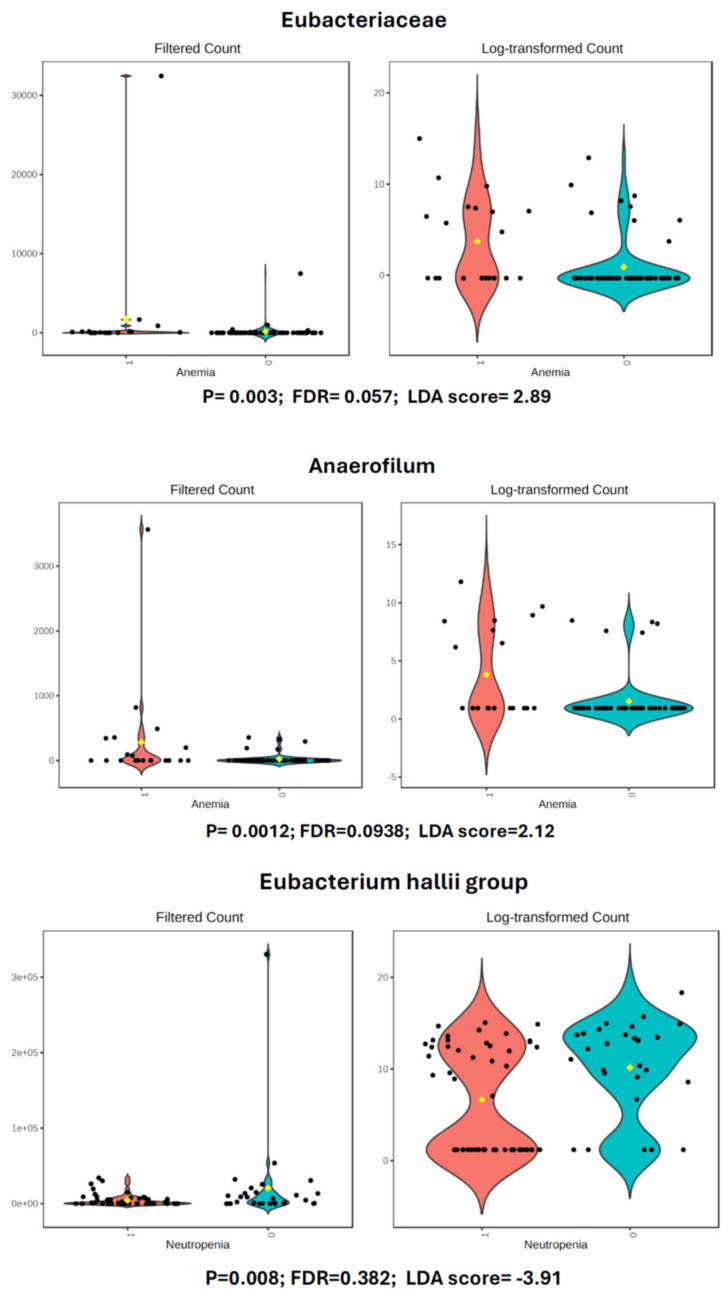

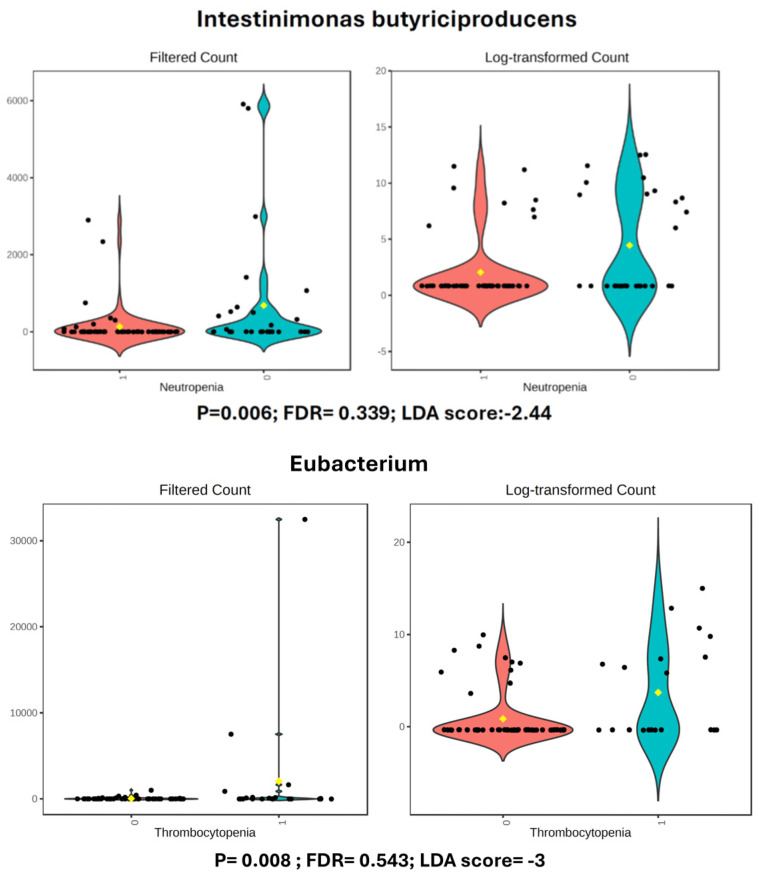
Differential abundance of Eubacteriaceae family members associated with chemotherapy-induced hematological toxicities. Violin plots display the abundance distribution of selected bacterial taxa from the Eubacteriaceae family and related genera significantly associated with hematological adverse events. Each panel presents filtered counts (**left**) and log-transformed counts (**right**) for comparison between adverse event groups (red) and control groups without the respective toxicity (teal). Black dots represent individual samples, and horizontal black lines indicate median values. Statistical significance was determined using linear discriminant analysis effect size (LEfSe), with *p*-values, false discovery rate (FDR)-adjusted *p*-values, and LDA scores provided for each comparison. The Eubacteriaceae family and Anaerofilum genus showed significant enrichment in patients with anemia (*p* = 0.003, FDR = 0.057, LDA score = 2.89; *p* = 0.0012, FDR = 0.0938, LDA score = 2.12, respectively), representing the largest effect sizes observed in the hematological toxicity analyses (Cohen’s d = 1.38 and d = 1.42, respectively). Conversely, the Eubacterium hallii group demonstrated significant depletion in patients with severe (grade 4) neutropenia (*p* = 0.008, FDR = 0.382, LDA score = −3.91; Cohen’s d = −1.08), while Intestinimonas butyriciproducens and Eubacterium genus also showed differential abundance patterns associated with neutropenia and thrombocytopenia, respectively (*p* = 0.006, FDR = 0.339, LDA score = −2.44; *p* = 0.008, FDR = 0.543, LDA score = −3). These findings highlight distinct microbiome signatures associated with impaired hematopoiesis during chemotherapy treatment.

Heatmap analysis revealed distinct directional patterns of microbiota-adverse event associations spanning multiple taxonomic levels (Figure 2). Anemia (grades 2–3) demonstrated the strongest and most numerous associations, with large positive effect sizes observed for Eubacteriaceae family (d = 1.38) and multiple genera including Anaerofilum (d = 1.42), Eubacterium (d = 1.38), and Eubacterium brachy group (d = 1.07), as well as species Massiliomicrobiota timonensis (d = 1.40) and Eubacterium limosum (d = 1.38), indicating substantial enrichment of these taxa in anemic patients. The largest effect observed in our dataset was the enrichment of the Eubacteriaceae family in anemia (d = 1.38), accompanied by substantial enrichment of Anaerofilum (d = 1.42), Massiliomicrobiota timonensis (d = 1.40), and the pro-inflammatory family Enterobacteriaceae (d = 0.60) (Figure 3). Conversely, severe neutropenia (grade 4) exhibited a notable negative association with Eubacterium hallii group (d = −1.08), suggesting depletion of this genus in severely neutropenic patients. These distinct microbiome signatures associated with chemotherapy-induced hematological toxicities provide evidence for a bidirectional microbiome-hematopoiesis axis that may influence blood cell production during cancer treatment. Hepatic enzyme elevations showed consistent medium-effect associations, with AST elevation linked to Coriobacteriaceae family and Collinsella genus (both d = 0.66), while ALT elevation associated with Faecalibacterium (d = 0.70) and Streptococcus (d = 0.72). Gastrointestinal adverse events demonstrated moderate positive associations, including nausea with multiple genera (Gemella, Rothia, Veillonella, Acidaminococcus; all d = 0.70) and oral mucositis with Lachnospira (d = 0.69). Fatigue exhibited mixed directionality, with positive associations for Agathobacter (d = 0.47) and Dialister (d = 0.52), but a negative association with Parasutterella (d = −0.53). The majority of significant associations were positive (enrichment in adverse event groups), with only three taxa showing negative associations, suggesting that chemotherapy-related adverse events are predominantly characterized by expansion rather than depletion of specific microbial taxa.

### 3.11. Effect Size Distribution and Biomarker Potential

**Figure 4:**
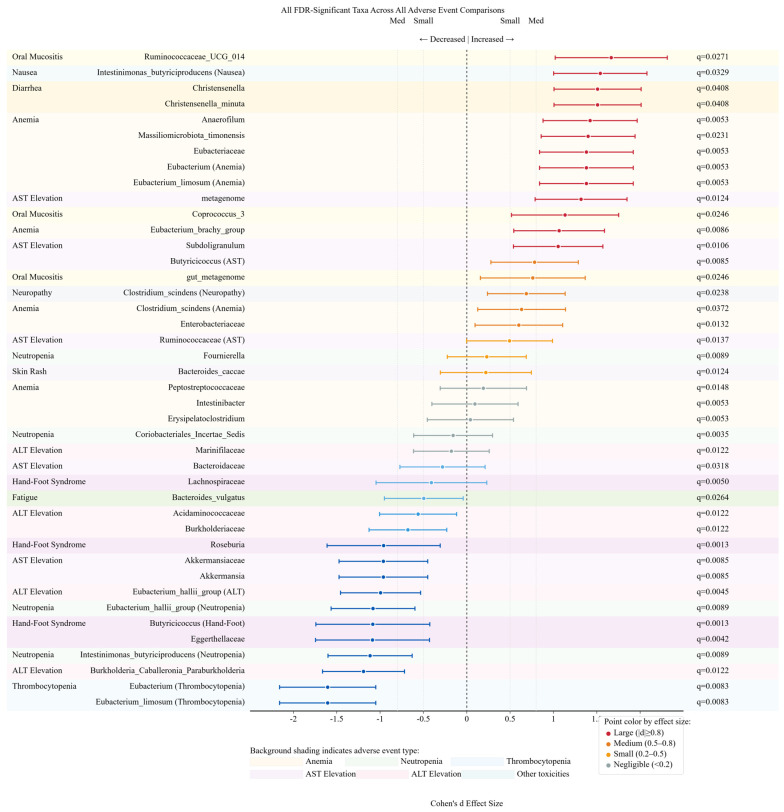
Effect sizes and confidence intervals of all FDR-significant bacterial taxa associated with chemotherapy-induced adverse events. Forest plot displaying Cohen’s d effect sizes with 95% confidence intervals for 42 bacterial taxa demonstrating statistically significant associations (FDR q < 0.05) across all adverse event comparisons. Each horizontal line represents the 95% confidence interval for the effect size estimate, with the point estimate indicated by a dot. Effect sizes are color-coded by magnitude: large (|d| ≥ 0.8, red), medium (0.5 ≤ |d| < 0.8, orange), small (0.2 ≤ |d| < 0.5, yellow), and negligible (<0.2, blue), with the vertical dashed line at zero indicating no effect. Positive effect sizes (right of zero) indicate bacterial enrichment in adverse event groups, while negative effect sizes (left of zero) indicate depletion. The adverse event associated with each taxon is listed on the left, with FDR-adjusted q-values provided on the right. Effect sizes ranged from d = −1.60 (Eubacterium and Eubacterium_limosum in thrombocytopenia) to d = 1.67 (Ruminococcaceae_UCG_014 in oral mucositis), with 23 taxa (54.8%) demonstrating medium to large effect sizes (|d| ≥ 0.5), indicating biologically meaningful associations. Anemia exhibited the most extensive microbiome alterations with 11 significant taxa associations, followed by AST elevation (n = 7) and hand-foot syndrome (n = 4), highlighting adverse event-specific microbiome signatures with potential biomarker utility for predicting chemotherapy toxicity.

Forest plot analysis revealed 42 taxa with statistically significant associations (FDR q < 0.05) across all adverse event comparisons, with effect sizes ranging from Cohen’s d = −1.60 to +1.67 (Figure 4). The largest positive effect sizes were observed for Ruminococcaceae_UCG_014 in oral mucositis (d = 1.67, 95% CI: 1.02–2.31, q = 0.027), Eubacterium (d = −1.60, 95% CI: −2.16 to −1.05, q = 0.008) and Eubacterium_limosum (d = −1.60, 95% CI: −2.16 to −1.05, q = 0.008) in thrombocytopenia, and Intestinimonas_butyriciproducens in nausea (d = 1.54, 95% CI: 1.00–2.08, q = 0.033). Notable negative associations included Eubacterium_hallii_group in both severe neutropenia (d = −1.08, 95% CI: −1.57 to −0.60, q = 0.009) and ALT elevation (d = −0.99, 95% CI: −1.46 to −0.53, q = 0.005), as well as Burkholderia_Caballeronia_Paraburkholderia in ALT elevation (d = −1.19, 95% CI: −1.66 to −0.72, q = 0.012). Among the 42 significant taxa, 23 (54.8%) demonstrated medium to large effect sizes (|d| ≥ 0.5), indicating biologically meaningful associations beyond statistical significance. Anemia showed the highest number of significant taxa associations (n = 11), followed by AST elevation (n = 7) and hand-foot syndrome (n = 4), suggesting adverse event-specific microbiome signatures that may serve as potential predictive biomarkers for chemotherapy toxicity.

## 4. Discussion

While our study identifies associations between baseline microbiota and subsequent chemotherapy toxicity, the cross-sectional design precludes causal inference, and these findings represent predictive associations rather than proven causal relationships. Our analysis identified multiple bacterial taxa at baseline associated with chemotherapy-related adverse events that developed during treatment, with FDR q < 0.05 and effect sizes ranging from substantial depletion (Cohen’s d = −1.60) to marked enrichment (Cohen’s d = 1.67), suggesting potential predictive value for identifying patients at risk for specific toxicities. The microbiome signature patterns predicting toxicity risk are toxicity-specific rather than uniform, characterized by both baseline depletion of protective commensal bacteria and baseline enrichment of potentially pathogenic taxa that may be associated with subsequent adverse events.

A striking finding in our study is the paradoxical baseline associations between butyrate-producing bacteria and subsequent chemotherapy toxicities, which challenge the conventional paradigm that beneficial bacteria universally confer protective effects. This association does not establish causation, and multiple alternative explanations merit consideration. While we observed expected baseline depletions of butyrate producers—Eubacterium hallii group in patients who developed neutropenia (d = −1.08), Roseburia in those experiencing hand-foot syndrome (d = −0.96), and Lachnospiraceae in epithelial toxicities (d = −0.41)—consistent with their established roles in maintaining barrier integrity, stimulating regulatory T cells, and suppressing inflammation through histone deacetylase inhibition [29,30], we paradoxically identified significant baseline enrichment of other butyrate producers in patients who subsequently developed adverse events: Ruminococcaceae_UCG_014 showed the largest positive effect in oral mucositis (d = 1.67), while Faecalibacterium predicted ALT elevation (d = 0.70). These contradictory findings likely reflect several mechanisms: baseline enrichment may indicate pre-existing vulnerability or represent subclinical inflammatory states that predispose to toxicity rather than causative factors; butyrate’s biological effects can be context-dependent based on local concentration, tissue type, and inflammatory milieu; and strain-level functional heterogeneity beyond butyrate production determines net physiological outcomes [31,32]. The bidirectional associations observed with Intestinimonas butyriciproducens—enriched at baseline in patients who developed nausea (d = 1.54) but depleted in those experiencing neutropenia (d = −1.12)—further underscore this complexity and suggest that precision microbiome interventions tailored to specific toxicity types, rather than universal butyrate producer supplementation, will be required for effective chemotherapy toxicity prediction and prevention.

The distinct baseline microbiome signatures associated with chemotherapy-induced hematological toxicities provide evidence for potential bidirectional microbiome-hematopoiesis relationships that may be associated with blood cell production during cancer treatment. These associations do not prove causation, and experimental validation is required to establish mechanistic relationships. The largest effect observed in our dataset was the baseline enrichment of the Eubacteriaceae family in patients who developed anemia (d = 1.38), accompanied by substantial enrichment of Anaerofilum (d = 1.42), Massiliomicrobiota timonensis (d = 1.40), and the pro-inflammatory family Enterobacteriaceae (d = 0.60). These enriched taxa are primarily pro-inflammatory organisms whose baseline presence may predispose to cytokine-mediated anemia through multiple interconnected mechanisms: pro-inflammatory cytokines such as IL-6, IL-1β, and TNF-α suppress erythropoiesis both by reducing erythropoietin production and by directly interfering with erythroid progenitor differentiation, while simultaneously inducing hepcidin expression that causes iron sequestration in macrophages and enterocytes, rendering iron unavailable for hemoglobin synthesis despite adequate stores [33,34,35]. Conversely, neutropenia showed strong associations with baseline depletion of beneficial commensals—Eubacterium hallii group (d = −1.08) and Intestinimonas butyriciproducens (d = −1.12)—while thrombocytopenia exhibited severe baseline depletion of Eubacterium species (d = −1.60), the strongest negative association in our study. Emerging evidence demonstrates that commensal gut bacteria sustain steady-state hematopoiesis through microbial metabolites that enter circulation and trigger cytokine signaling affecting hematopoietic stem and progenitor cells; antibiotic-induced microbiota depletion causes neutropenia, anemia, and pan-lymphopenia that are reversed by fecal microbiota transplantation, establishing causality for this relationship [36,37]. The bidirectional nature of Eubacterium associations—depleted at baseline in patients who developed thrombocytopenia yet enriched in those experiencing anemia—suggests context-dependent roles that warrant mechanistic investigation, as these seemingly contradictory patterns may reflect different underlying pathophysiological processes predisposing to distinct toxicity phenotypes. These findings highlight potential therapeutic opportunities: supplementing patients with baseline depleted beneficial bacteria (Eubacterium hallii group) might prevent neutropenia, while targeting pro-inflammatory taxa present at baseline (Enterobacteriaceae, Eubacteriaceae) could potentially mitigate inflammatory anemia, though longitudinal studies tracking microbiome changes with toxicity onset and mechanistic studies using gnotobiotic models [38] will be essential to distinguish predictive baseline signatures from treatment-induced dysbiosis and to establish causal relationships.

A distinct pattern of baseline microbiome dysbiosis predicted chemotherapy-induced hepatotoxicity, characterized predominantly by pre-treatment depletion of protective bacteria rather than pathogenic enrichment. These associations suggest but do not prove causal relationships. Akkermansiaceae/Akkermansia showed substantial baseline depletion in patients who subsequently developed AST elevation (d = −0.96), representing the most clinically significant finding given this bacterium’s well-established hepatoprotective properties. Akkermansia muciniphila, which constitutes 1–4% of healthy gut microbiota, exerts hepatoprotection through four interconnected mechanisms. First, A. muciniphila maintains gut barrier integrity by strengthening tight junction proteins (occludin, claudin, ZO-1) and stimulating mucin production, thereby preventing bacterial translocation and reducing systemic lipopolysaccharide exposure to the liver [29,30,31]. This barrier-protective effect is particularly relevant as chemotherapy frequently compromises intestinal epithelial integrity, leading to increased gut permeability and endotoxemia. Second, A. muciniphila produces bioactive metabolites including short-chain fatty acids (propionate, acetate) and the outer membrane protein Amuc_1100, which activate hepatic AMPK signaling, enhance mitochondrial fatty acid oxidation, and reduce hepatic lipid accumulation—collectively improving hepatic metabolic capacity and stress resistance [32,33]. Third, A. muciniphila modulates systemic inflammation by reducing circulating LPS levels and suppressing pro-inflammatory cytokines (TNF-α, IL-6, IL-1β) while promoting anti-inflammatory IL-10 production [34,35]. Fourth, experimental studies demonstrate that A. muciniphila administration protects against various hepatotoxic insults by reducing serum ALT/AST levels (30–50% reduction), attenuating oxidative stress through enhanced glutathione activity, and improving liver fibrosis markers [29,36]. These protective effects persist even after cessation of administration, suggesting sustained microbiome remodeling [30]. The substantial baseline depletion of Akkermansia (d = −0.96) therefore represents loss of multiple protective mechanisms that collectively maintain hepatic resilience against chemotherapy-induced stress. Additional baseline-depleted bacteria predicting hepatotoxicity included Burkholderiaceae (d = −1.19, ALT elevation) and Eubacterium hallii group (d = −0.99, ALT elevation). Eubacterium hallii, a key butyrate-producing bacterium, contributes to hepatoprotection through short-chain fatty acid-mediated enhancement of gut barrier function and anti-inflammatory signaling [37]. Its depletion may compound the loss of Akkermansia’s protective effects, creating a microbiome profile characterized by impaired barrier function, increased endotoxin exposure, and diminished anti-inflammatory capacity. Paradoxically, we observed baseline enrichment of typically beneficial bacteria—Faecalibacterium (d = 0.70) and Streptococcus (d = 0.72)—in patients who developed ALT elevation. For Faecalibacterium, this may represent compensatory proliferation in response to subclinical inflammatory states preceding overt toxicity [38]. The Streptococcus enrichment may reflect oral microbiota translocation predisposing to hepatic inflammation through the oral-gut-liver axis, as increased intestinal Streptococcus colonization has been associated with bacterial translocation and hepatic immune activation [39]. These findings suggest therapeutic opportunities for hepatotoxicity prevention through Akkermansia supplementation in patients identified as high-risk based on baseline microbiome profiling. Preclinical studies demonstrate sustained protective effects even after cessation of administration [30], though clinical trials are needed to establish efficacy, optimal dosing, timing of intervention, and safety in cancer patients receiving hepatotoxic chemotherapy regimens.

Our study identifies novel baseline microbiota signatures predictive of chemotherapy toxicity using rigorous statistical methods with FDR correction and substantial effect sizes (Cohen’s d up to 1.67). However, several important limitations must be acknowledged. First, the cross-sectional design with single-timepoint baseline sampling limits causal inference—while our findings demonstrate predictive associations between pre-treatment microbiota and subsequent toxicity. We cannot definitively establish whether microbiota composition directly influences toxicity susceptibility, whether unmeasured confounders independently affect both microbiota and toxicity, or whether subclinical disease processes alter microbiota before diagnosis [37]. Second, our study lacks systematic collection of important potential confounders, including precise age, body mass index, menopausal status, recent antibiotic or probiotic use, proton pump inhibitor use, other concomitant medications, smoking, alcohol consumption, physical activity, and psychological stress. Most critically, we did not collect detailed dietary data using validated instruments, which is a primary determinant of gut microbiota composition and may independently affect chemotherapy tolerance through nutritional status, inflammation, or metabolic pathways. These unmeasured variables limit our ability to exclude residual confounding, prevent us from demonstrating baseline comparability between adverse event severity groups beyond disease stage and molecular subtype, and restrict causal inference, as they may independently affect both microbiota composition and toxicity susceptibility. Our reported associations are based on comparisons between groups stratified by adverse event severity, with adjustment only for multiple comparisons using FDR correction, and should be interpreted as hypothesis-generating associations requiring validation in studies with comprehensive confounder assessment. Third, our cohort is heavily dominated by sequential anthracycline-taxane regimens (82.7%), with insufficient sample sizes in anthracycline-only (n = 3) and taxane-only (n = 11) groups to perform stratified analyses or meaningful statistical adjustment for regimen type. This limits our ability to assess regimen-specific microbiota-toxicity interactions. The associations we report primarily reflect relationships in patients receiving sequential combination therapy, and we cannot exclude the possibility that different patterns may exist in patients receiving single-agent regimens. Fourth, this single-center study in Taipei, Taiwan, limits generalizability given well-established geographic variation in gut microbiota composition reflecting differences in diet, environment, and ethnicity, requiring validation in diverse populations. Fifth, limited statistical power for rare toxicities constrained detection of associations with modest effect sizes. Sixth, 16S rRNA sequencing provides genus-level resolution only and cannot identify specific bacterial species or strains; functional capacity cannot be directly inferred from taxonomic composition, and we did not analyze fecal metabolites such as short-chain fatty acids or bile acids that may mediate microbiota-host interactions. Seventh, single-timepoint sampling cannot assess microbiota changes during treatment or determine whether longitudinal trajectories predict toxicity better than baseline profiles. Eighth, models were validated internally only; independent external validation in diverse populations is essential before clinical application. Future studies should address these limitations through prospective longitudinal designs with serial sampling, systematic collection of comprehensive baseline data including validated dietary assessments, and balanced recruitment across chemotherapy regimens. Shotgun metagenomic and meta transcriptomic sequencing should be employed for species-level identification and functional pathway analysis, while integrated metabolomic profiling including bile acids and short-chain fatty acids can identify functional mediators, particularly given our findings regarding Eubacteriaceae and butyrate-producing bacteria which are known to influence bile acid metabolism. Mechanistic validation using gnotobiotic models will be essential to test whether baseline microbiota profiles causally determine toxicity risk for candidate taxa, and multi-center studies across diverse geographic populations will assess generalizability. Development of prospective risk stratification models integrating baseline microbiome profiles with clinical variables should be validated in external cohorts, ultimately leading to randomized controlled interventional trials testing targeted microbiome modulation strategies initiated before chemotherapy in patients identified as high-risk based on baseline microbiome profiling [26,37]. Only through such comprehensive approaches addressing these methodological limitations can we translate these baseline microbiome-toxicity predictive associations into clinically actionable risk stratification tools and preventive interventions that improve patient outcomes.

## 5. Conclusions

This study identifies toxicity-specific microbiome signatures that could enable precision prediction and prevention of chemotherapy-related adverse events. However, longitudinal mechanistic studies are essential to establish causality and develop clinically actionable microbiome-based interventions. These findings represent predictive associations rather than proven causal relationships.

## Figures and Tables

**Table 1:** Patient characteristics.

**Clinica T stage**	
cT1	20 (24.7%)
cT2	48 (59.3%)
cT3	9 (11.1%)
cT4	4 (4.9%)
**Clinical N stage**	
cN0	59 (72.8%)
cN+	22 (27.2%)
**Clinical Stage**	
IA	20 (24.7%)
IIA	33 (40.7%)
IIB	15 (18.5%)
IIIA	4 (4.9%)
IIIB	3 (3.7%)
IIIC	1 (1.2%)
IV	5 (6.2%)
**Molecular Subgroup**	
Luminal A	16 (19.8%)
Luminal B	28 (34.6%)
Luminal Her2	12 (14.8%)
Her2	11 (13.6%)
Triple Negative	14 (17.3%)
**Chemotherapy Regimen**	
Anthracycline-based (AC, EC, LipodoxC)	3 (3.7%)
Taxane-based	11 (13.6%)
Anthracycline + taxane sequential	67 (82.7%)
**Chemotherapy Type**	
Neoadjuvant	26 (32.1%)
Adjuvant	50 (61.7%)
Palliative	5 (6.2%)
**Molecular Subgroup of Neoadjuvant Chemotherapy (*n* = 26)**	
Luminal A	2 (7.7%)
Luminal B	9 (34.6%)
Luminal Her2	3 (11.5%)
Her2	4 (15.4%)
Triple Negative	8 (30.8)
**Neoadjuvant chemotherapy (*n* = 26)**	
pCR	7 (26.9%)
No pCR	19 (73.1%)
**Hormone therapy**	
Yes	56 (69.1%)
No	25 (30.9%)
**Recurrence**	
No	70 (86.4%)
Yes	6 (7.4%)
stage IV	5 (6.2%)
pCR: pathologic complete response	
AC: Adriamycin and Cyclophosphamide	
EC: Epirubicin and Cyclophosphamide	
LipodoxC: Liposomal doxorubicin and Cyclophosphamide	

**Table 2:** Chemotherapy side effect grading and patient numbers.

Chemotherapy Side Effect Grading	
**Anemia**	Patient No. (%)
0	29 (35.8)
1	31 (38.3)
2	14 (17.3)
3	7 (8.6)
**Neutropenia**	
0	3 (3.7)
1	5 (6.2)
2	7 (8.6)
3	14 (17.3)
4	52 (64.2)
**Thrombocytopenia**	
0	60 (74.1)
1	20 (24.7)
2	1 (1.2)
**AST(GOT) Elevation**	
0	59 (72.8)
1	20 (24.7)
2	1 (1.2)
3	1 (1.2)
**AST(GOT) Elevation**	
0	42 (51.9)
1	35 (43.2)
2	2 (2.5)
3	2 (2.5)
**Nausea**	
0	58 (71.6)
1	22 (27.2)
2	1 (1.2)
**Oral Mucositis**	
0	68 (84.0)
1	12 (14.8)
2	1 (1.2)
**Diarrhea**	
0	49 (60.5)
1	22 (27.2)
2	10 (12.3)
**Peripheral Neuropathy**	
0	40 (49.4)
1	29 (35.8)
2	7 (8.6)
3	5 (6.2)
**Skin Rash**	
0	63 (77.8)
1	13 (16.0)
2	4 (4.9)
3	1 (1.2)
**Hand Food Syndrome**	
0	70 (86.4)
1	8 (9.9)
2	3 (3.7)
**Fatique**	
0	50 (61.7)
1	28 (34.6)
2	3 (3.7)

**Table 3:** Linear Discriminant Analysis Effect Size (LEfSe) of different side effects in breast cancer patients after Chemotherapy.

Linear Discriminant Analysis of Gut Microbiota Taxa Associated with Chemotherapy-Related Adverse Events							
Adverse Event	Taxonomic Level	Taxa	With AE (Mean)	Without AE (Mean)	LDA Score	Effect Size	Significance
ANEMIA	Family	*Erysipelotrichaceae*	190.1	140.17	2.8	Large	Bio Sig*
ANEMIA	Family	*Clostridiaceae*	3824.2	4147.1	4.37	Large	Bio Sig*
ANEMIA	Genus	*Coprococcus*	194.5	104.52	2.61	Negligible	Nominal Sig
ANEMIA	Genus	*Clostridium*	2812.5	2323.96	2.12	Large	Bio Sig*
ANEMIA	Species	*[Eubacterium]_rectale*	731.7	693.08	1.65	Negligible	Nominal Sig
ANEMIA	Species	*Coprococcus_catus*	1613.9	1534.5	1.6	Negligible	Nominal Sig
ANEMIA	Species	*Eubacterium_sp.*	67.6	58.52	2.35	Large	Bio Sig*
ANEMIA	Species	*Bacteroides_thetaiotaomicron_group*	39,132	15,068	2.87	Large	Bio Sig*
ANEMIA	Species	*Akkermansia_muciniphila*	3770	160.17	2.19	Large	Bio Sig*
ANEMIA	Species	*Massilimicrobium_timonense*	221.24	19.246	2.01	Large	Bio Sig*
ANEMIA	Species	*Clostridium_sporogenes*	330.11	81.004	1.7	Large	Bio Sig*
NEUTROPENIA (Severe)	Family	*Clostridiaceae/Lachnospiraceae_Sedis*	530.56	641.16	−1.75	Medium	Bio Sig*
NEUTROPENIA (Severe)	Genus	*Eubacterium_hallii_group*	4382	2043.4	−3.91	Large	Bio Sig*
NEUTROPENIA (Severe)	Genus	*Roseburia*	266.26	200.66	1.52	Small	Nominal Sig
NEUTROPENIA (Severe)	Species	*Intestinimonas_butyriciproducens*	135.39	683.13	−2.44	Large	Bio Sig*
THROMBOCYTOPENIA	Genus	*Eubacterium*	39.127	2055	−3	Large	Bio Sig*
THROMBOCYTOPENIA	Species	*Blautia_luti/Faecalibacterium_prausnitzii*	30.127	2055	−3	Large	Bio Sig*
AST(GOT) ELEVATION	Family	*Akkermansiaceae*	7241.4	2961.2	−4.03	Large	Bio Sig*
AST(GOT) ELEVATION	Genus	*Ruminococcaceae*	167,140	92,946	4.58	Small	Nominal Sig
AST(GOT) ELEVATION	Genus	*Bacteroidaceae*	348,710	469,440	−4.85	Small	Nominal Sig
AST(GOT) ELEVATION	Genus	*Butyricicoccus*	3273.4	1181.7	3.02	Medium	Bio Sig*
AST(GOT) ELEVATION	Genus	*Akkermansia*	7241.4	2961.2	−4.03	Large	Bio Sig*
AST(GOT) ELEVATION	Species	*Subdoligranulum_variabile*	1790.1	3840.3	3.82	Large	Bio Sig*
AST(GOT) ELEVATION	Species	*metagenome*	7560.1	881.63	3.52	Large	Bio Sig*
ALT(GPT) ELEVATION	Family	*Burkholderiaceae*	65.263	19.485	−3.65	Medium	Bio Sig*
ALT(GPT) ELEVATION	Genus	*Haemilus*	864.79	810.78	−2.89	Negligible	Nominal Sig
ALT(GPT) ELEVATION	Genus	*Acidaminococcaceae*	16243	32,737	−3.92	Medium	Bio Sig*
ALT(GPT) ELEVATION	Genus	*Eubacterium_hallii_group*	3949.8	1582.8	−3.77	Large	Bio Sig*
ALT(GPT) ELEVATION	Genus	*Burkholderia_Caballeronia_Para*	172.99	1039.3	−2.64	Large	Bio Sig*
NAUSEA	Species	*Intestinimonas_butyriciproducens*	449.56	17.621	2.34	Large	Bio Sig*
NAUSEA	Species	*Christensenella_minuta*	62.35	0	1.51	Large	Bio Sig*
NAUSEA	Species	*Faecalibacterium_prausnitzii*	1296.3	159.51	2.76	Large	Bio Sig*
NAUSEA	Species	*Bacteroides_coprocola_DSM_17136*	27,895	72,430	−4.35	Medium	Not Sig
ORAL MUCOSITIS	Genus	*Coprococcus_2*	3366.8	614	3.14	Large	Bio Sig*
ORAL MUCOSITIS	Genus	*Ruminococcaceae_UCG_014*	6478.8	0	3.51	Large	Bio Sig*
ORAL MUCOSITIS	Species	*guL_metagenome*	33196	12369	4.02	Medium	Bio Sig*
DIARRHEA	Genus	*Christensenella*	72.482	3.5995	1.35	Large	Bio Sig*
DIARRHEA	Species	*Christensenella_minuta*	72.482	3.5995	1.35	Large	Bio Sig*
PERIPHERAL NEUROPATHY	Species	*Clostridium_scindens*	122.67	51.06	1.57	Medium	Bio Sig*
SKIN RASH	Species	*Bacteroides_ovatus*	1111.8	852.16	2.12	Small	Nominal Sig
HAND-FOOT SYNDROME	Family	*Erysipelotrichaceae*	125.09	188.11	−3.77	Large	Bio Sig*
HAND-FOOT SYNDROME	Genus	*Lachnospiraceae*	115490	206780	−4.61	Small	Nominal Sig
HAND-FOOT SYNDROME	Genus	*Butyricicoccus*	1703.5	8535.8	−3.5	Small	Nominal Sig
HAND-FOOT SYNDROME	Genus	*Roseburia*	5545.1	2057.1	−3.88	Large	Small & Bio Sig***
FATIGUE	Species	*Bacteroides_vulgatus*	39121	72232	−4.22	Small	Nominal Sig
**LEGEND**							
**Significance Categories:**							
Bio Sig* (Biologically Significant)	FDR *p* < 0.05 AND Cohen’s d > 0.5—Statistically robust AND biologically meaningful						
Nominal Sig	*p* < 0.05, d ≤ 0.5 (FDR *p* ≥ 0.05)—Statistically significant but small effect						
Small & Bio Sig***	*p* < 0.05, d ≥ 0.05 (Cohen’s d > 0.5)—Nominal biological relevance						
Not Sig	*p* ≥ 0.05—No statistical significance						

## Data Availability

The data for this study is available at https://www.ncbi.nlm.nih.gov/bioproject/PRJNA953204/ (accessed on 7 April 2023). Project ID: PRJNA953204.

## Supplementary Materials

The following supporting information can be downloaded at https://www.mdpi.com/article/10.3390/cancers17233783/s1, Figure S1: Gut microbiome diversity analysis comparing patients with mild versus severe anemia. Figure S2: Gut microbiome diversity analysis comparing patients with mild versus severe neutropenia. Figure S3: Gut microbiome diversity analysis comparing patients with and without thrombocytopenia. Figure S4: Gut microbiome diversity analysis comparing patients with and without ALT elevation. Figure S5: Gut microbiome diversity analysis comparing patients with and without AST elevation. Figure S6: Gut microbiome diversity analysis comparing patients with and without nausea. Figure S7: Gut microbiome diversity analysis comparing patients with and without oral mucositis. Gure S8: Gut microbiome diversity analysis comparing patients with and without diarrhea. Figure S9: Gut microbiome diversity analysis comparing patients with and without peripheral sensory neuropathy. Figure S10: Gut microbiome diversity analysis comparing patients with and without maculopapular rash. Figure S11: Gut microbiome diversity analysis comparing patients with and without hand-foot syndrome. Figure S12: Gut microbiome diversity analysis comparing patients with and without fatigue.

## Abbreviations

The following abbreviations are used in this manuscript:

ALT	Alanine Aminotransferase
ANC	Absolute Neutrophil Count
ANOVA	Analysis of Variance
AST	Aspartate Aminotransferase
BRCA	Breast Cancer gene
CI	Confidence Interval
CTCAE	Common Terminology Criteria for Adverse Events
DNA	Deoxyribonucleic Acid
FDR	False Discovery Rate
GOT	Glutamic-Oxaloacetic Transaminase
GPT	Glutamic-Pyruvic Transaminase
HER2	Human Epidermal growth factor Receptor 2
HFS	Hand-Foot Syndrome
HR	Hormone Receptor
IL-1β	Interleukin-1 beta
IL-6	Interleukin-6
IRB	Institutional Review Board
LDA	Linear Discriminant Analysis
LEfSe	Linear discriminant analysis Effect Size
LPS	Lipopolysaccharide
MMH	MacKay Memorial Hospital
NCBI	National Center for Biotechnology Information
NCDB	National Cancer Database
NSTC	National Science and Technology Council
OTU	Operational Taxonomic Unit
pCR	Pathologic Complete Response
PCR	Polymerase Chain Reaction
PCoA	Principal Coordinates Analysis
PERMANOVA	Permutational Multivariate Analysis of Variance
PSN	Peripheral Sensory Neuropathy
rRNA	Ribosomal Ribonucleic Acid
SCFAs	Short-Chain Fatty Acids
SD	Standard Deviation
TDM-1	Trastuzumab emtansine
TNF-α	Tumor Necrosis Factor alpha
ULN	Upper Limit of Normal
