# Supplementary material for: Gut Microbiota Signatures Associate with Chemotherapy-Related Adverse Events in Breast Cancer Patients
Source: Cancers (Basel). 2025 Nov 26;17(23):3783. doi: 10.3390/cancers17233783 (PMC12691247; doi:10.3390/cancers17233783)

Supplementary Fig S1a

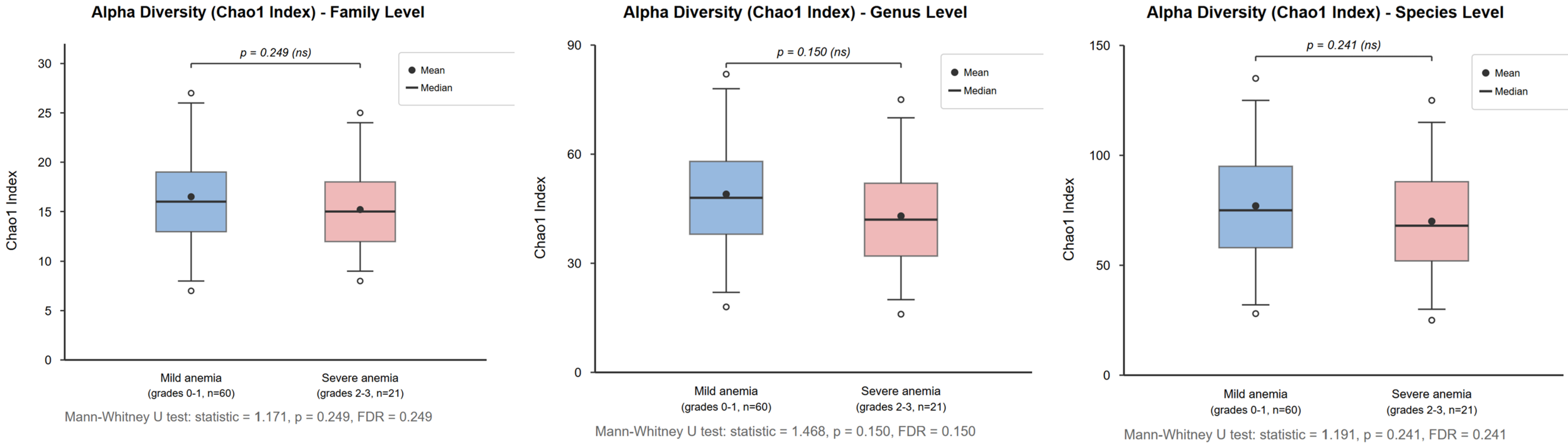

Supplementary Fig S1b

Beta Diversity Analysis: Group F

Principal Coordinates Analysis (PCoA)

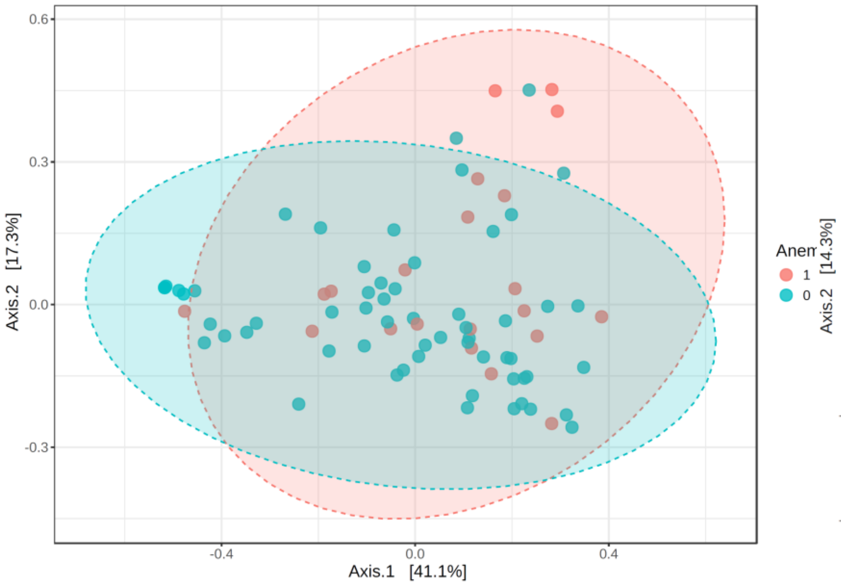

PERMANOVA Results

Comparison: 1 vs 0

Statistic	F	R-squared	P-value	FDR
1 vs 0	2.4999	0.030673	0.034*	0.034

* P < 0.05 (Significant difference between groups)

Beta Diversity Analysis: Group G

Principal Coordinates Analysis (PCoA)

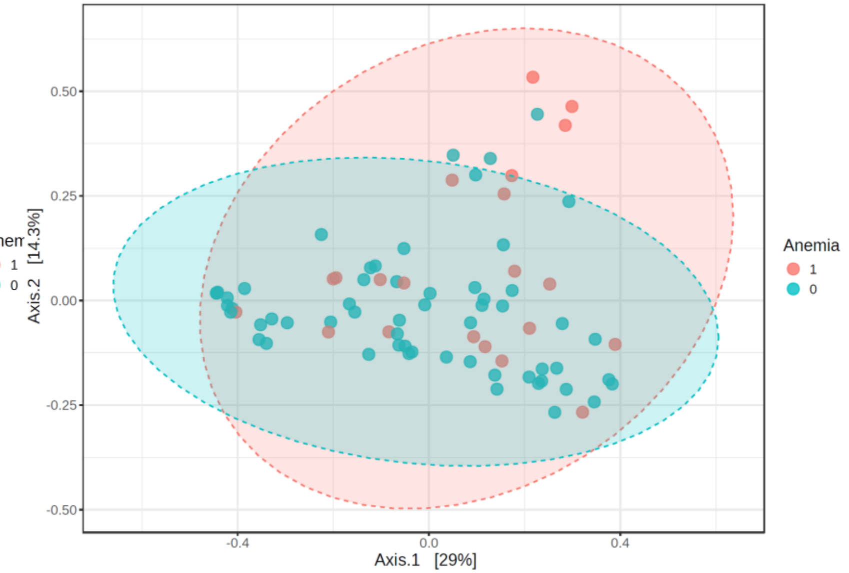

PERMANOVA Results

Comparison: 1 vs 0

Statistic	F	R-squared	P-value	FDR
1 vs 0	2.2008	0.027103	0.027*	0.027

* P < 0.05 (Significant difference between groups)

Beta Diversity Analysis: Group S

Principal Coordinates Analysis (PCoA)

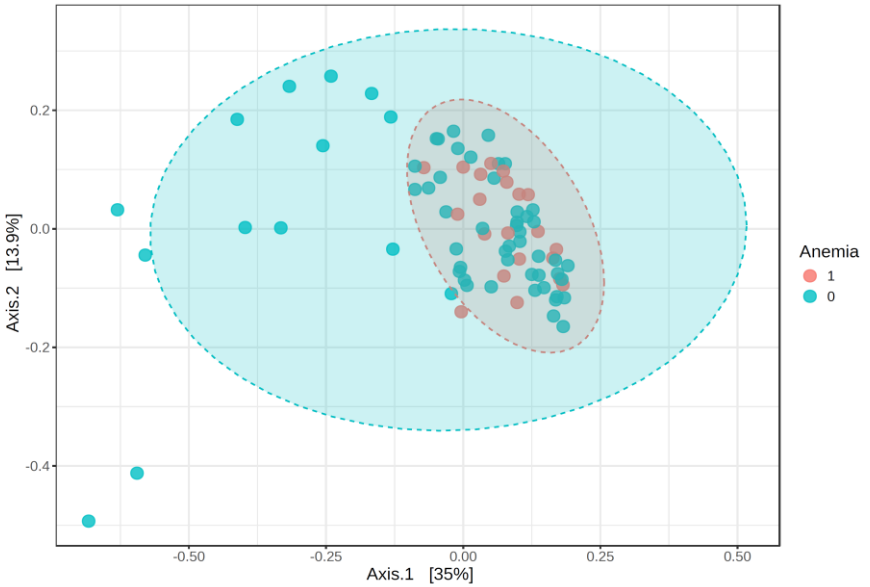

PERMANOVA Results

Comparison: 1 vs 0

Statistic	F	R-squared	P-value	FDR
1 vs 0	1.9367	0.023929	0.057 ns	0.057

ns = not significant (P > 0.05), trending toward significance

Supplementary Fig S1c

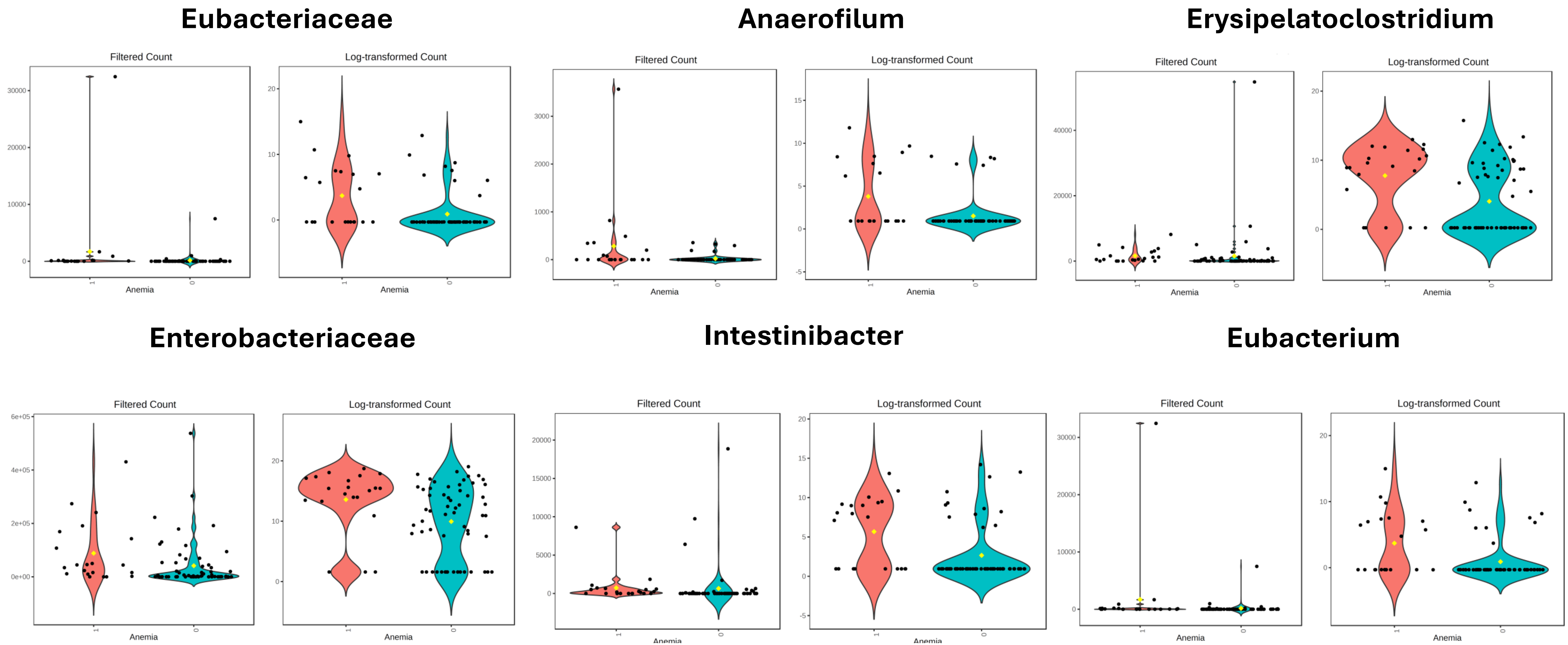

Supplementary Fig S2a

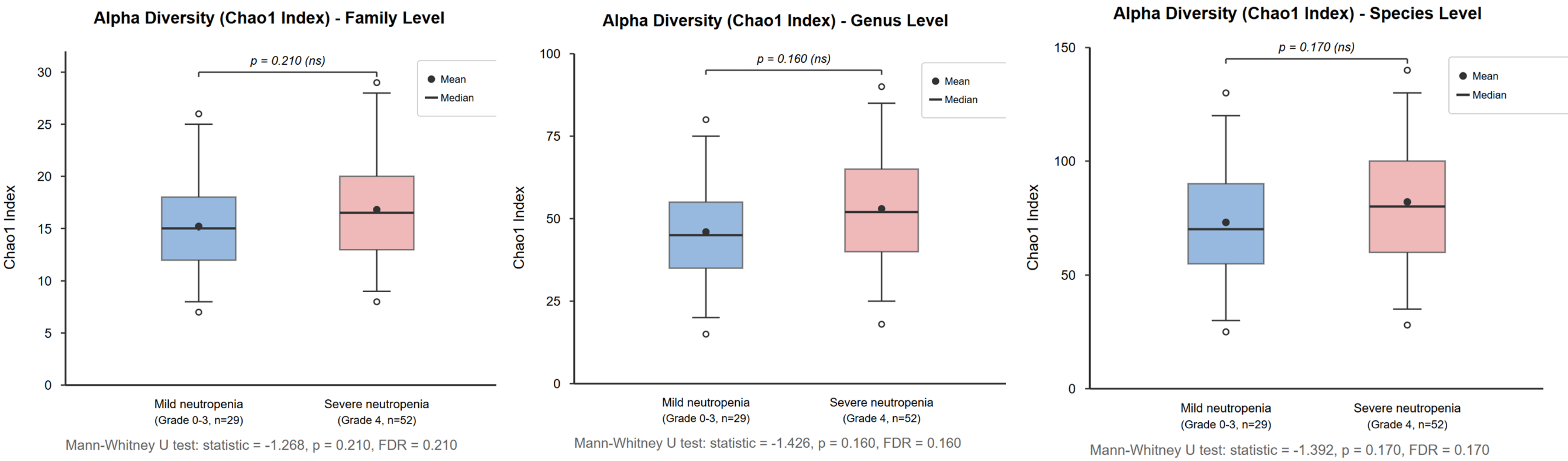

Supplementary Fig S2b

Beta Diversity Analysis: Group F

Principal Coordinates Analysis (PCoA)

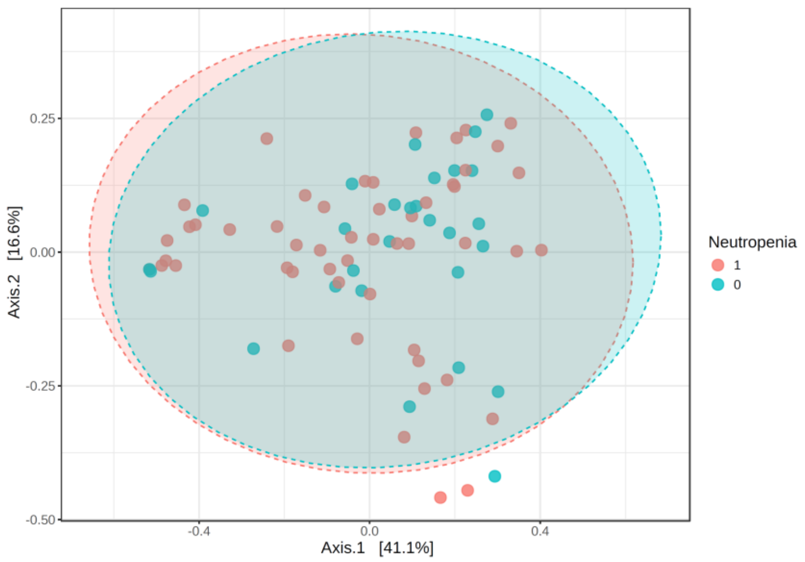

PERMANOVA Results

Comparison: 1 vs 0

Statistic	F	R-squared	P-value	FDR
1 vs 0	2.4999	0.030673	0.034*	0.034

** P < 0.05 (Significant difference between groups)*

Beta Diversity Analysis: Group G

Principal Coordinates Analysis (PCoA)

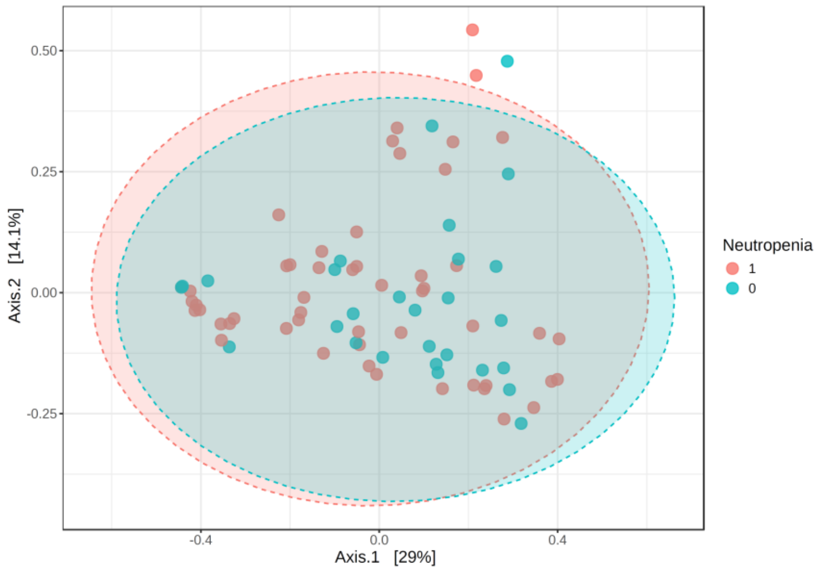

PERMANOVA Results

Comparison: 1 vs 0

Statistic	F	R-squared	P-value	FDR
1 vs 0	2.2008	0.027103	0.027*	0.027

** P < 0.05 (Significant difference between groups)*

Beta Diversity Analysis: Group S

Principal Coordinates Analysis (PCoA)

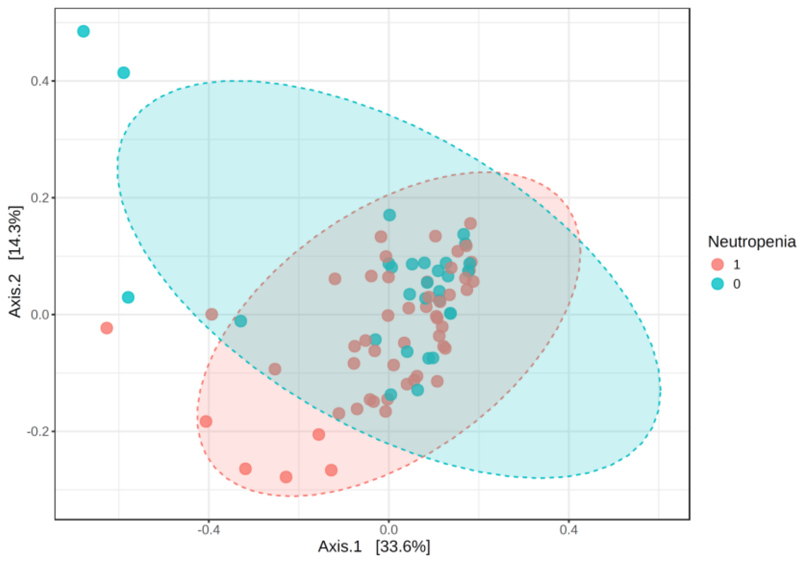

PERMANOVA Results

Comparison: 1 vs 0

Statistic	F	R-squared	P-value	FDR
1 vs 0	1.9367	0.023929	0.057 ns	0.057

ns = not significant (P > 0.05), trending toward significance

Supplementary Fig S2c

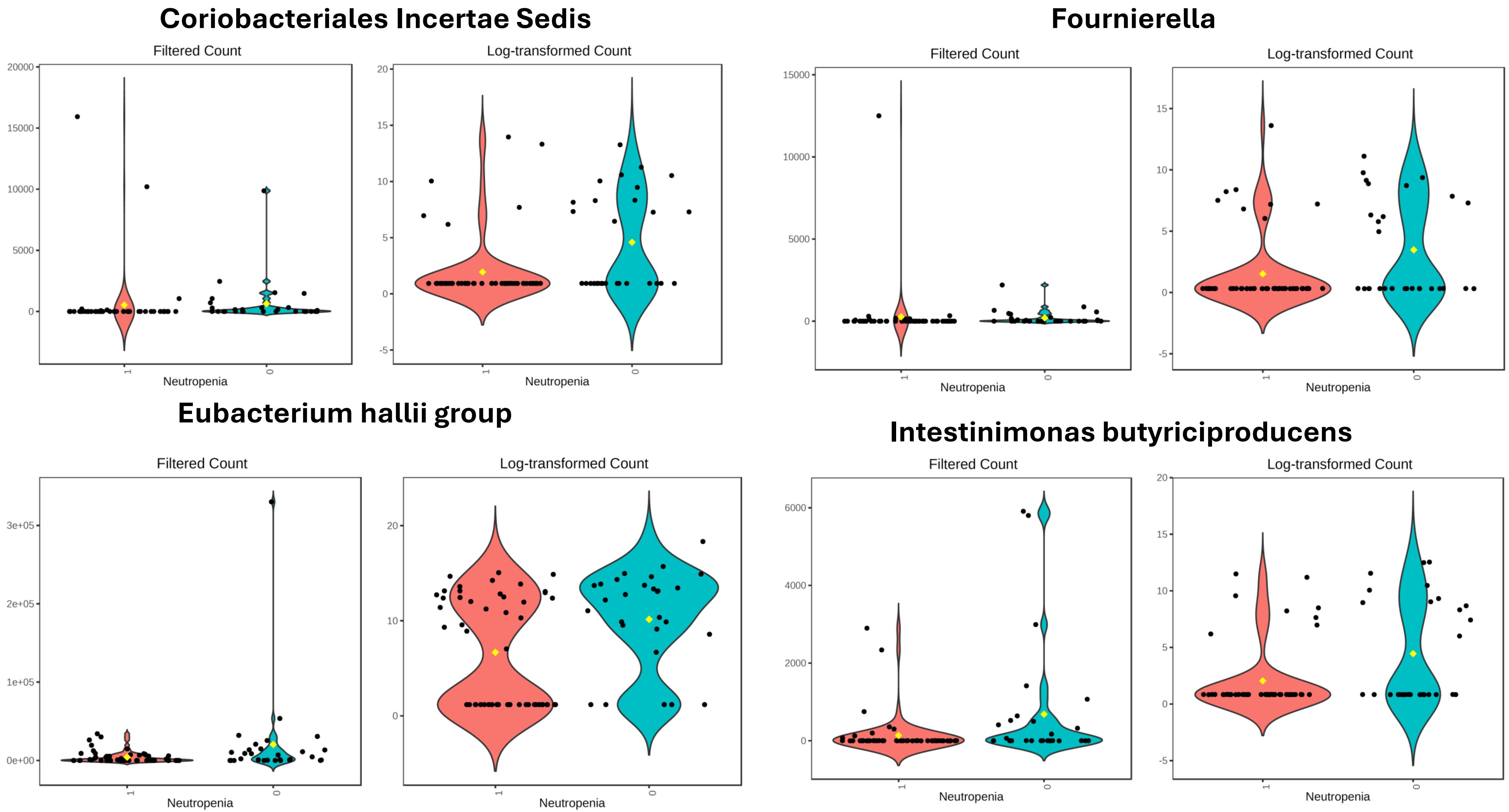

Supplementary Fig S3a

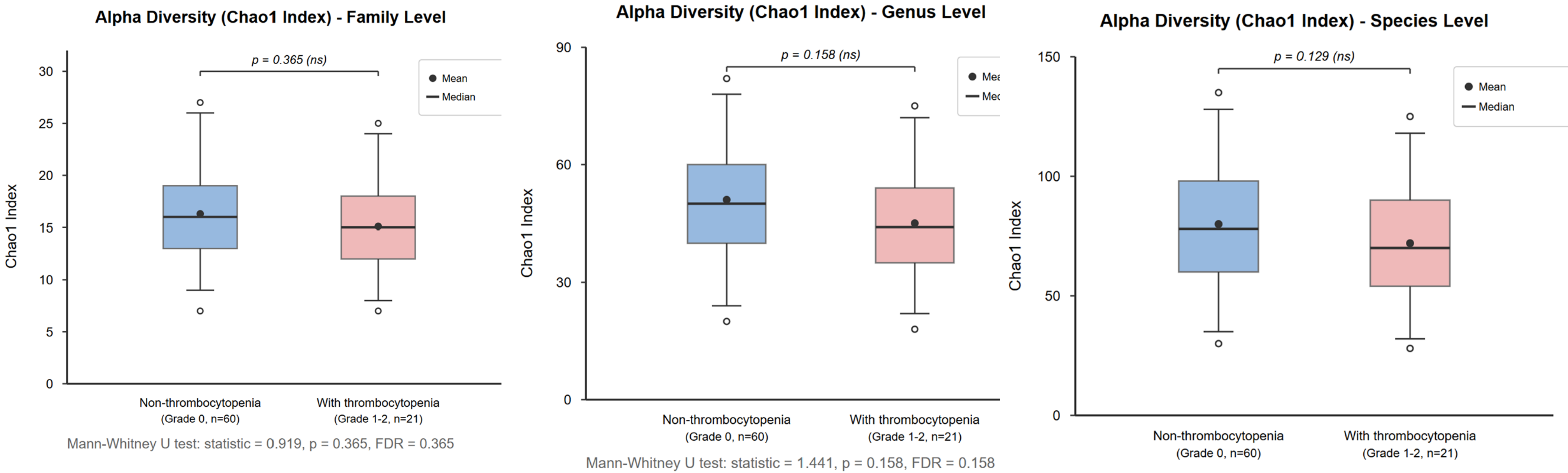

Supplementary Fig S3b

Beta Diversity Analysis: Group F

Principal Coordinates Analysis (PCoA)

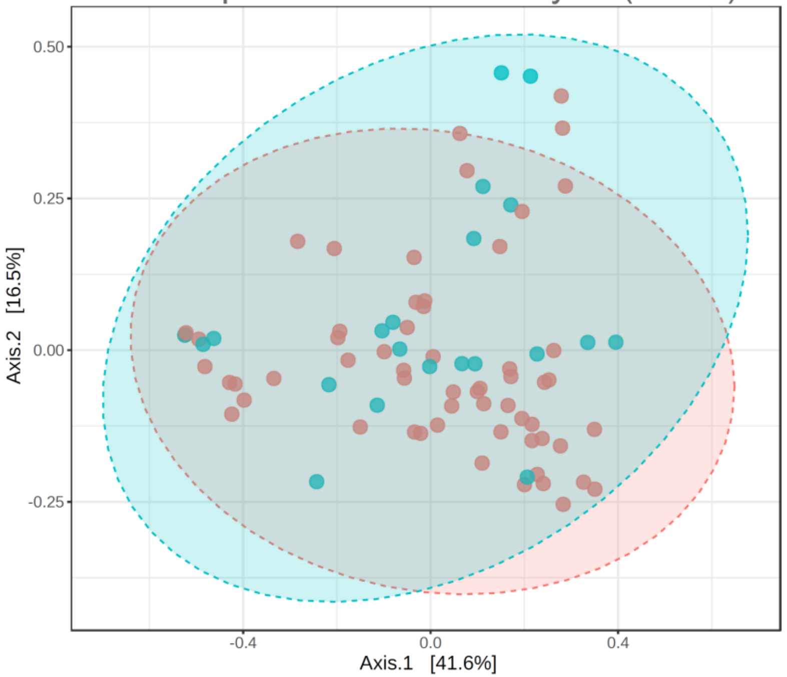

PERMANOVA Results

Comparison: 0 vs 1

Statistic	F	R-squared	P-value	FDR
0 vs 1	0.9424	0.0118	0.433	0.433

No significant difference between groups (P > 0.05)

Beta Diversity Analysis: Group G

Principal Coordinates Analysis (PCoA)

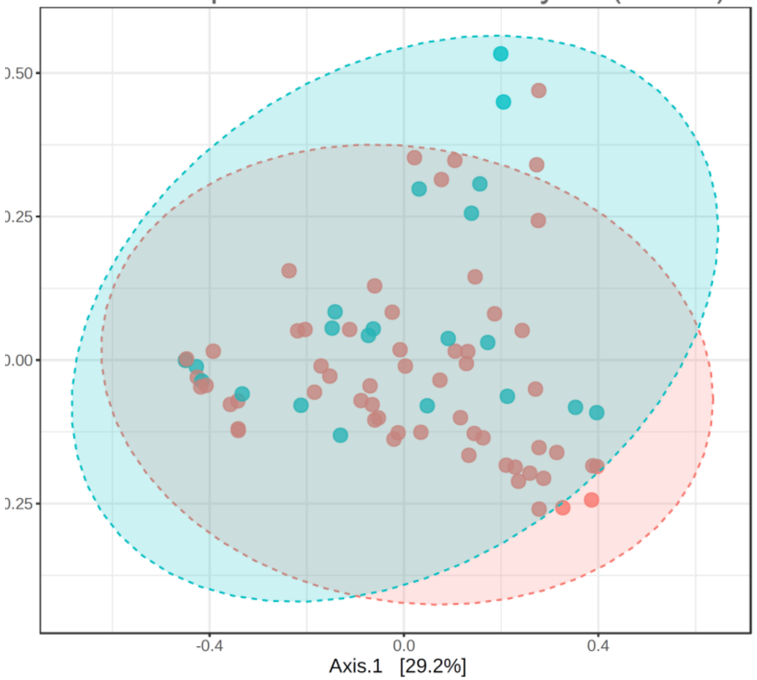

PERMANOVA Results

Comparison: 0 vs 1

Statistic	F	R-squared	P-value	FDR
0 vs 1	1.1318	0.0141	0.299	0.299

No significant difference between groups (P > 0.05)

Beta Diversity Analysis: Group S

Principal Coordinates Analysis (PCoA)

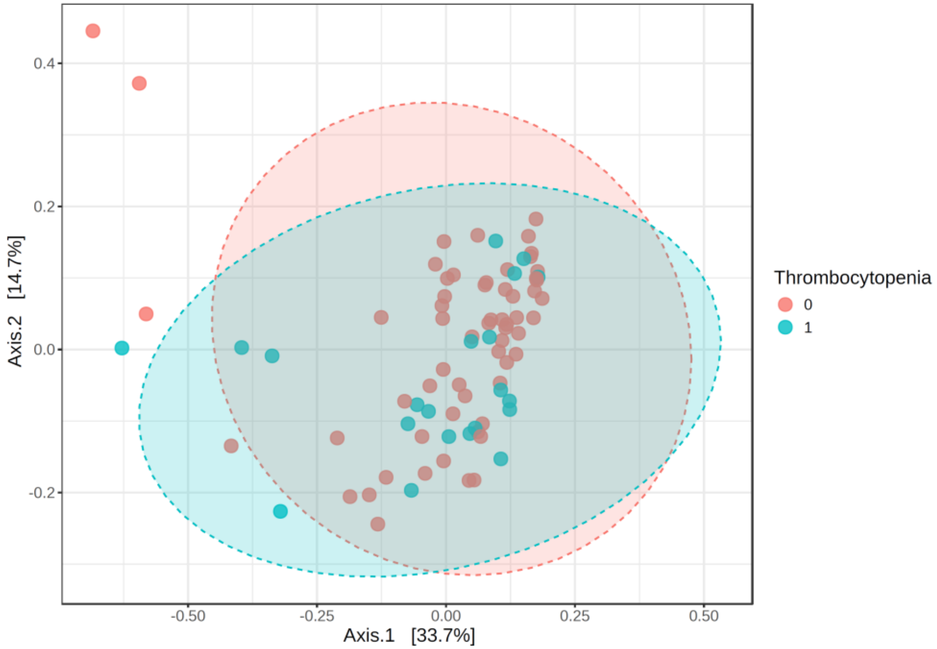

PERMANOVA Results

Comparison: 0 vs 1

Statistic	F	R-squared	P-value	FDR
0 vs 1	1.3055	0.0163	0.226	0.226

No significant difference between groups (P > 0.05)

Supplementary Fig S3c

Eubacterium

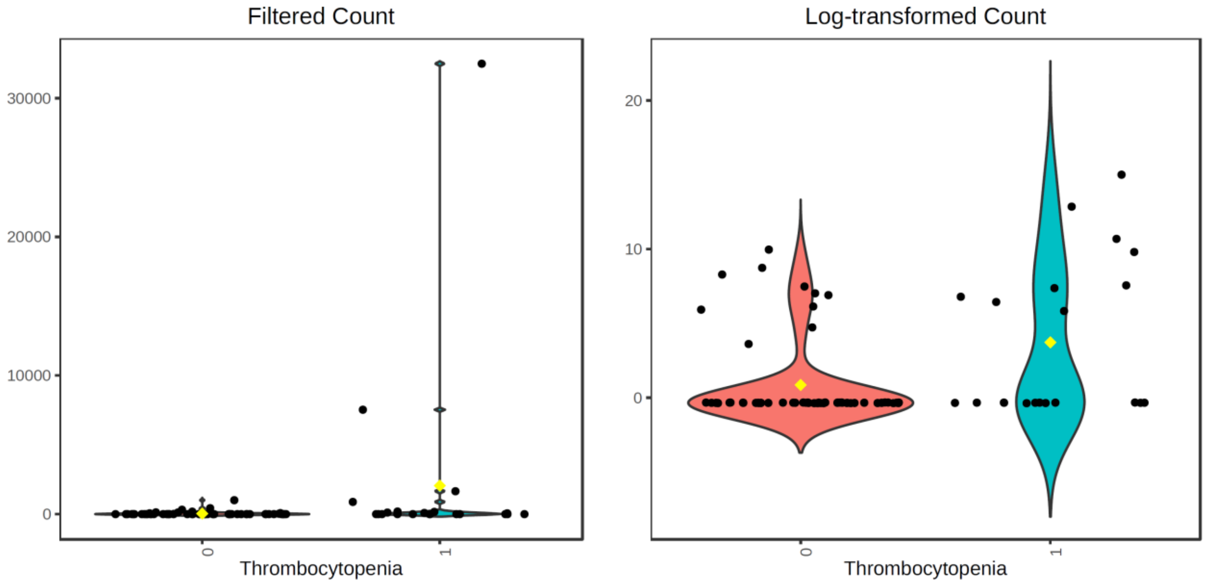

Eubacterium_limosum

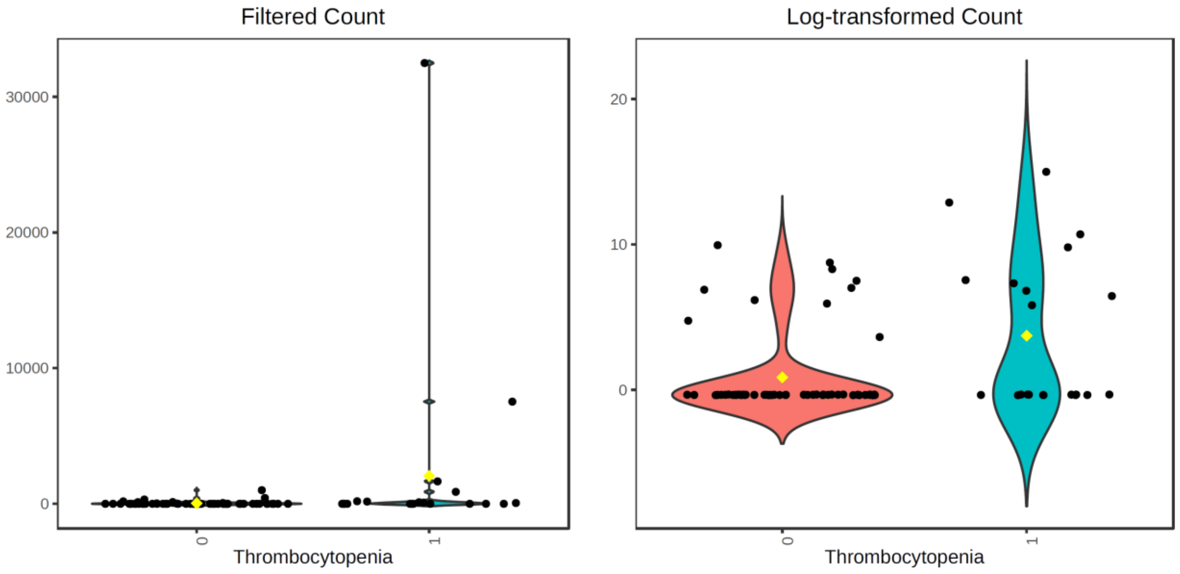

Supplementary Fig S4a

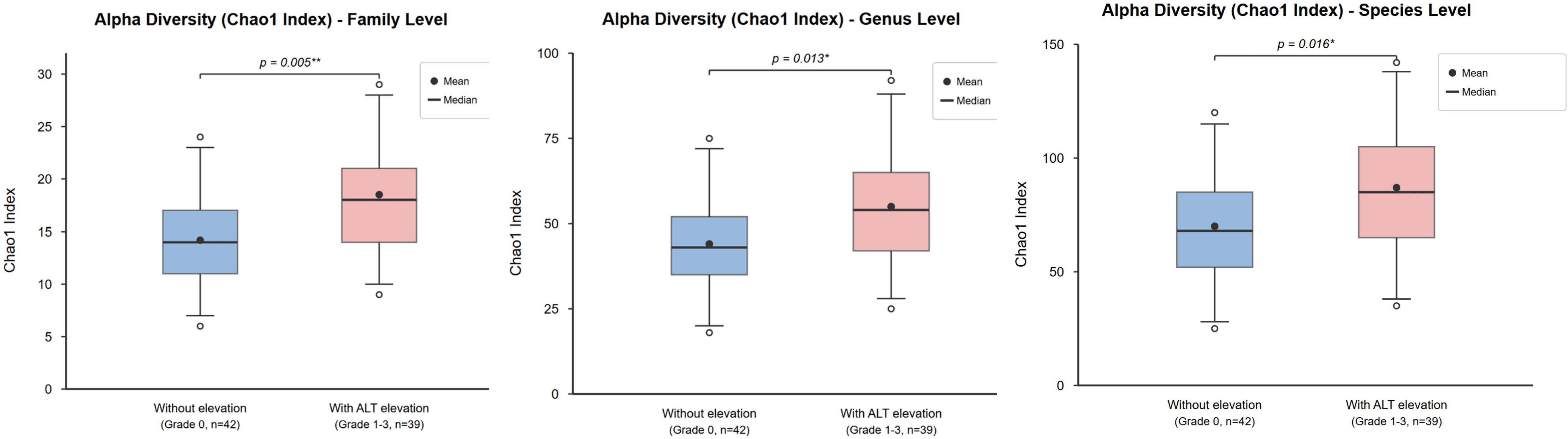

Mann-Whitney U test: statistic = -2.912, p = 0.005, FDR = 0.005

Mann-Whitney U test: statistic = -2.559, p = 0.013, FDR = 0.013

Mann-Whitney U test: statistic = -2.462, p = 0.016, FDR = 0.016

Supplementary Fig S4b

Beta Diversity Analysis: Group F

Principal Coordinates Analysis (PCoA)

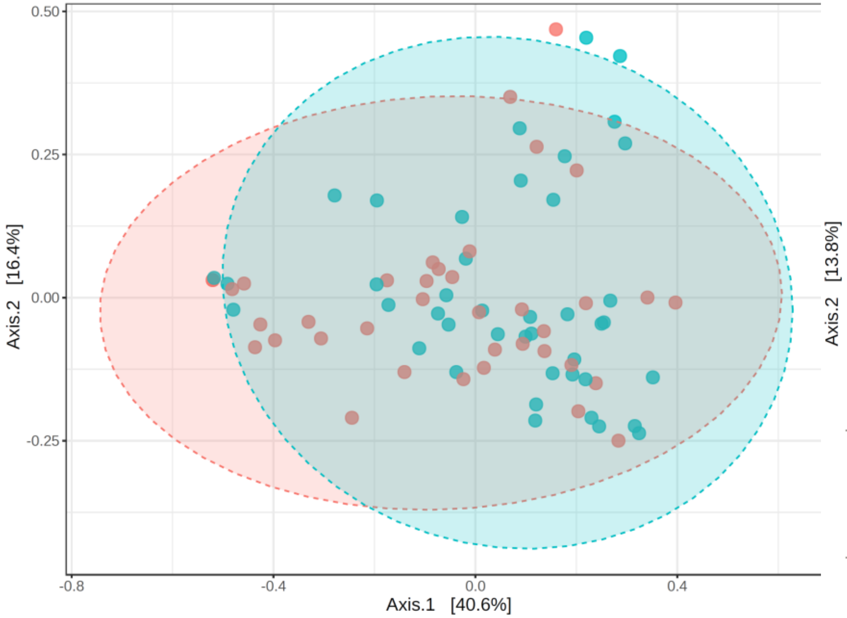

PERMANOVA Results

Comparison: 1 vs 0

Statistic	F	R-squared	P-value	FDR
1 vs 0	2.8538	0.0349	0.031*	0.031

** P < 0.05 (Significant difference between groups)*

Beta Diversity Analysis: Group G

Principal Coordinates Analysis (PCoA)

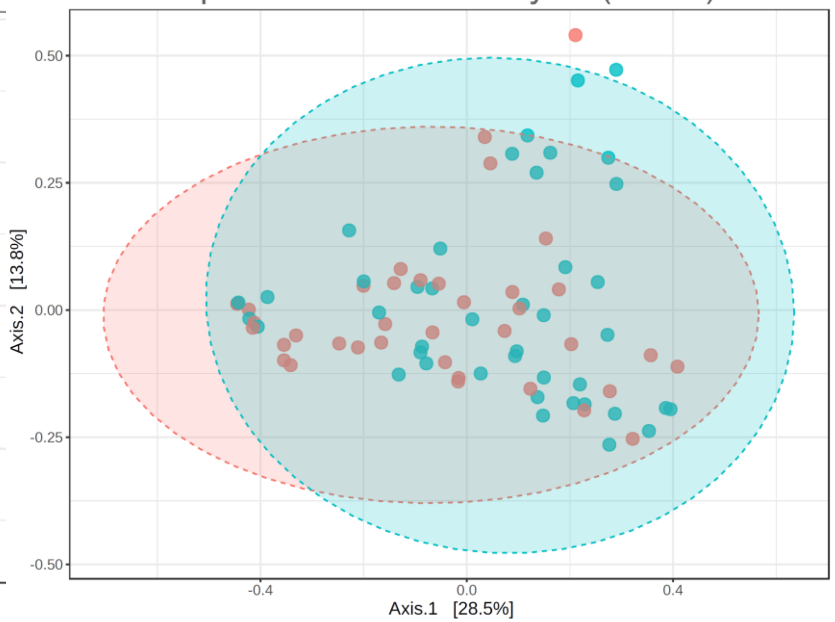

PERMANOVA Results

Comparison: 1 vs 0

Statistic	F	R-squared	P-value	FDR
1 vs 0	2.6372	0.0323	0.020*	0.020

** P < 0.05 (Significant difference between groups)*

Beta Diversity Analysis: Group S

Principal Coordinates Analysis (PCoA)

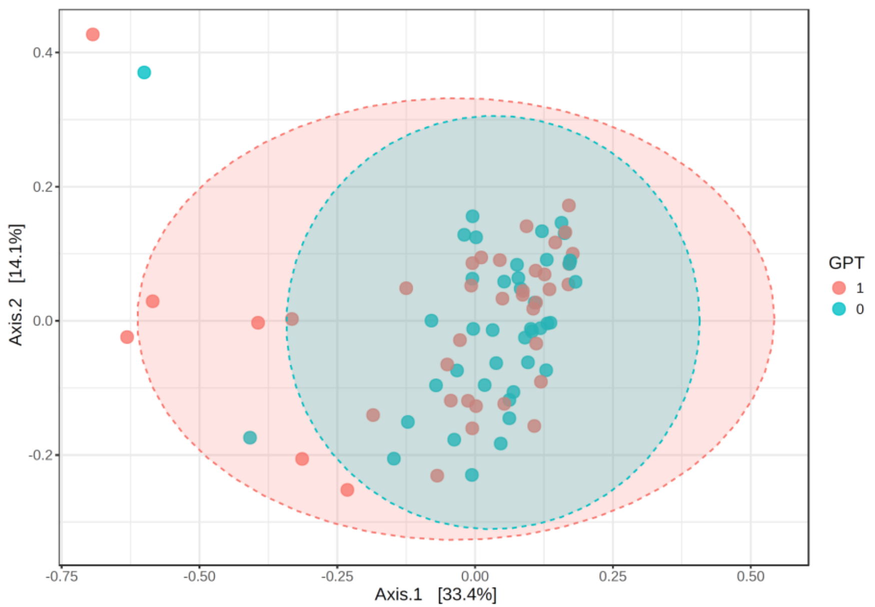

PERMANOVA Results

Comparison: 1 vs 0

Statistic	F	R-squared	P-value	FDR
1 vs 0	1.3789	0.0172	0.202	0.202

No significant difference between groups (P > 0.05)

Supplementary Fig S4c

Burkholderiaceae

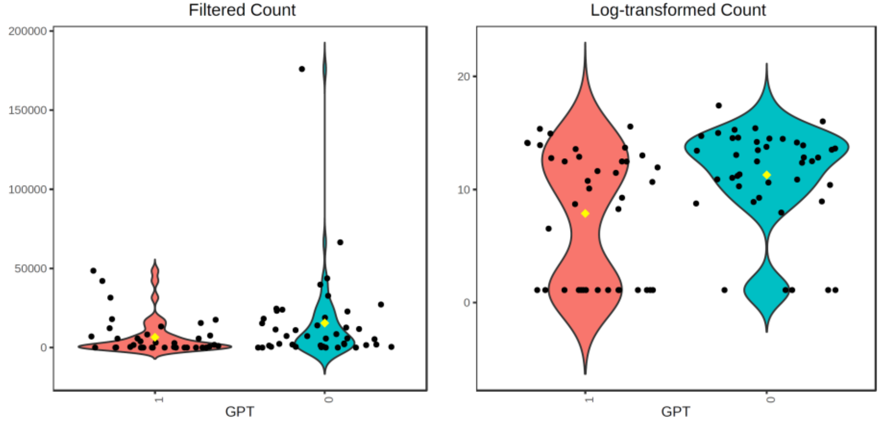

Marinifilaceae

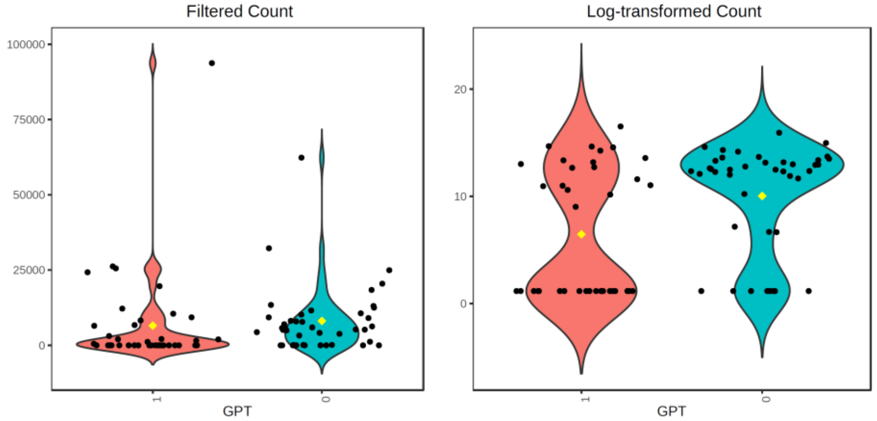

Acidaminococcaceae

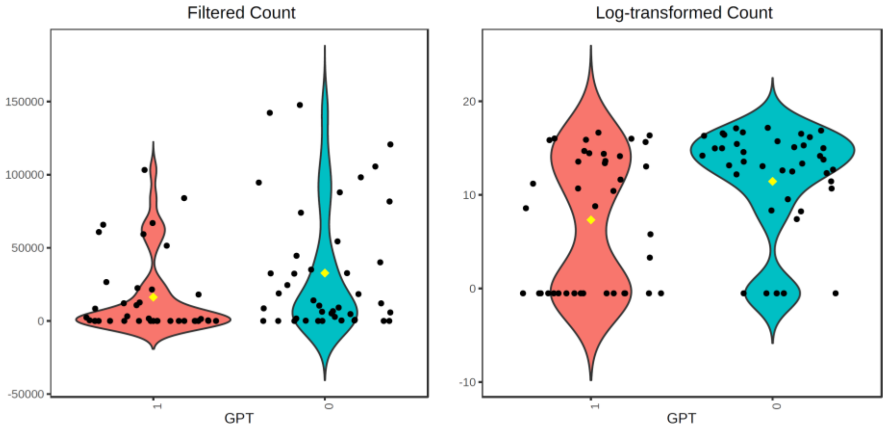

Eubacterium hallii group

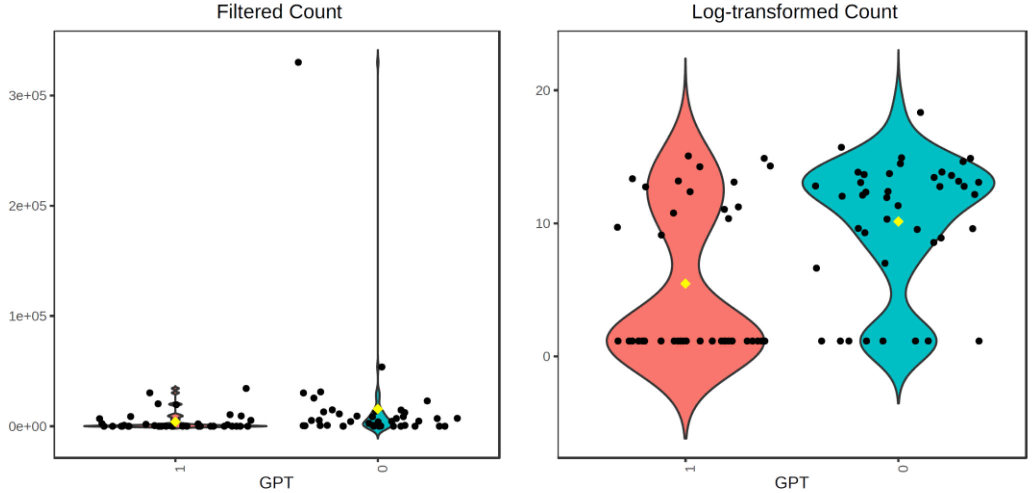

Burkholderia Caballeronia Paraburkholderia

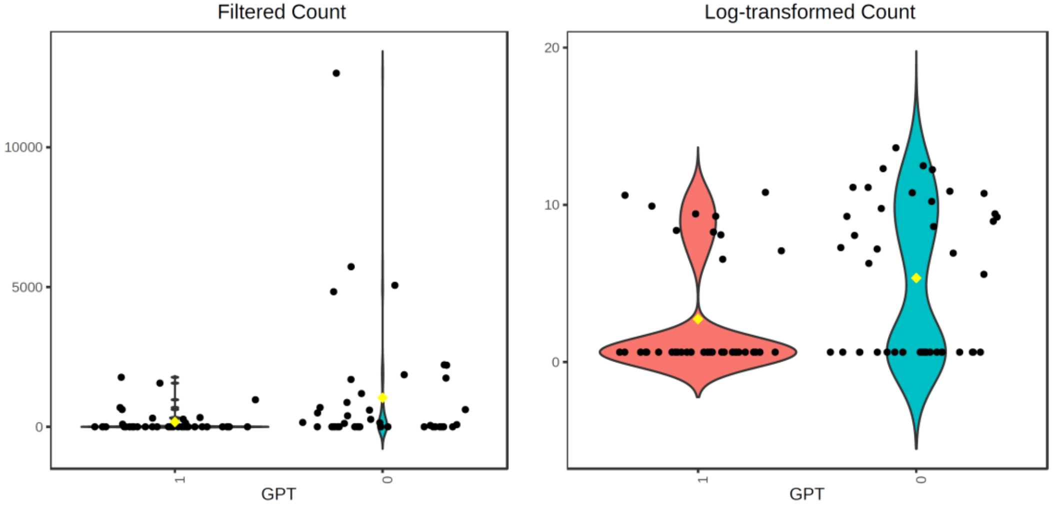

Supplementary Fig S5a

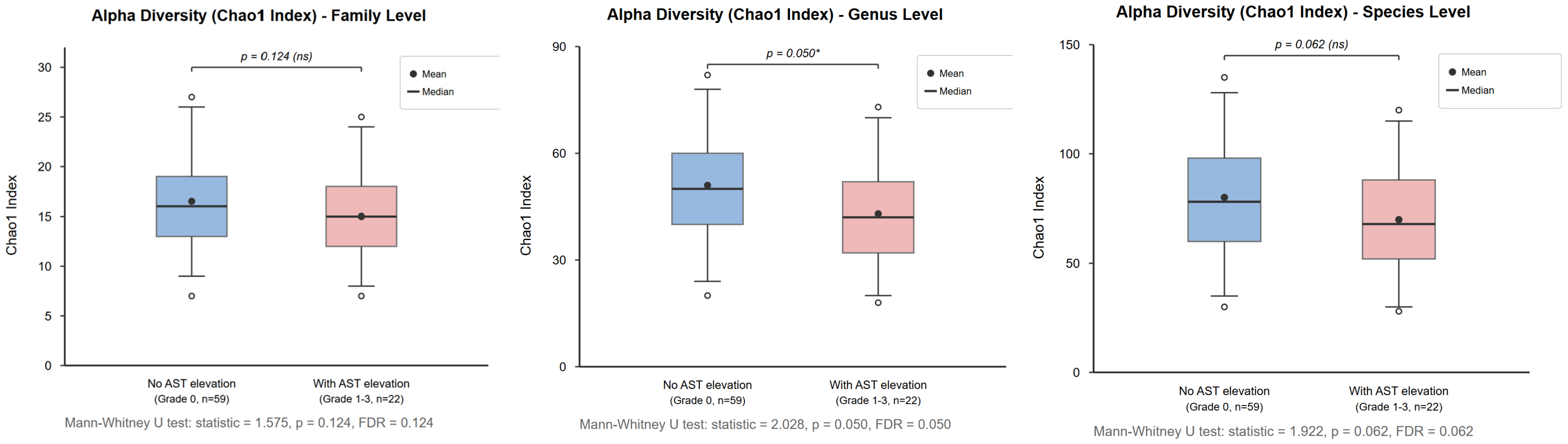

Supplementary Fig S5b

Beta Diversity Analysis: Group F

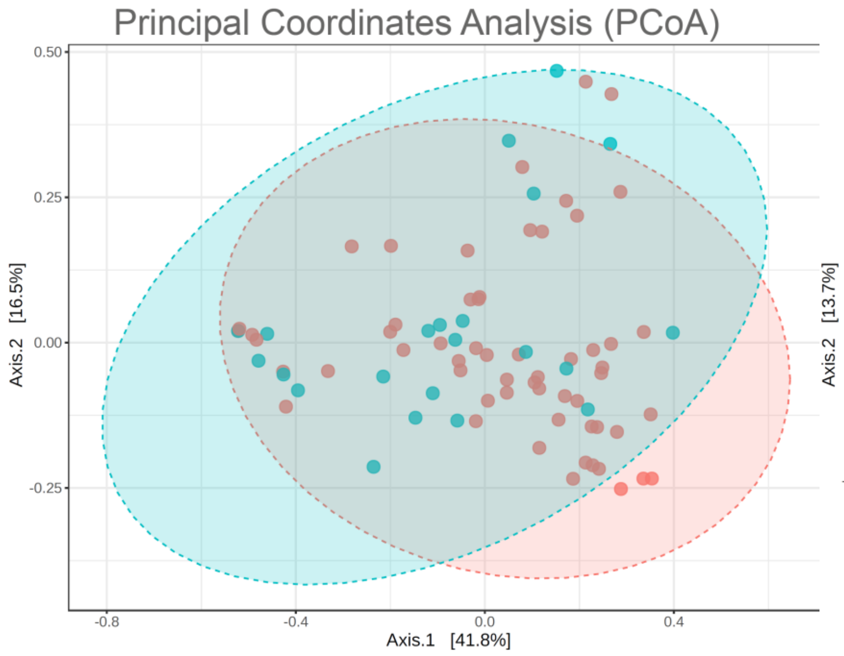

PERMANOVA Results

Comparison: 0 vs 1

Statistic	F	R-squared	P-value	FDR
0 vs 1	2.7713	0.0339	0.023*	0.023

* P < 0.05 (Significant difference between groups)

Beta Diversity Analysis: Group G

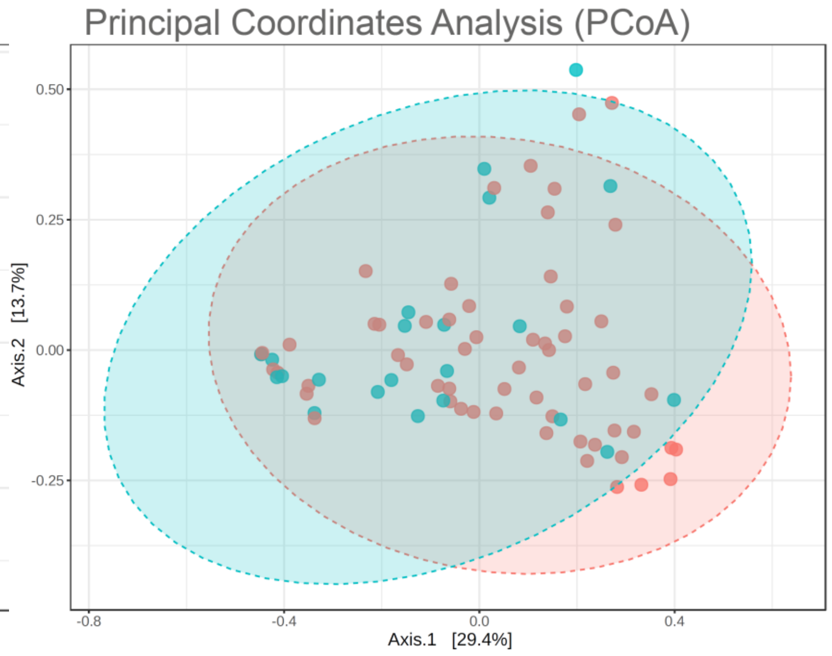

PERMANOVA Results

Comparison: 0 vs 1

Statistic	F	R-squared	P-value	FDR
0 vs 1	2.2415	0.0276	0.027*	0.027

* P < 0.05 (Significant difference between groups)

Beta Diversity Analysis: Group S

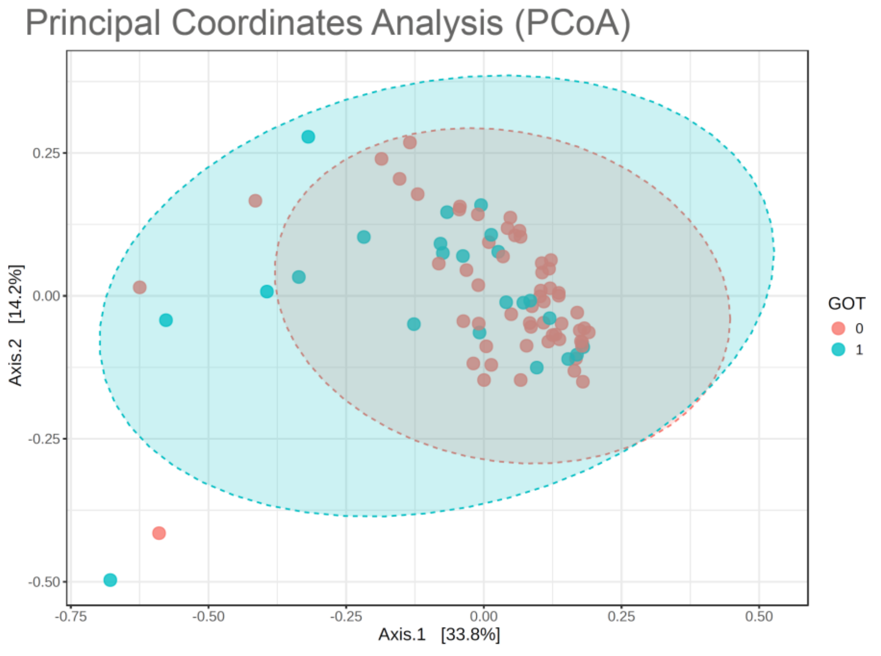

PERMANOVA Results

Comparison: 0 vs 1

Statistic	F	R-squared	P-value	FDR
0 vs 1	2.9475	0.0360	0.012*	0.012

* P < 0.05 (Significant difference between groups)

Supplementary Fig S5c

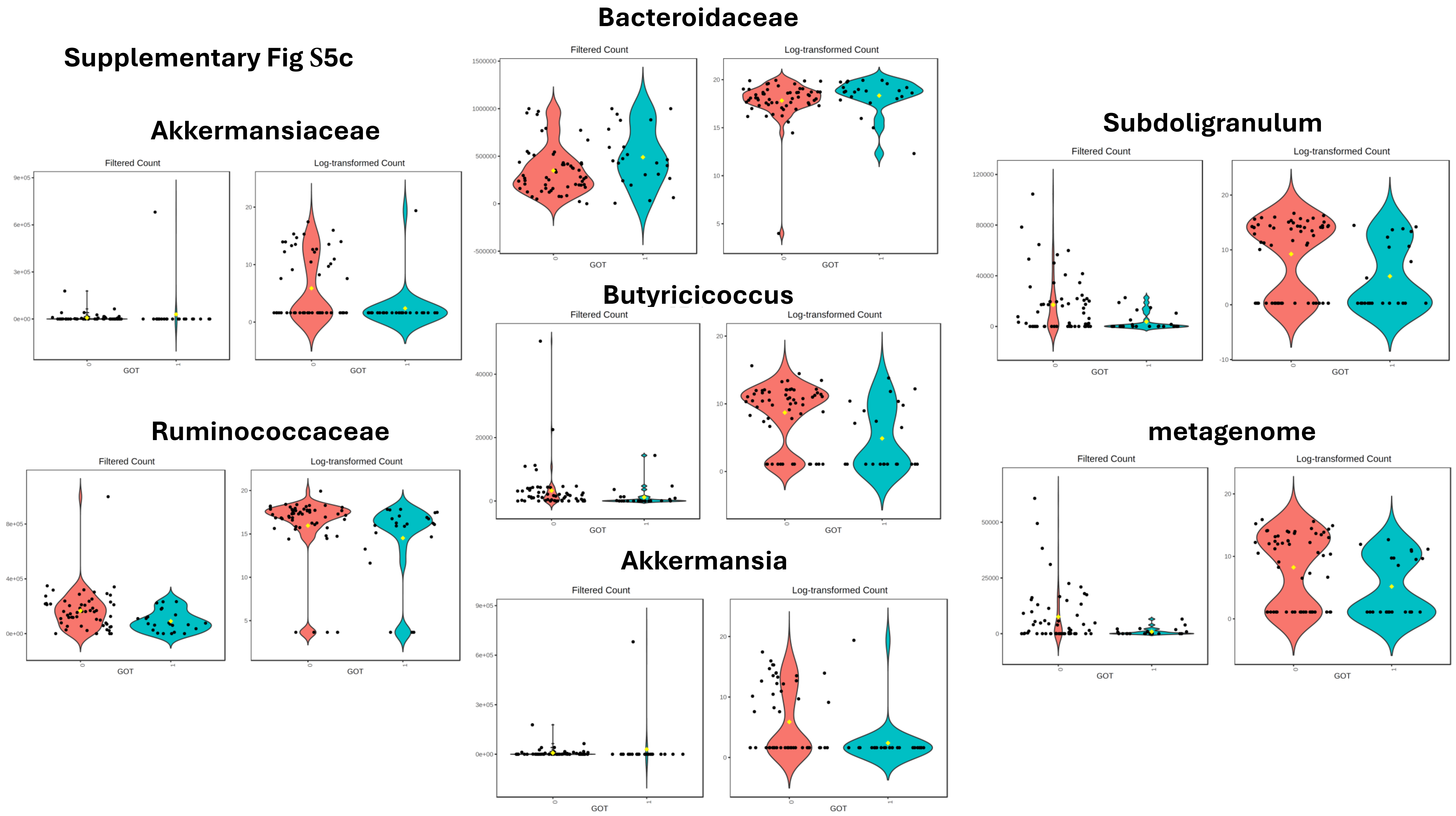

Supplementary Fig S6a

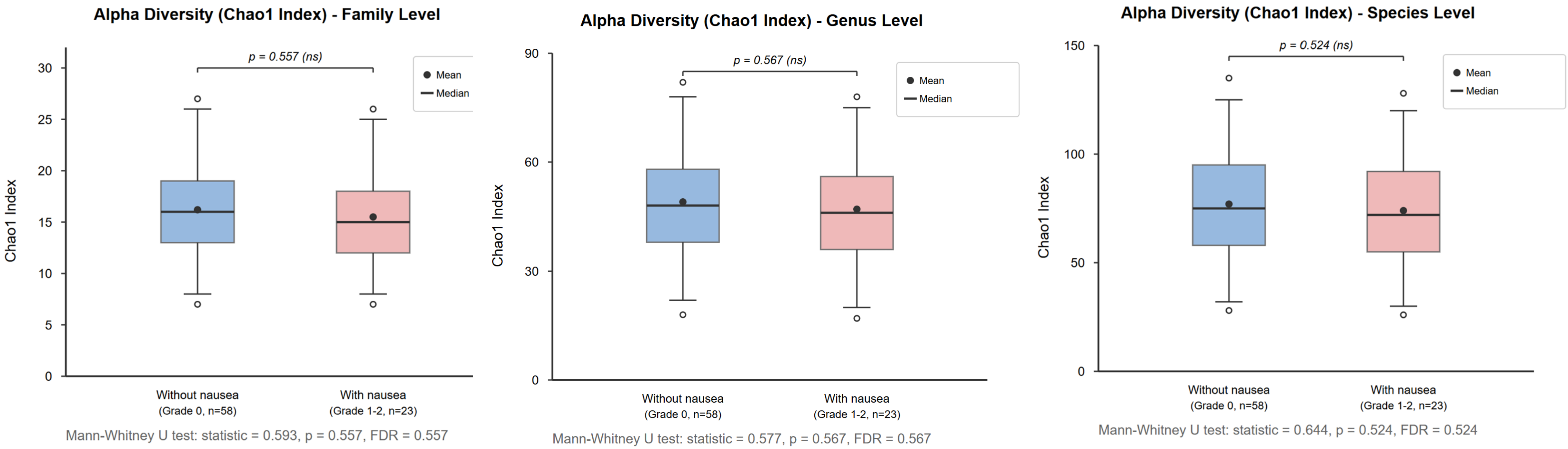

Supplementary Fig S6b

Beta Diversity Analysis: Group F

Principal Coordinates Analysis (PCoA)

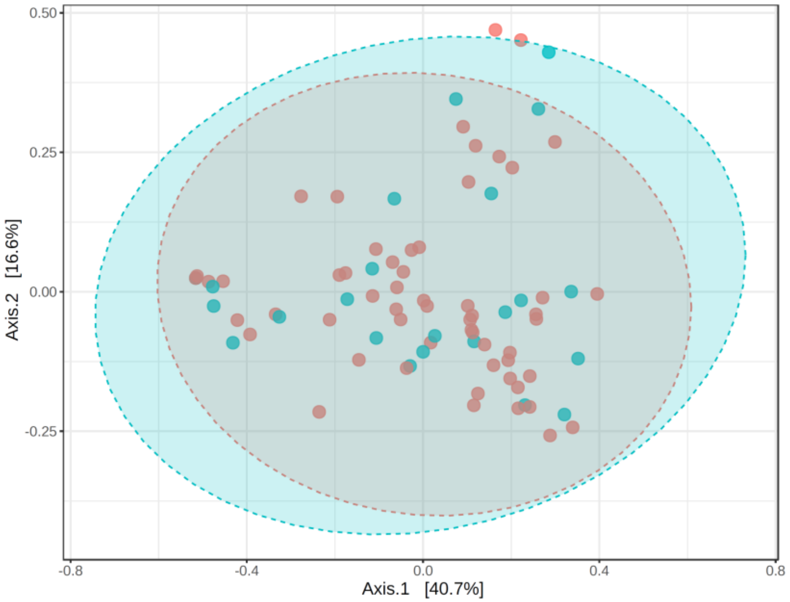

PERMANOVA Results

Comparison: 0 vs 1

Statistic	F	R-squared	P-value	FDR
0 vs 1	0.1408	0.0018	0.997	0.997

No significant difference between groups (P > 0.05)

Beta Diversity Analysis: Group G

Principal Coordinates Analysis (PCoA)

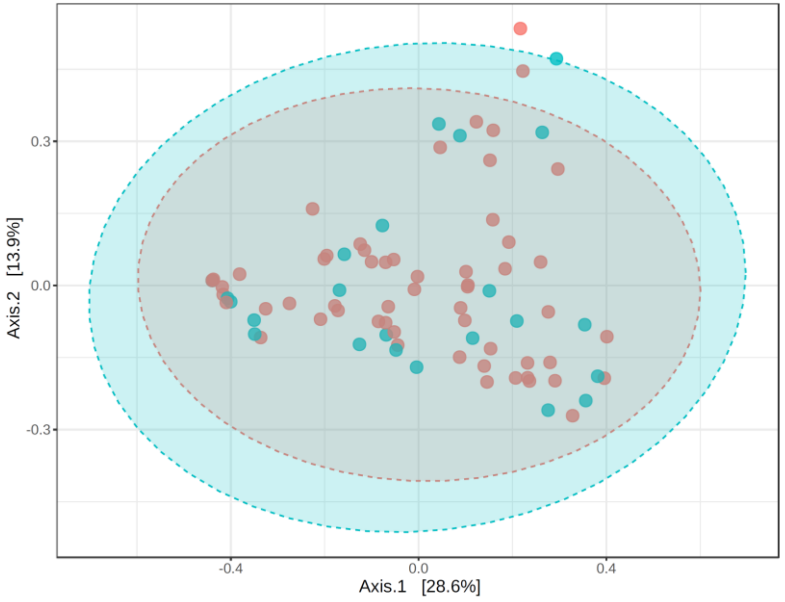

PERMANOVA Results

Comparison: 0 vs 1

Statistic	F	R-squared	P-value	FDR
0 vs 1	0.3350	0.0042	0.988	0.988

No significant difference between groups (P > 0.05)

Beta Diversity Analysis: Group S

Principal Coordinates Analysis (PCoA)

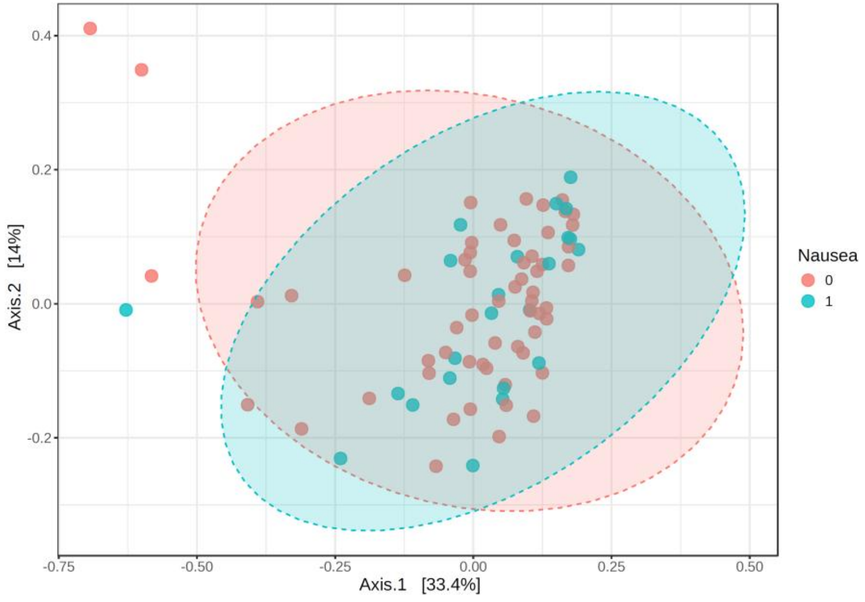

PERMANOVA Results

Comparison: 0 vs 1

Statistic	F	R-squared	P-value	FDR
0 vs 1	1.0654	0.0133	0.369	0.369

No significant difference between groups (P > 0.05)

Supplementary Fig S6c

Bacteroides coprocola DSM 17136

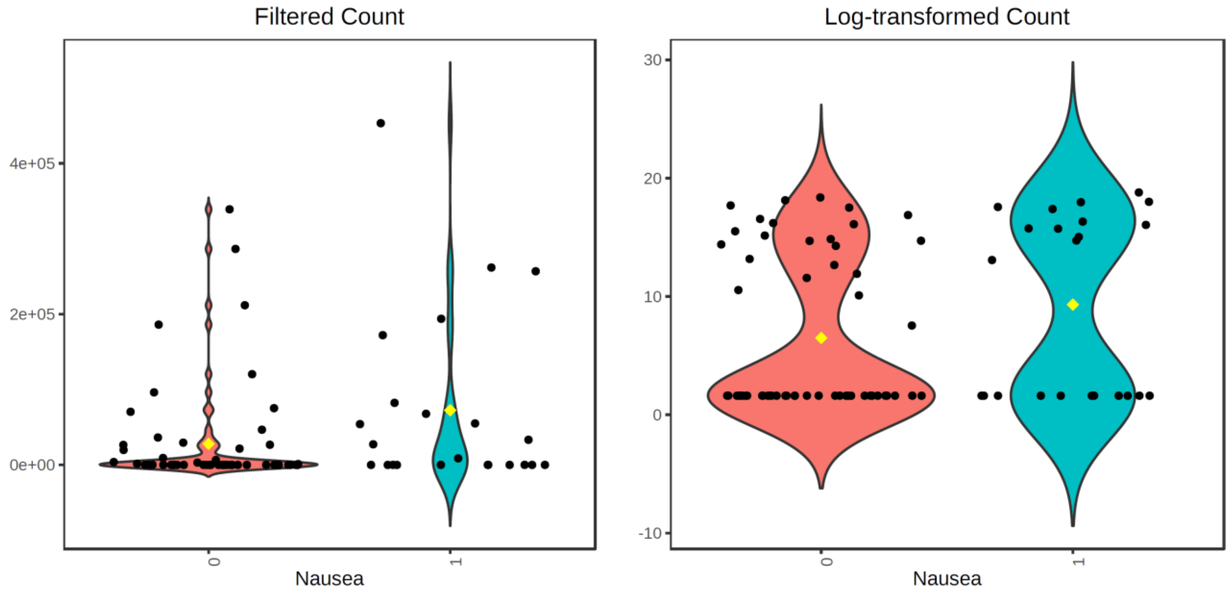

Intestinimonas butyriciproducens

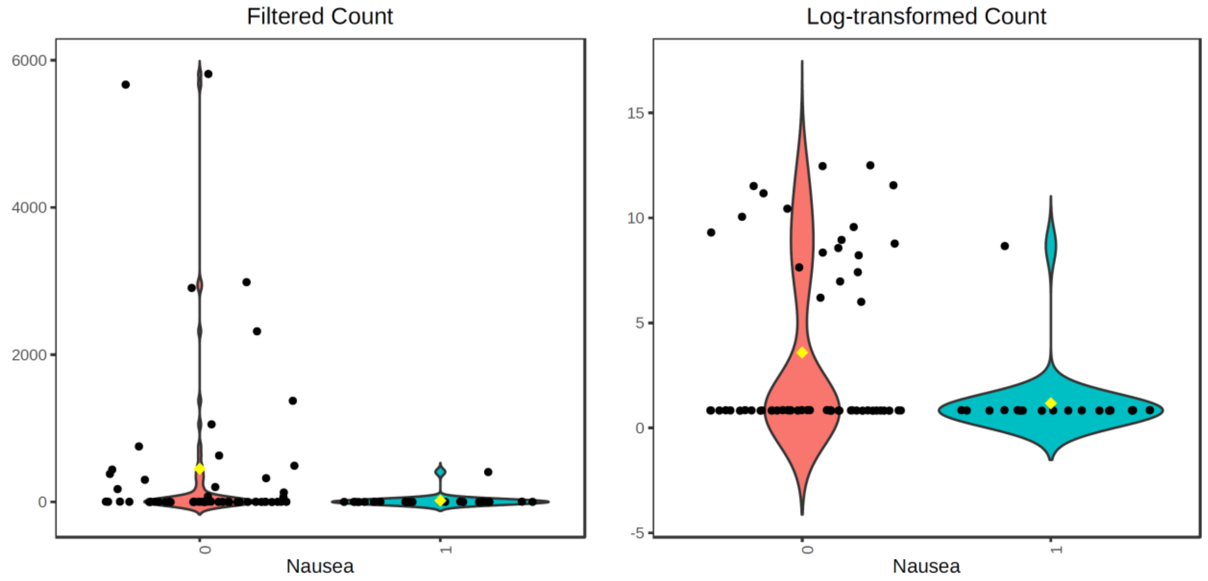

Supplementary Fig S7a

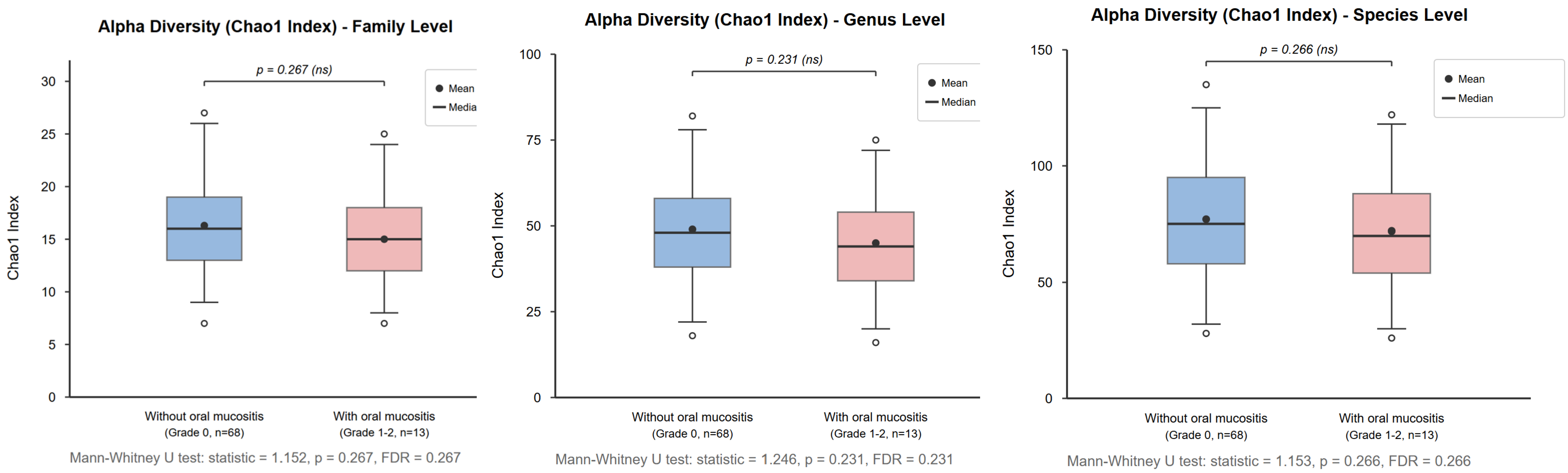

Supplementary Fig S7b

Beta Diversity Analysis: Group F

Principal Coordinates Analysis (PCoA)

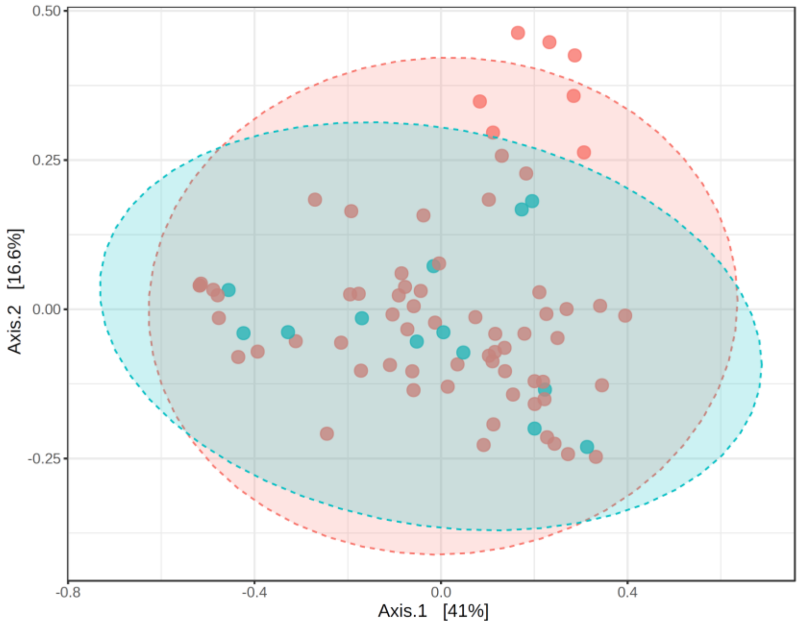

PERMANOVA Results

Comparison: 0 vs 1

Statistic	F	R-squared	P-value	FDR
0 vs 1	0.5854	0.0074	0.717	0.717

No significant difference between groups (P > 0.05)

Beta Diversity Analysis: Group G

Principal Coordinates Analysis (PCoA)

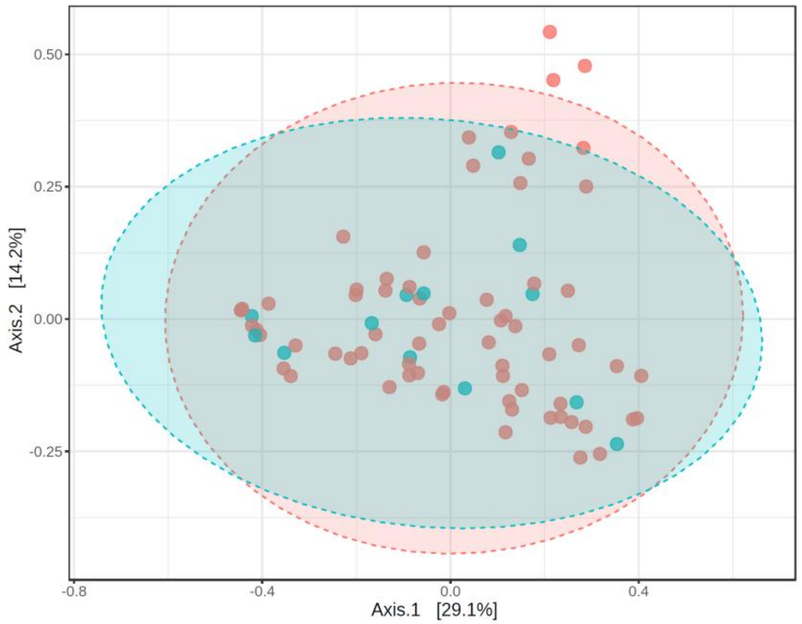

PERMANOVA Results

Comparison: 0 vs 1

Statistic	F	R-squared	P-value	FDR
0 vs 1	1.1061	0.0138	0.348	0.348

No significant difference between groups (P > 0.05)

Beta Diversity Analysis: Group S

Principal Coordinates Analysis (PCoA)

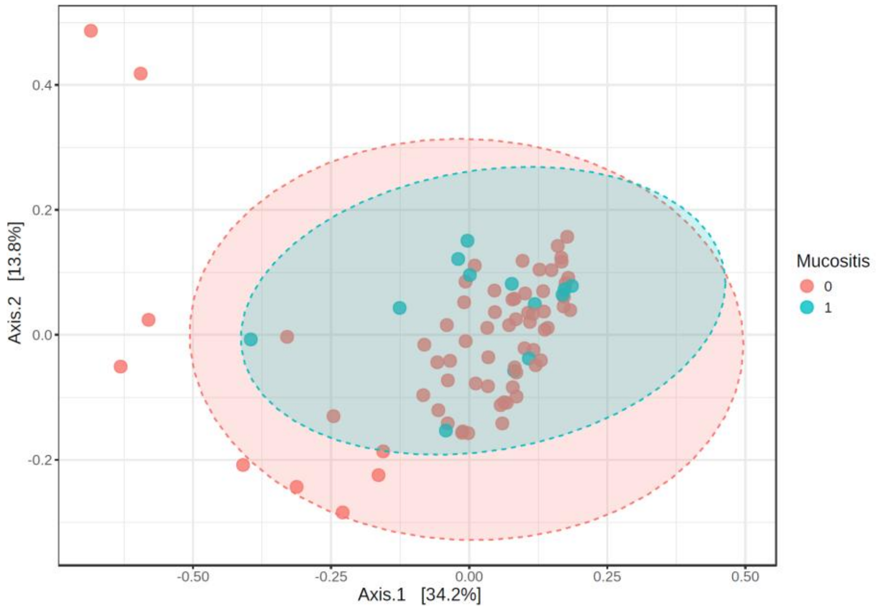

PERMANOVA Results

Comparison: 0 vs 1

Statistic	F	R-squared	P-value	FDR
0 vs 1	0.8149	0.0102	0.581	0.581

No significant difference between groups (P > 0.05)

Supplementary Fig S7c

Coprococcus_3

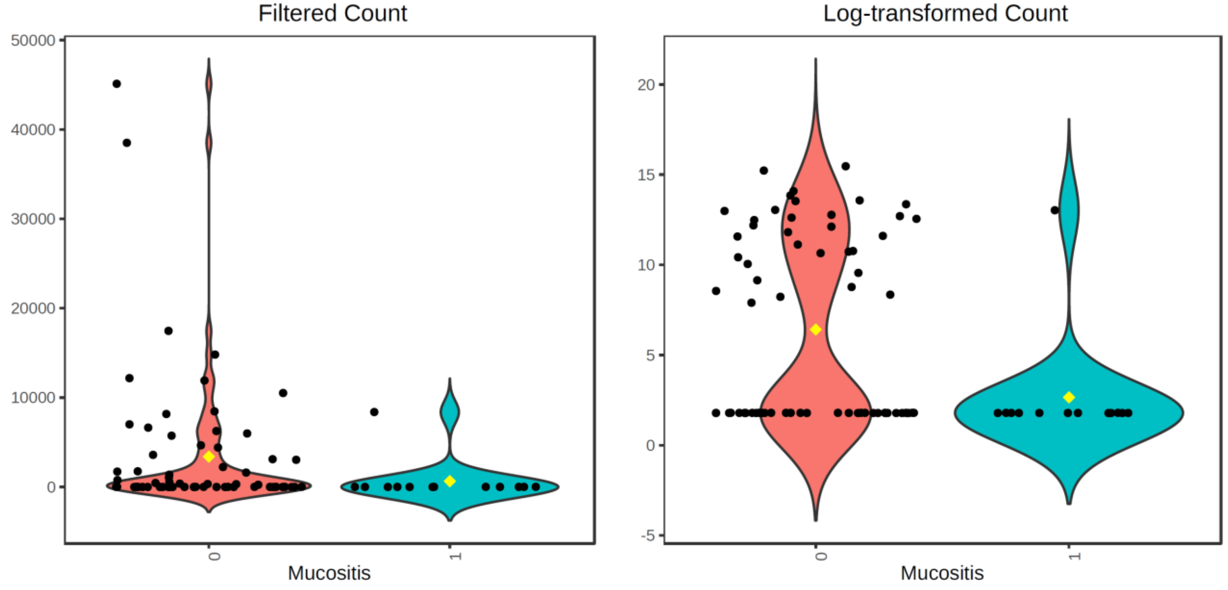

Ruminococcaceae UCG 014

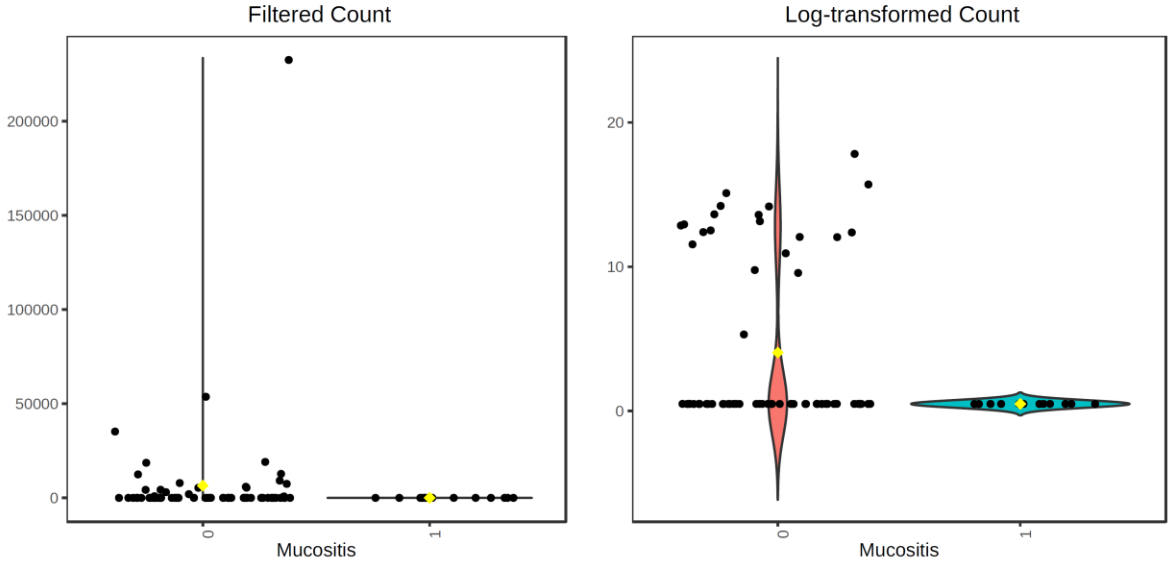

Gut metagenome

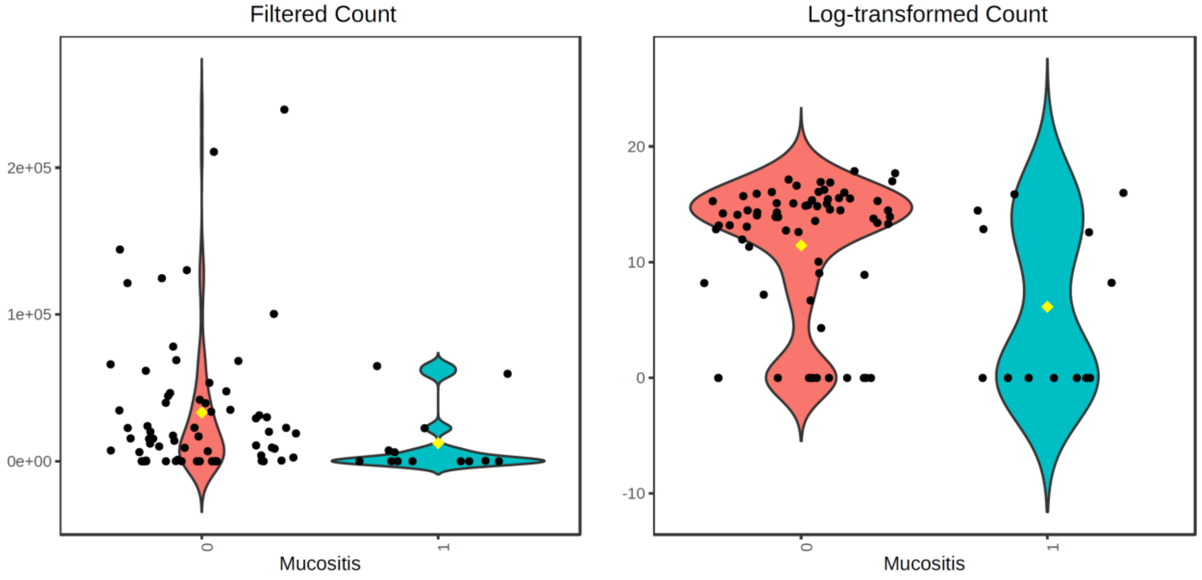

Supplementary Fig S8a

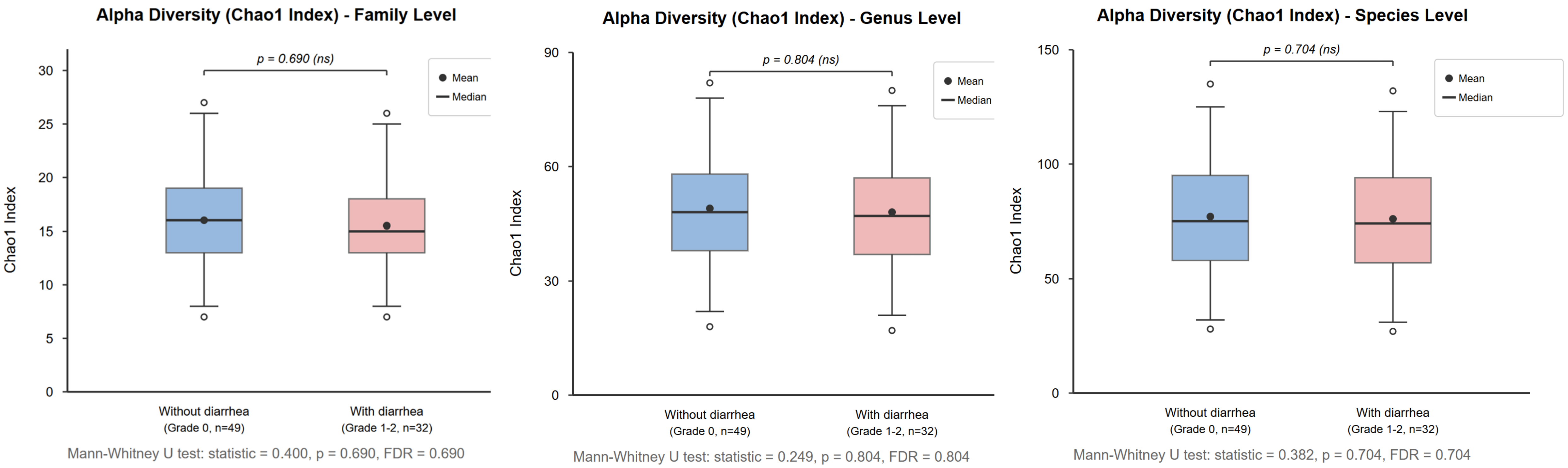

Supplementary Fig S8b

Beta Diversity Analysis: Group F

Principal Coordinates Analysis (PCoA)

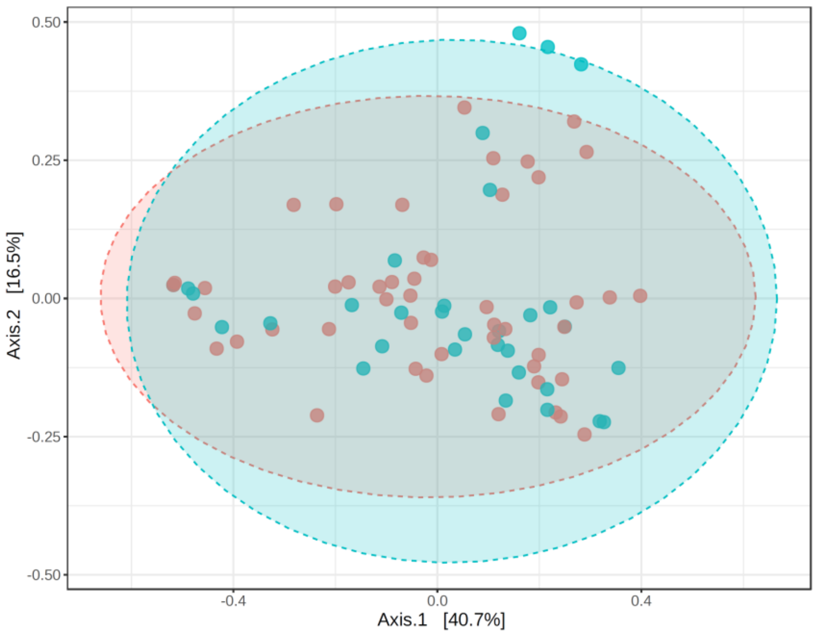

PERMANOVA Results

Comparison: 0 vs 1

Statistic	F	R-squared	P-value	FDR
0 vs 1	1.3489	0.0168	0.209	0.209

No significant difference between groups (P > 0.05)

Beta Diversity Analysis: Group G

Principal Coordinates Analysis (PCoA)

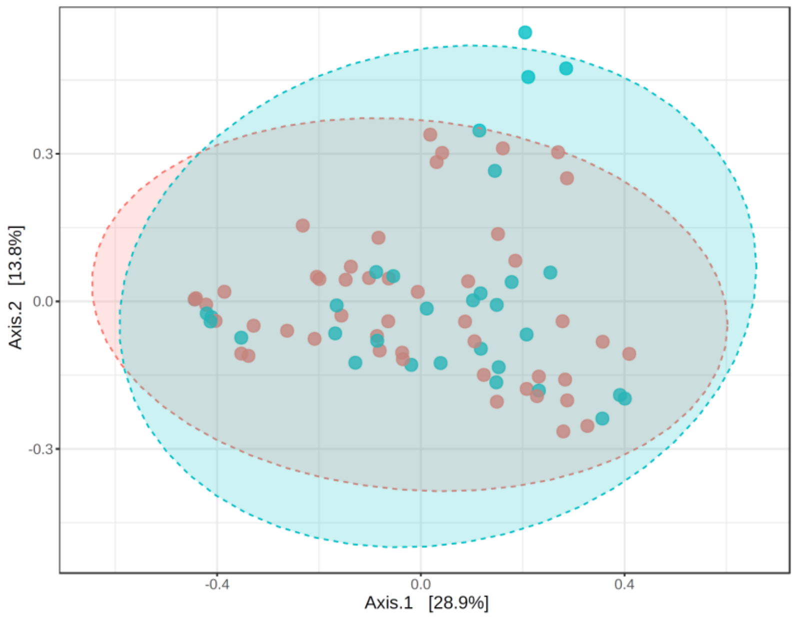

PERMANOVA Results

Comparison: 0 vs 1

Statistic	F	R-squared	P-value	FDR
0 vs 1	1.1742	0.0146	0.274	0.274

No significant difference between groups (P > 0.05)

Beta Diversity Analysis: Group S

Principal Coordinates Analysis (PCoA)

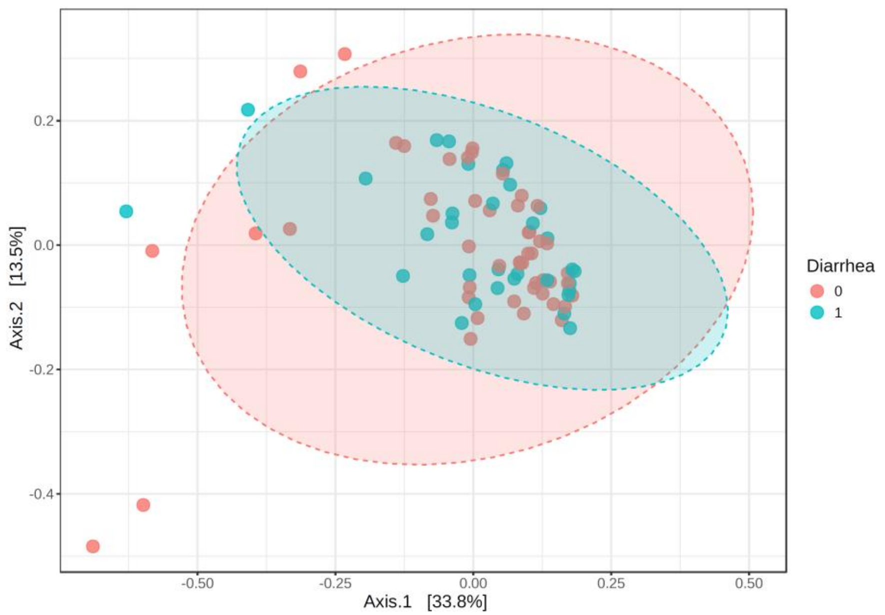

PERMANOVA Results

Comparison: 0 vs 1

Statistic	F	R-squared	P-value	FDR
0 vs 1	1.1825	0.0147	0.311	0.311

No significant difference between groups (P > 0.05)

Supplementary Fig S8c

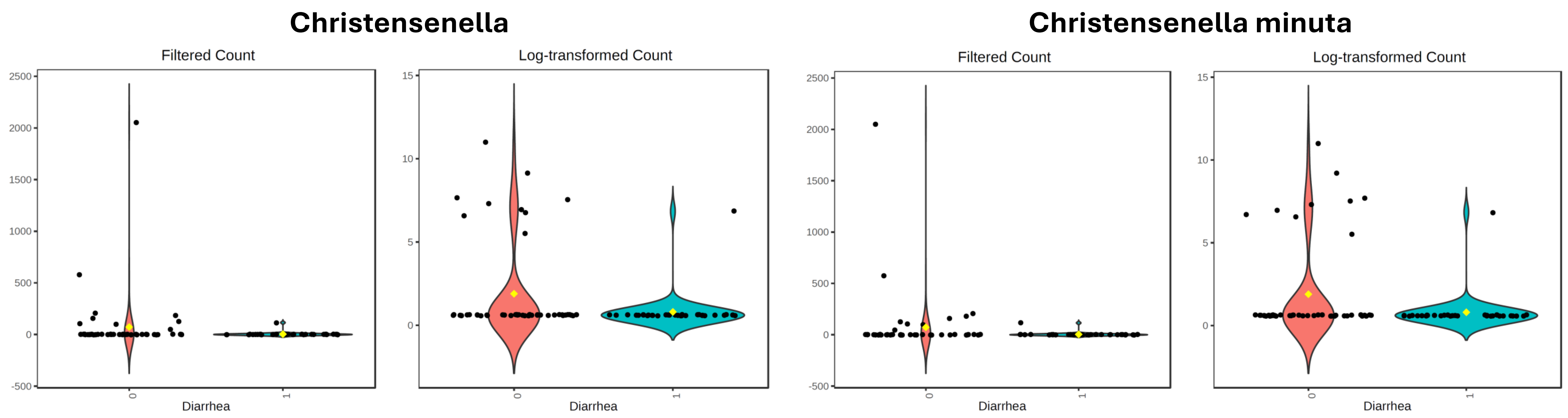

Supplementary Fig S9a

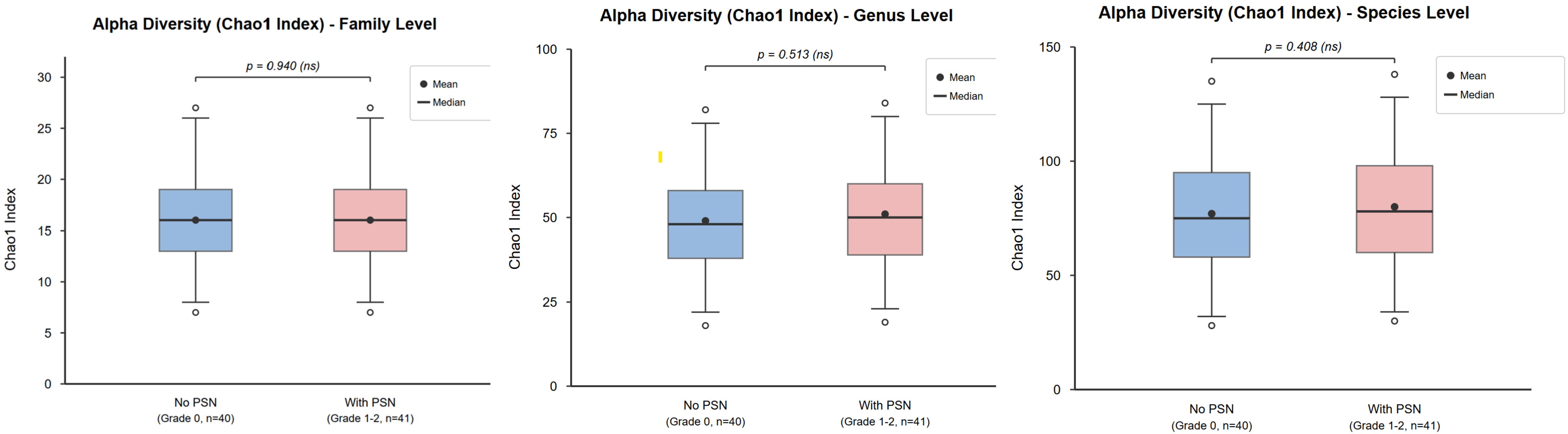

Mann-Whitney U test: statistic = 0.075, p = 0.940, FDR = 0.940

Mann-Whitney U test: statistic = -0.657, p = 0.513, FDR = 0.513

Mann-Whitney U test: statistic = -0.832, p = 0.408, FDR = 0.408

Supplementary Fig S9b

Beta Diversity Analysis: Group F

Principal Coordinates Analysis (PCoA)

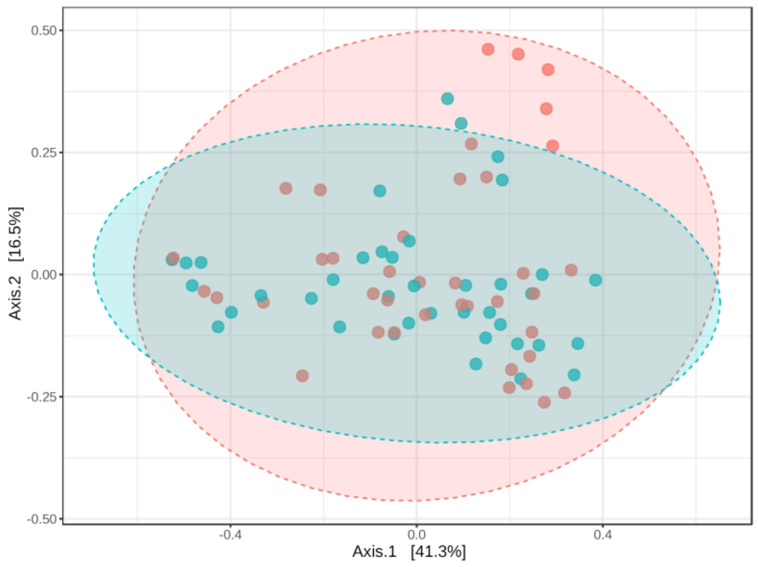

PERMANOVA Results

Comparison: 0 vs 1

Statistic	F	R-squared	P-value	FDR
0 vs 1	0.8679	0.0110	0.506	0.506

No significant difference between groups (P > 0.05)

Beta Diversity Analysis: Group G

Principal Coordinates Analysis (PCoA)

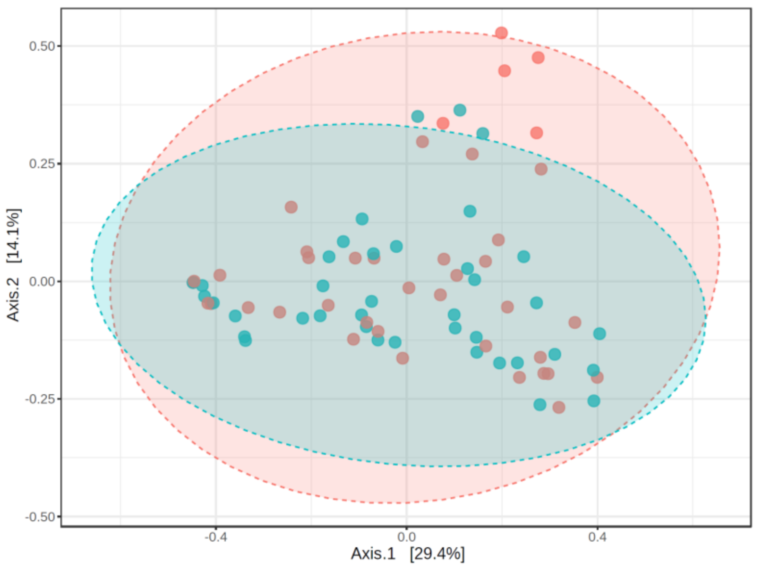

PERMANOVA Results

Comparison: 0 vs 1

Statistic	F	R-squared	P-value	FDR
0 vs 1	1.1515	0.0145	0.301	0.301

No significant difference between groups (P > 0.05)

Beta Diversity Analysis: Group S

Principal Coordinates Analysis (PCoA)

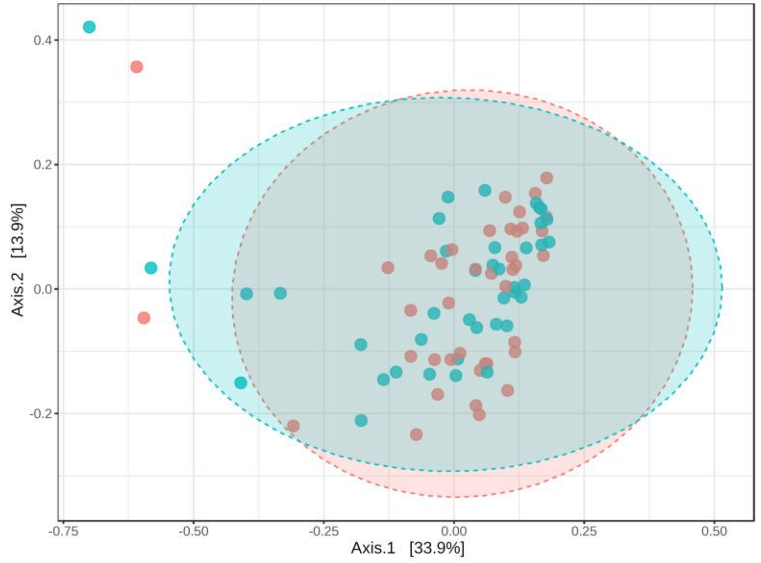

PERMANOVA Results

Comparison: 0 vs 1

Statistic	F	R-squared	P-value	FDR
0 vs 1	0.9127	0.0116	0.507	0.507

No significant difference between groups (P > 0.05)

Supplementary Fig S9c

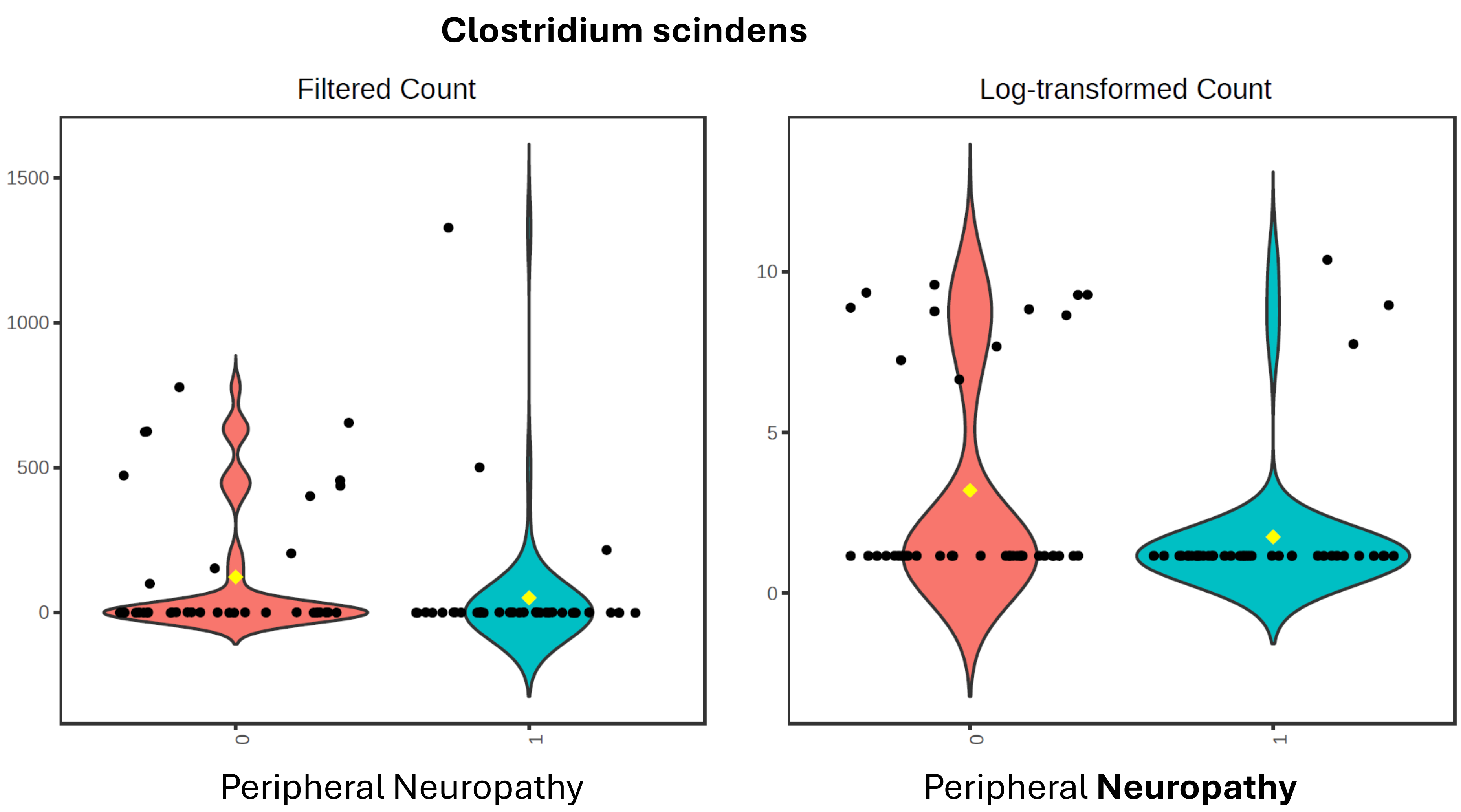

Supplementary Fig S10a

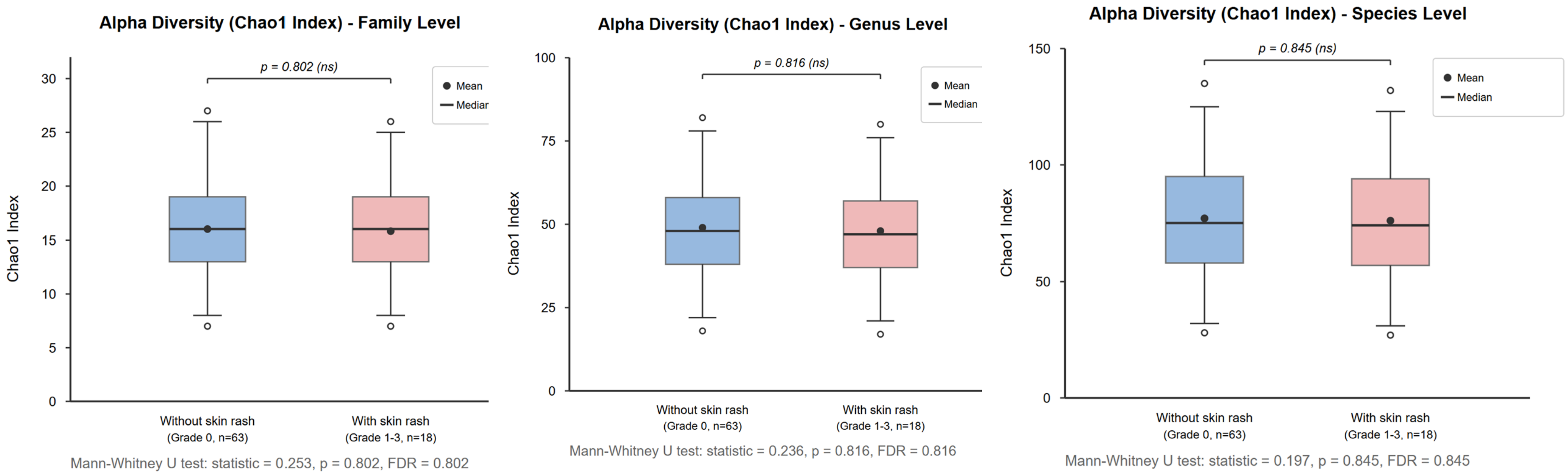

Supplementary Fig S10b

Beta Diversity Analysis: Group F

Principal Coordinates Analysis (PCoA)

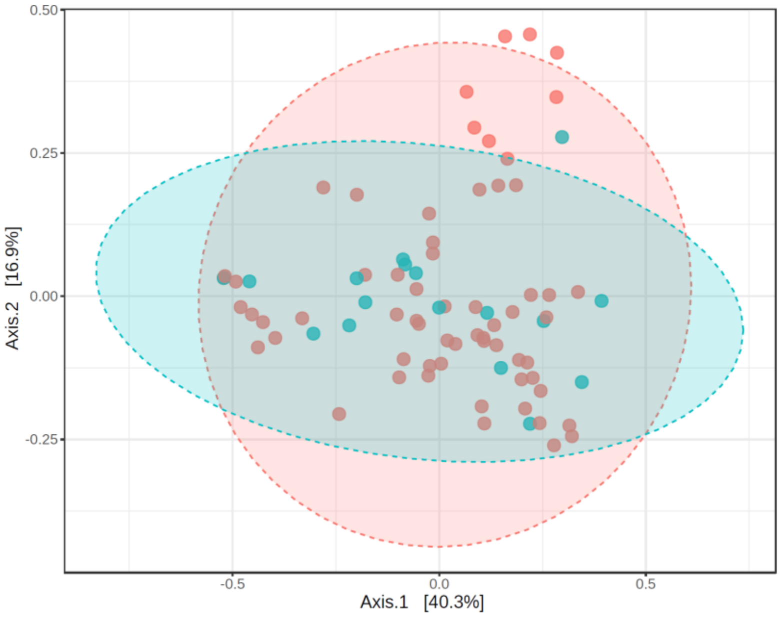

PERMANOVA Results

Comparison: 0 vs 1

Statistic	F	R-squared	P-value	FDR
0 vs 1	0.7969	0.0100	0.569	0.569

No significant difference between groups (P > 0.05)

Beta Diversity Analysis: Group G

Principal Coordinates Analysis (PCoA)

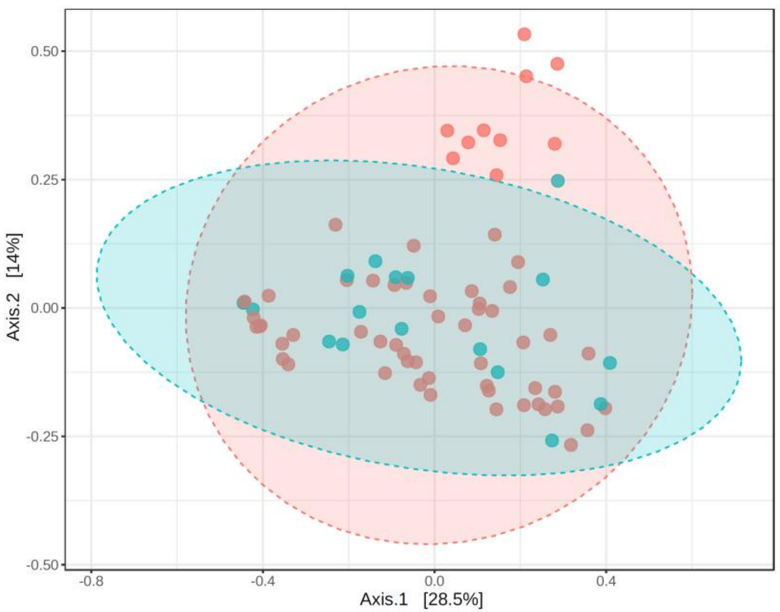

PERMANOVA Results

Comparison: 0 vs 1

Statistic	F	R-squared	P-value	FDR
0 vs 1	0.9914	0.0124	0.448	0.448

No significant difference between groups (P > 0.05)

Beta Diversity Analysis: Group S

Principal Coordinates Analysis (PCoA)

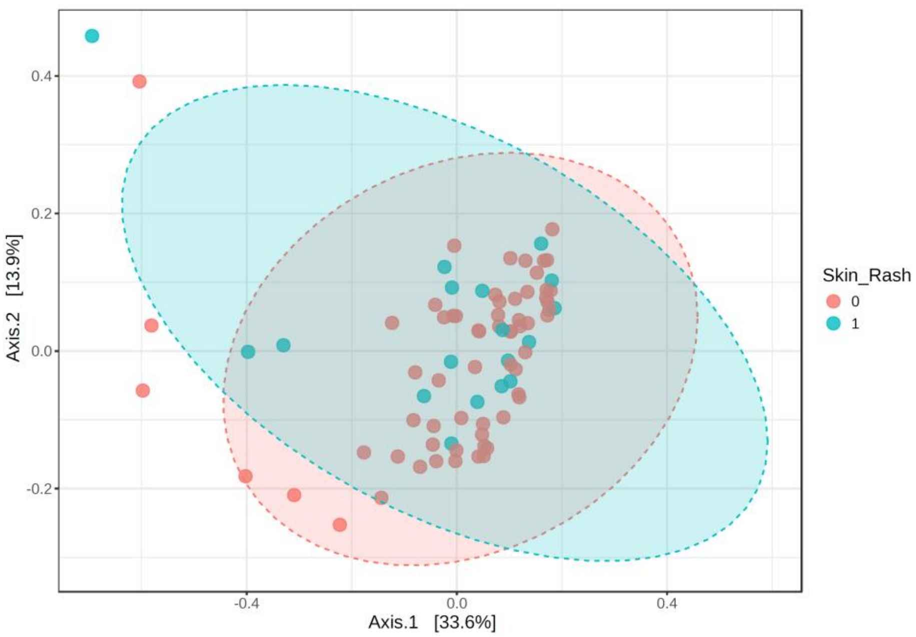

PERMANOVA Results

Comparison: 0 vs 1

Statistic	F	R-squared	P-value	FDR
0 vs 1	1.3514	0.0168	0.222	0.222

No significant difference between groups (P > 0.05)

Supplementary Fig S10c

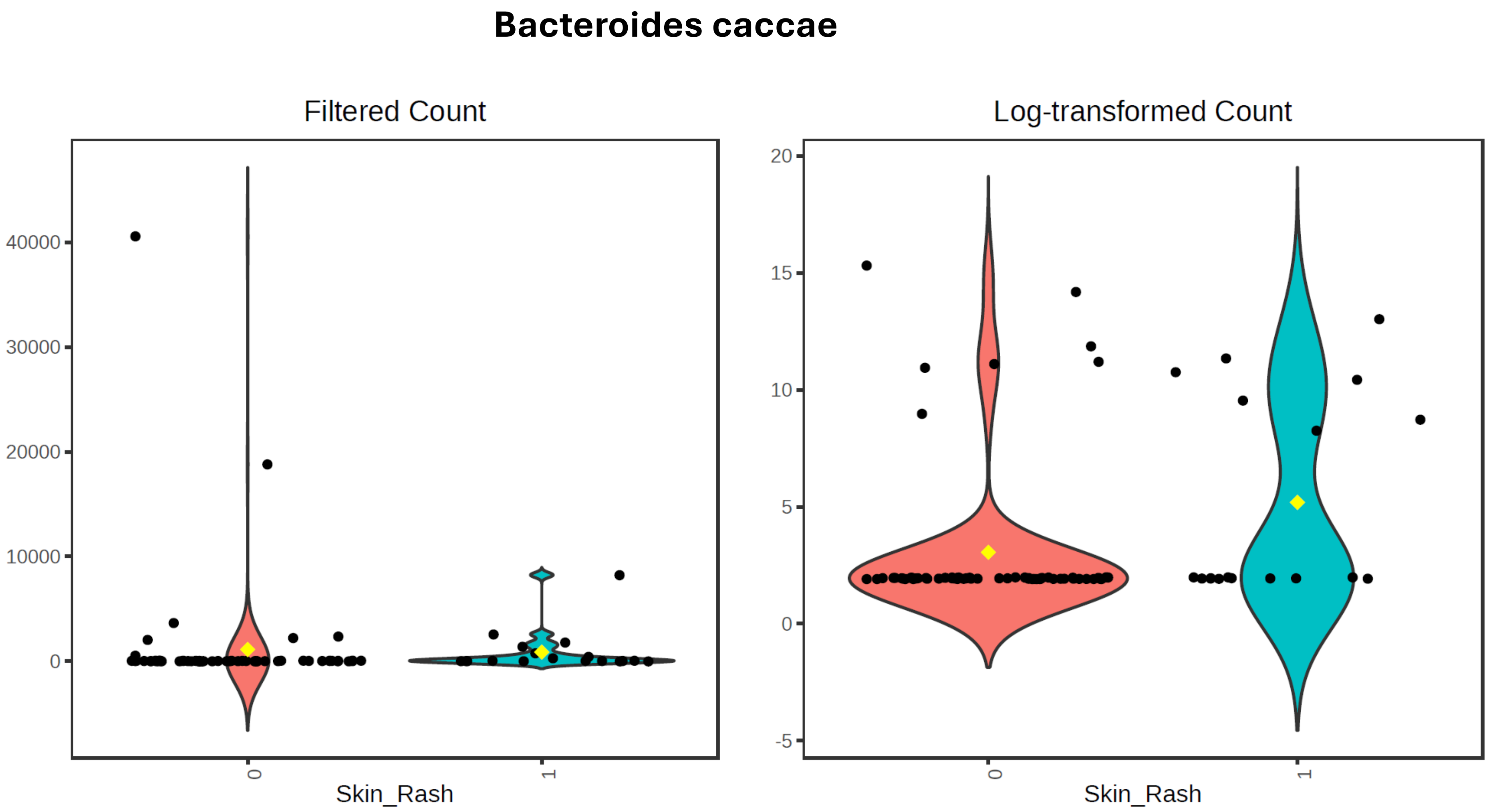

Supplementary Fig S11a

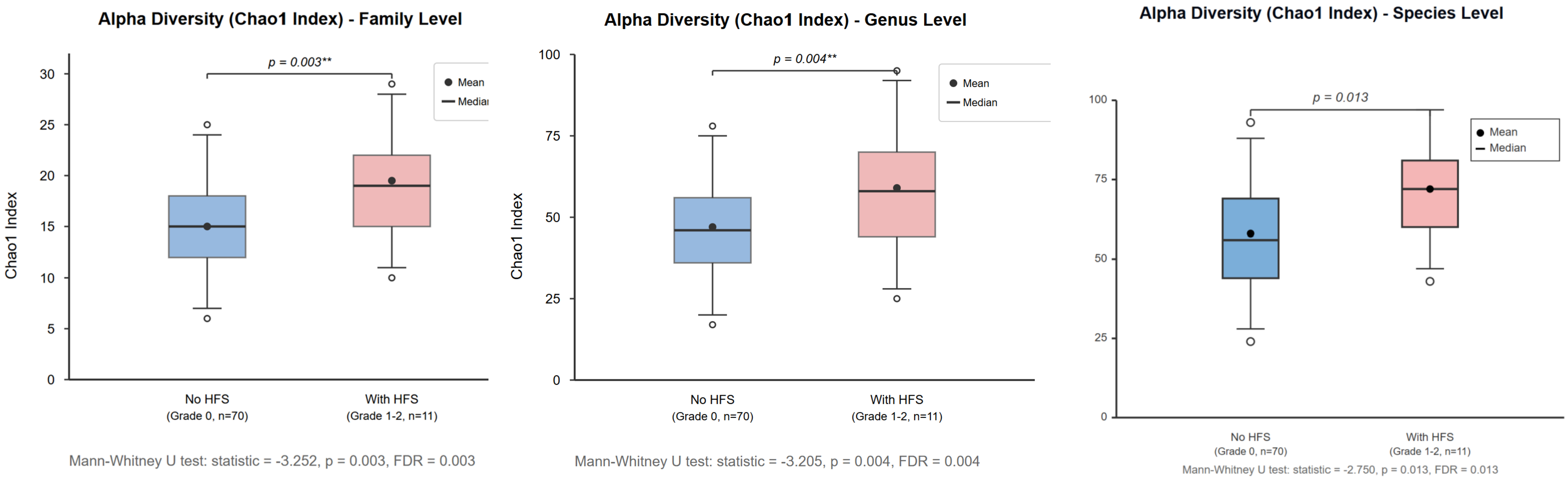

Supplementary Fig S11b

Beta Diversity Analysis: Group F

Principal Coordinates Analysis (PCoA)

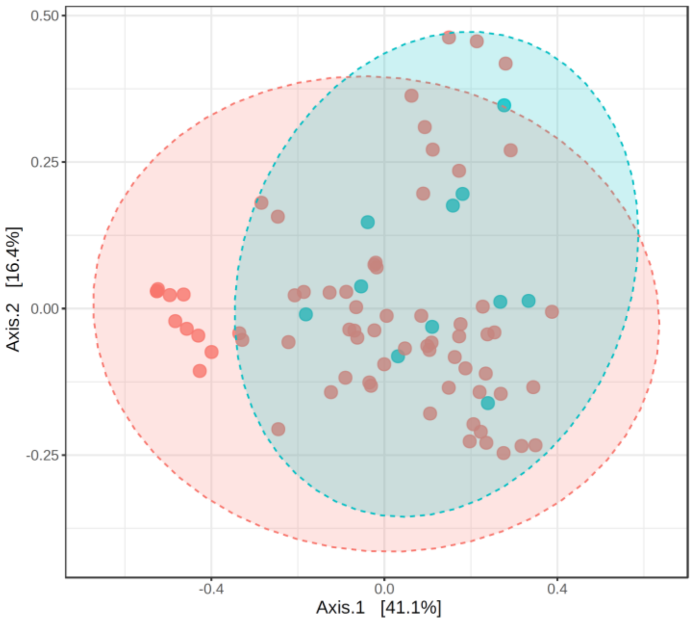

PERMANOVA Results

Comparison: 0 vs 1

Statistic	F	R-squared	P-value	FDR
0 vs 1	2.2510	0.0277	**0.051**	0.051

Borderline significance (P = 0.051), trending toward significance

Beta Diversity Analysis: Group G

Principal Coordinates Analysis (PCoA)

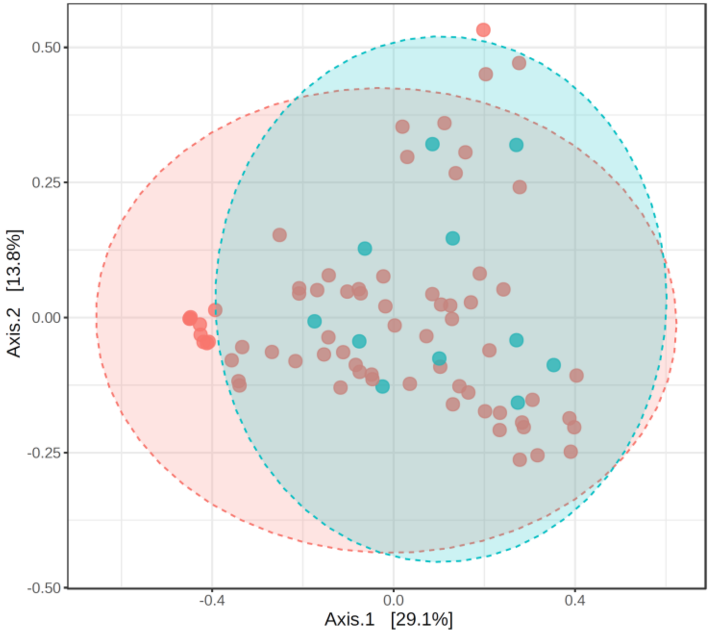

PERMANOVA Results

Comparison: 0 vs 1

Statistic	F	R-squared	P-value	FDR
0 vs 1	1.7170	0.0213	0.084	0.084

Not significant (P = 0.084), but shows a trend

Beta Diversity Analysis: Group S

Principal Coordinates Analysis (PCoA)

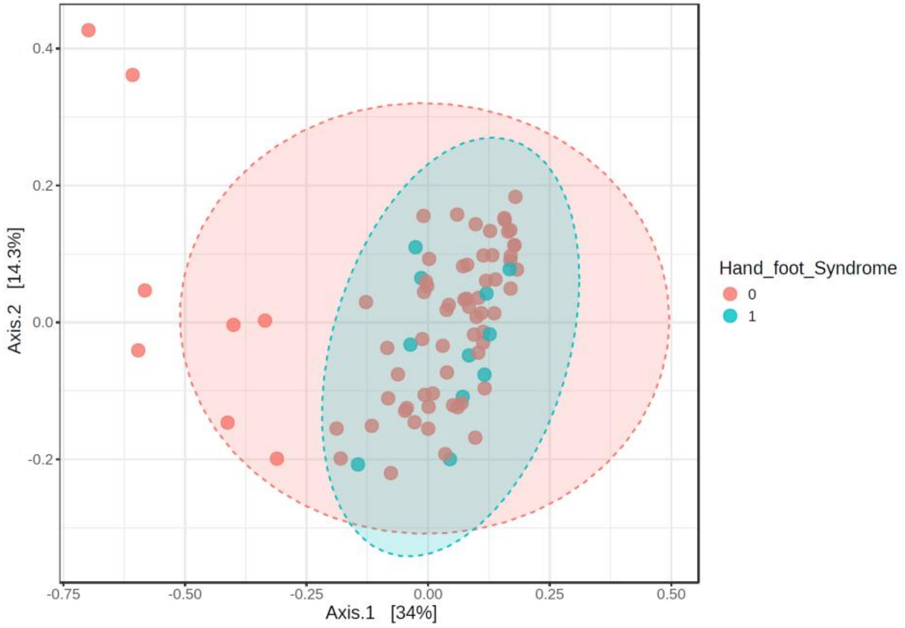

PERMANOVA Results

Comparison: 0 vs 1

Statistic	F	R-squared	P-value	FDR
0 vs 1	1.0100	0.0126	0.356	0.356

No significant difference between groups (P > 0.05)

Supplementary Fig S11c

Eggerthellaceae

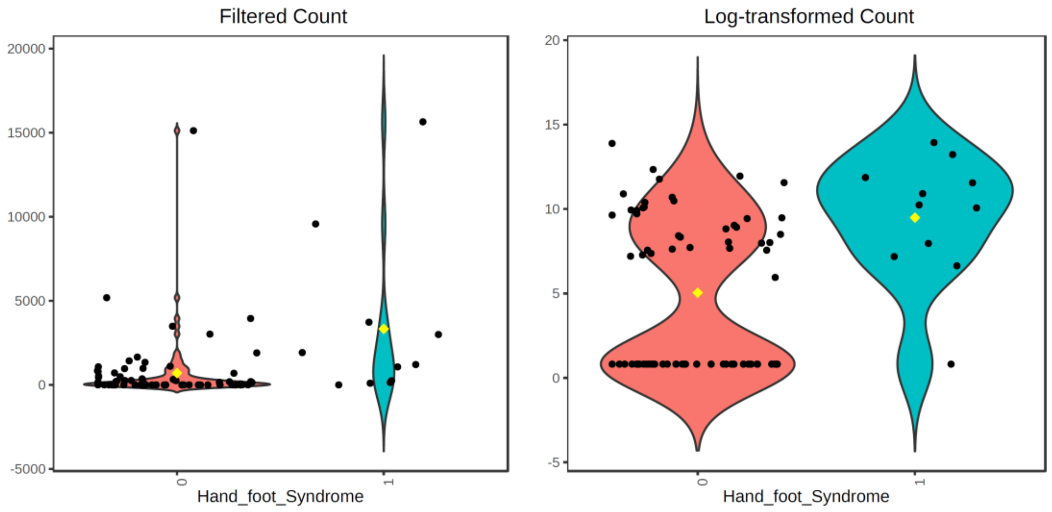

Butyricicoccus

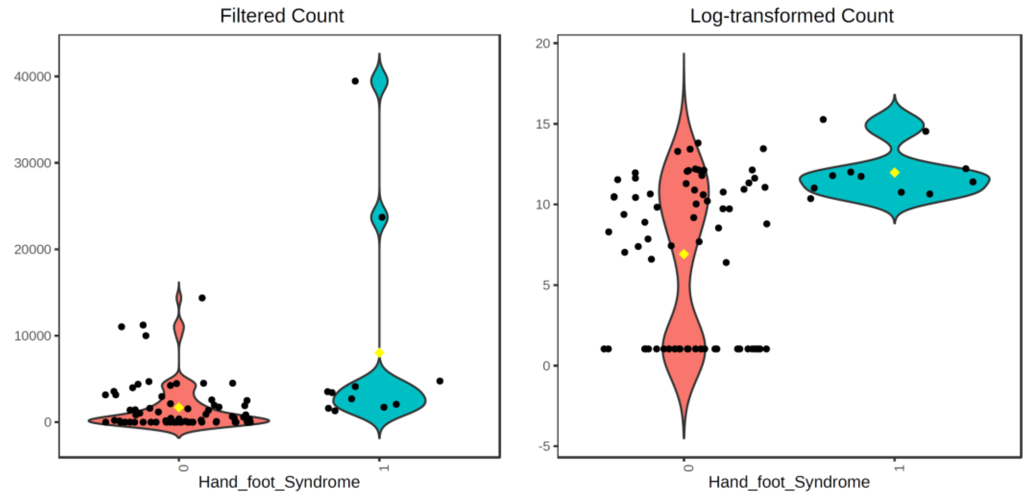

Lachnospiraceae

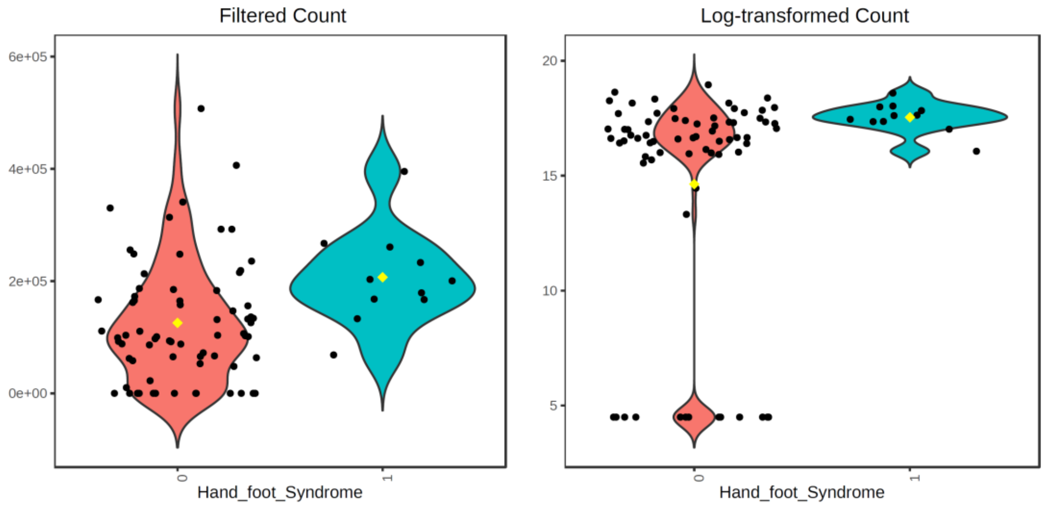

Roseburia

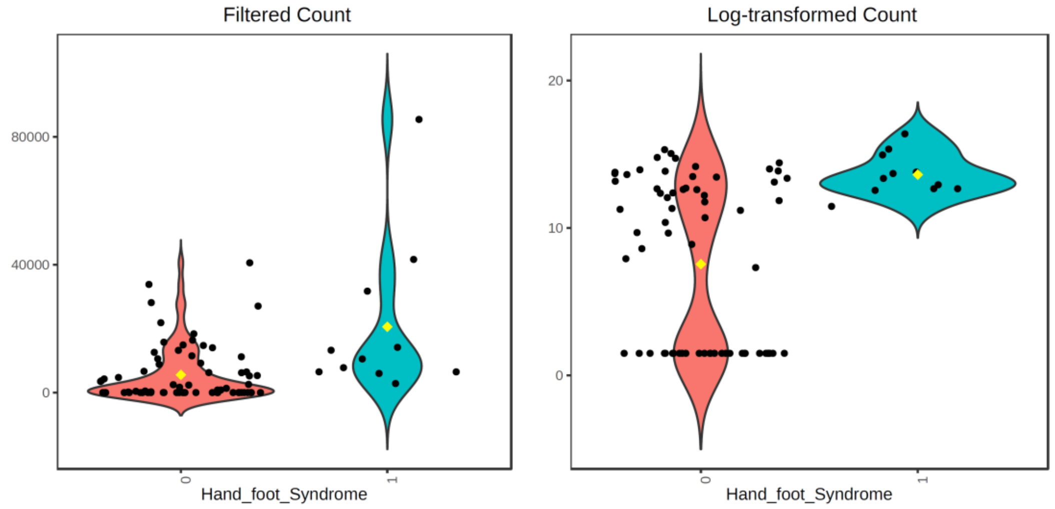

Supplementary Fig S12a

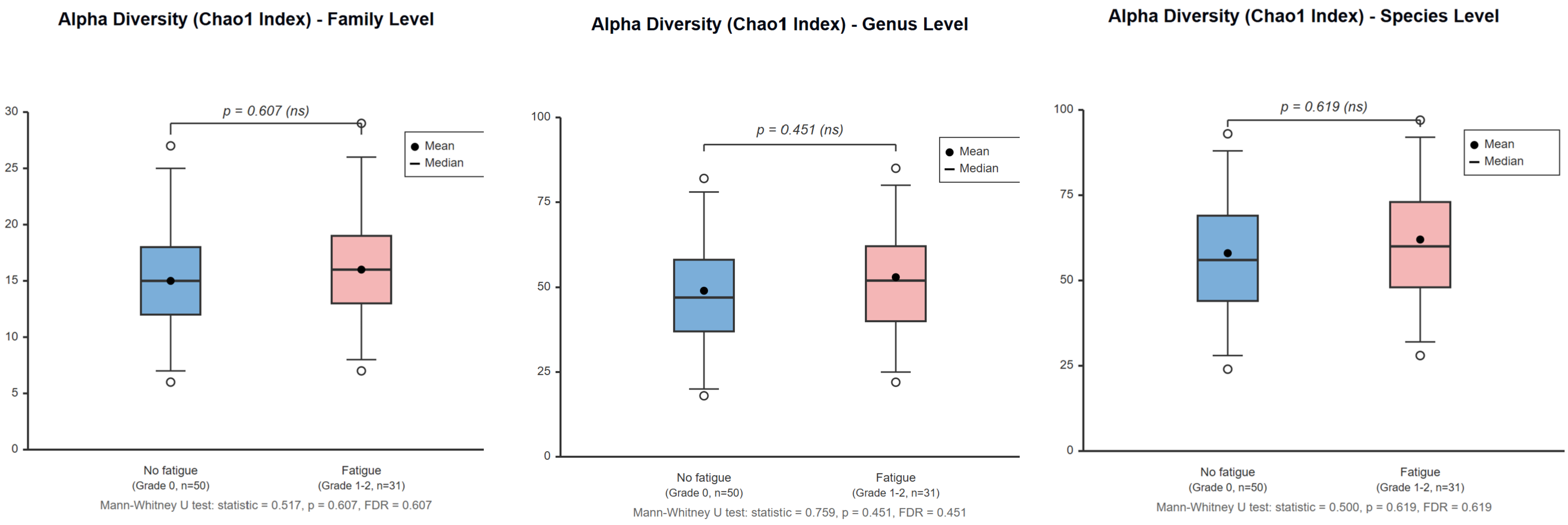

Supplementary Fig S12b

Beta Diversity Analysis: Group F

Principal Coordinates Analysis (PCoA)

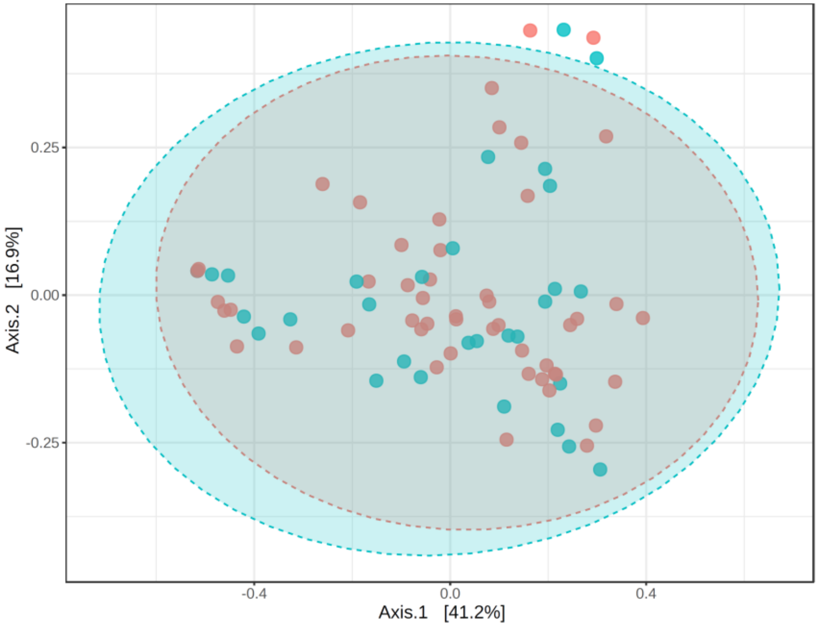

PERMANOVA Results

Comparison: 0 vs 1

Statistic	F	R-squared	P-value	FDR
0 vs 1	0.4046	0.0051	0.877	0.877

No significant difference between groups (P > 0.05)

Beta Diversity Analysis: Group G

Principal Coordinates Analysis (PCoA)

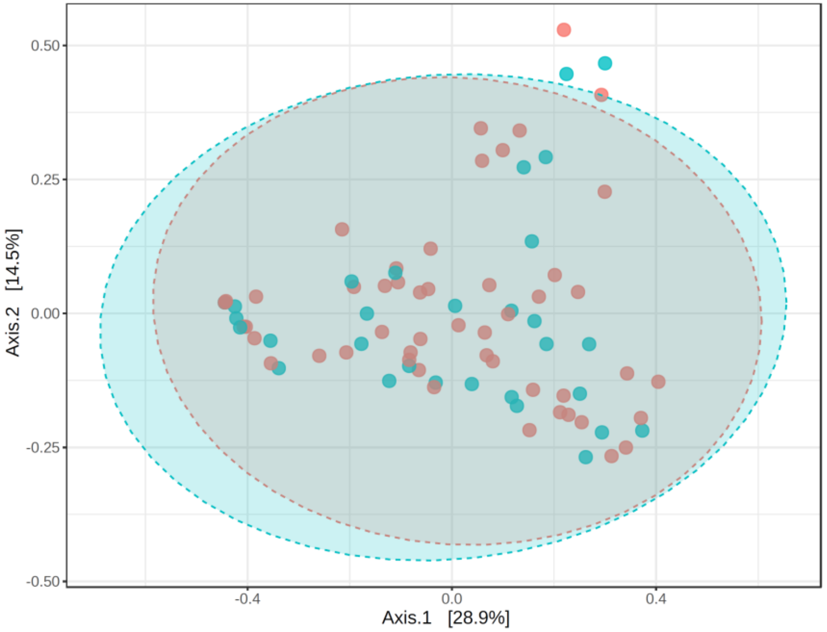

PERMANOVA Results

Comparison: 0 vs 1

Statistic	F	R-squared	P-value	FDR
0 vs 1	0.4257	0.0054	0.964	0.964

No significant difference between groups (P > 0.05)

Beta Diversity Analysis: Group S

Principal Coordinates Analysis (PCoA)

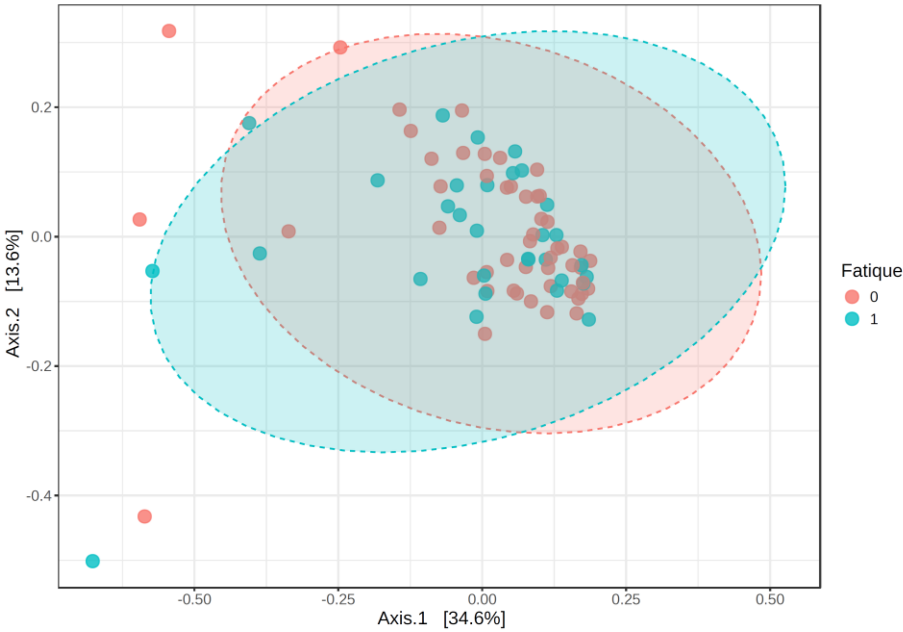

PERMANOVA Results

Comparison: 0 vs 1

Statistic	F	R-squared	P-value	FDR
0 vs 1	0.9417	0.0118	0.466	0.466

No significant difference between groups (P > 0.05)

Supplementary Fig S12c

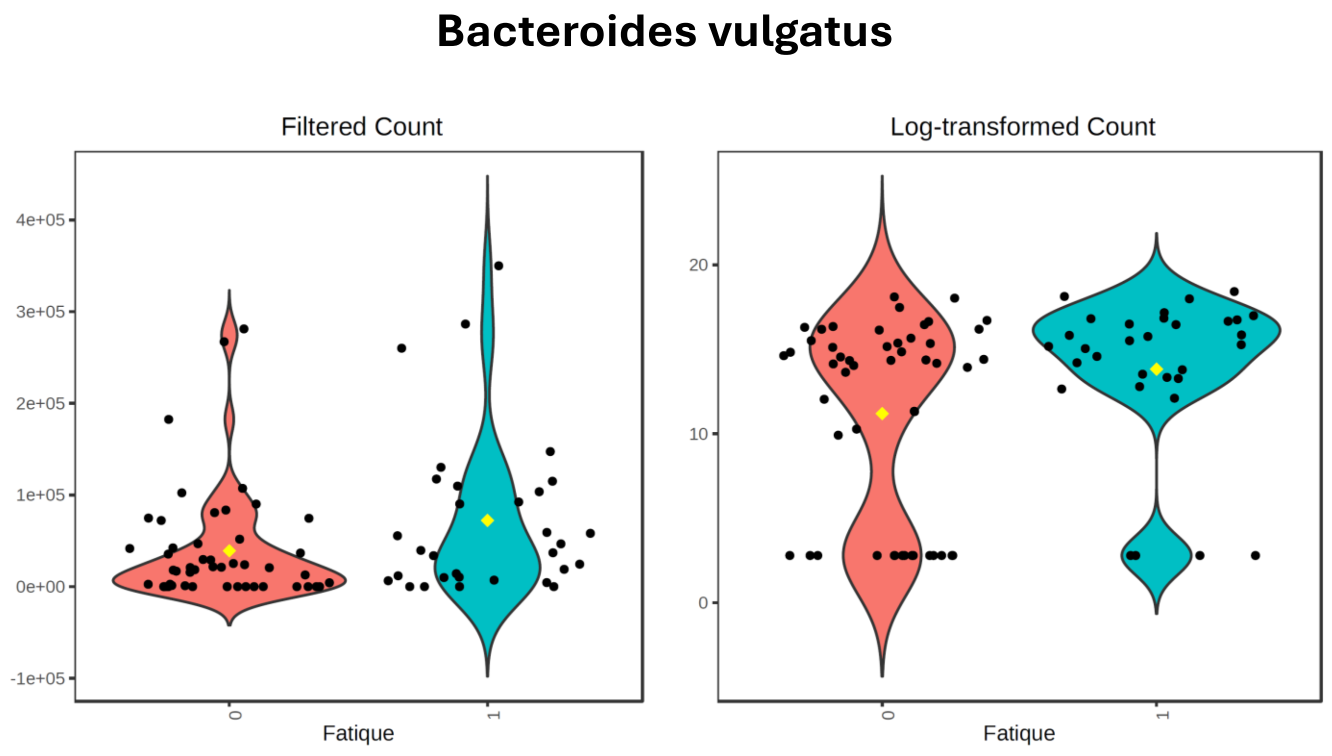

Supplement: Supplementary file 1 [file cancers-17-03783-s001.zip › cancers-3971723-supplementary.pdf]
